# Census-Tract-Level Median Household Income and Median Family Income Estimates: A Unidimensional Measure of Neighborhood Socioeconomic Status?

**DOI:** 10.3390/ijerph20010211

**Published:** 2022-12-23

**Authors:** Masayoshi Oka

**Affiliations:** Department of Management, Faculty of Management, Josai University, Sakado 350-0295, Japan; moka@alumni.harvard.edu

**Keywords:** neighborhood socioeconomic status, neighborhood deprivation, small-area socioeconomic status, small-area deprivation, conterminous United States

## Abstract

Previous studies suggested either census-tract-level median household income (MHI) or median family income (MFI) estimates may be used as a unidimensional measure of neighborhood socioeconomic status (SES) in the United States (US). To better understand its general use, the purpose of this study was to assess the usefulness of MHI and MFI in a wide range of geographic areas. Area-based socioeconomic data at the census tract level were obtained from the 2000 Census as well as the 2005–2009, 2010–2014, and 2015–2019 American Community Survey. MHI and MFI were used as two simple measures of neighborhood SES. Based on the five area-based indexes developed in the US, several census-tract-level socioeconomic indicators were used to derive five composite measures of neighborhood SES. Then, a series of correlation analyses was conducted to assess the relationships between these seven measures in the State of California and its seven Metropolitan Statistical Areas. Two simple measures were very strongly and positively correlated with one another, and were also strongly or very strongly correlated, either positively or negatively, with five composite measures. Hence, the results of this study support an analytical thinking that simple measures and composite measures may capture the same dimension of neighborhood SES in different geographic areas.

## 1. Introduction

Neighborhood social and/or physical characteristics may shape the health of its residents over and above their socio-demographic characteristics (e.g., age, gender, race/ethnicity, marital status, educational attainment, income level, and occupational status) [1,2]. Drawing from a large number of research studies conducted in the United States (US) and other countries, several review articles concluded that lower neighborhood socioeconomic status (SES) have been consistently associated with poor health, such as cardiovascular disease or coronary heart disease [3,4], depression [5,6], obesity and physical inactivity [7,8,9], substance use [10], mortality [11], adverse perinatal outcomes [12], physical aggression in children and adolescents [13], worse cognition in older adults [14], and sleep problems among children and adolescents [15]. Since the mid-2000s, multilevel (or hierarchical) regression models [16,17,18,19] have been used to combine traditionally distinct individual and ecological models, and to overcome analytical limitations inherent in focusing only on one level, in these reviewed studies. Note that the terms “neighborhood SES” and “neighborhood deprivation” appear most frequently in previous studies and have been conceived as synonyms to refer to the same dimension of neighborhood characteristics. Regardless of the different terminologies used in the literature, these review articles [3,4,5,6,7,8,9,10,11,12,13,14,15] highlighted the importance of understanding where people live in health research.

In previous studies [3,4,5,6,7,8,9,10,11,12,13,14,15], census tracts (also referred to as census areas, census districts, or census divisions outside the US) have been used to denote neighborhoods, and area-level socioeconomic data from national population and housing censuses have been compiled into an area-based index to derive a composite measure (i.e., a unidimensional measure) of neighborhood SES (or deprivation). For example, the area-based indexes of socioeconomic advantage (SEA) [20], socioeconomic position (SEP) [21], socioeconomic deprivation (SED) [22], socioeconomic status (SES) [23], and deprivation (DEP) [24] have been developed in the US (Table 1) and have been commonly used in US studies. To derive a composite measure of neighborhood SES (or deprivation), several area-level socioeconomic indictors are combined into an unweighted or weighted summary score either by a sum of z-scores, factor analysis, or principal component analysis. Different types of census-tract-level socioeconomic indicators and computational method used in each area-based index are listed in Table 1. Although the conceptual and methodological approach differs across area-based indexes (including those not listed in Table 1), similar findings across previous studies [3,4,5,6,7,8,9,10,11,12,13,14,15] corroborate a notion that a choice of area-based index may not matter much (i.e., “just pick one”) when examining an effect of neighborhood SES (or deprivation) on health in a multilevel analysis.

This notion of interchangeability has been supported by empirical studies [25,26] where multiple area-based indexes were used to derive composite measures of neighborhood SES (or deprivation) and their performance was examined in separate regression analyses by holding other covariates constant. Despite the analytical differences between these method comparison studies, a series of regression analyses yielded very similar results in a range of outcomes related to health [25,26]. Note that two or more strongly correlated measures (in general, a correlation coefficient [*r*] of greater than 0.80) in a regression analysis would lead to collinearity or multicollinearity, and thus replacing one another in separate regression analyses (holding other covariates constant) would yield very similar regression coefficients and standard errors. Building upon the basic principles of regression analysis, one study [27] suggested that either census-tract-level median household income (MHI) or median family income (MFI) may be used as a simple measure of neighborhood SES (or deprivation) without the need of any area-based index. This means that using either of the two forgoes the hassle of compiling multiple census-tract-level socioeconomic indicators and carrying out complicated computational analysis necessary for deriving a composite measure of neighborhood SES (or deprivation) [27]. To support this suggestion, MHI has been shown to yield very similar results as a composite measure of neighborhood SES (or deprivation) in an empirical study [28] and in a simulation study [29]. Taken together, these more recent method comparison studies [28,29] evince an analytical thinking that simple measures (i.e., MHI and MFI) and composite measures derived from area-based indexes (e.g., SEA, SEP, SED, SES, and DEP) may be used interchangeably for capturing the same dimension of neighborhood SES (or deprivation) in a given study area.

While the findings from three previous studies [27,28,29] may have collectively shed some light on the potential use of MHI or MFI in the context of data analytics and multilevel analyses, its application in research studies remains questionable not only for the study of neighborhood effects on health [1,2], but also for the evidence-based decision making in public health [30,31,32,33]. In particular, spatial–temporal variations in the size and internal structure of geographic ranges [34] have been known to influence the consistency of a measurement of interest. Without due consideration, an inappropriate use of MHI or MFI in research studies may jeopardize a meaningful synthesis of scientific evidence or scientific knowledge into health promotion interventions, urban and regional planning, and health policies. From a dissemination and implementation science standpoint, a detail-oriented approach to health research is likely to improve the quality of research synthesis [30,31,32,33] in the US since the geographic and population characteristics vary from state to state as well as across urban, suburban, and rural areas within each state. To better understand the usefulness of MHI and MFI [27] and its application [28,29] in research studies, therefore, the relationships between simple measures and composite measures of neighborhood SES (or deprivation) were examined in the State of California and its seven Metropolitan Statistical Areas (MSAs) at four time periods.

## 2. Materials and Methods

### 2.1. Study Design

The overarching objective of this study was to understand whether spatial–temporal variations in the size and internal structure of geographic ranges [34] have an influence on the measurement of neighborhood SES (or deprivation). This was accomplished by accounting for potential influences of geographic selection bias (i.e., spatial variations in the size and internal structure of geographic areas as well as the population size and population structure) and demographic changes over time (i.e., temporal variations in the population size and population structure) in assessing the consistency of measurement.

To account for potential influences of geographic selection bias, the State of California and its seven MSAs were considered as the study areas. MSAs are delineated by the US Office of Management and Budget and are configured to represent a core area containing a substantial population nucleus, together with adjacent communities having a high degree of economic and social integration with that core. Delineated by one or more contiguous counties and equivalent entities, each MSA consists at least one urbanized area with 50,000 or more inhabitants.

In conjunction with the geographic ranges of the study areas, the 2000 Census data as well as the 2005–2009, 2010–2014, and 2015–2019 American Community Survey (ACS) data were considered in this study to account for potential influences of demographic changes over time. The ACS is an ongoing national survey of the US population that provides demographic, housing, social, and economic information summarized at multiple levels of the census geographic entities (e.g., defined by block groups, census tracts, counties, and states). It is conducted every year since 2005 in replacement of the decennial census. On the whole, the five-year ACS estimates are based on a larger sample size, and thus more reliable, than the one- and three-year ACS estimates.

To put the context of this study in perspective, geographic and population characteristics of the study areas are summarized in Table 2. Here, the 2000 and 2010 cartographic boundary shapefiles were obtained from the US Census Bureau [35], and then the total land area (in square kilometers) was recalculated in ArcGIS 10.2 (ESRI Inc., Redlands, CA, USA). Since cartographic boundaries extend into rivers, ponds, lakes, and the Pacific Ocean, bodies of water were removed from these boundary shapefiles in this recalculation process (using the erase tool in ArcGIS) to better represent the actual land surface area of each study area. The shapefile of bodies of water was obtained from the Data & Maps Collection for ArcGIS on DVD, and all shapefiles were projected using the NAD 1983, State Plane Coordinate System. In Table 2, the total population was calculated by aggregating the census-tract-level estimates into each study area.

### 2.2. Neighborhood Measures

The 2000 Census data as well as the 2005–2009, 2010–2014, and 2015–2019 ACS data at the census tract level were obtained from the US Census Bureau’s website [36] and were used to create unidimensional measures of neighborhood SES (or deprivation) in each of the study areas. Since census-tract-level median household income (MHI) and median family income (MFI) estimates are readily available in these four data, these were simply used as two simple measures of neighborhood SES (or deprivation). On the other hand, following the conceptual and methodological approaches described in Table 1, five composite measures of neighborhood SES (or deprivation) were derived from the area-based indexes of SEA [20], SEP [21], SED [22], SES [23], and DEP [24] (for more details, refer to the original articles). Therefore, a composite measure refers to either a summary score derived from a sum of z-scores, a first factor score derived from factor analysis, or a first component score derived from principal component analysis. Using census tracts as the unit of analysis, five composite measures of neighborhood SES (or deprivation) were calculated separately for each study area and for each time period in R 4.1.3 [37]. When carrying out computational analyses, a sum of z-score was computed by a combination of base R functions, whereas factor analysis and principal component analysis were carried out by the fa and principal function, respectively, in the psych package [38].

Similar to previous studies conducted in the US [3,4,5,6,7,8,9,10,11,12,13,14,15,27,28,29], census tracts were used to denote neighborhoods for two main reasons: (i) some area-level socioeconomic indicators comprised in the construction of area-based indexes [20,21,22,23,24] were not available at the block group level (i.e., a subdivision of census tracts), and (ii) census tracts are a manifestation of national democratic governance informed by local input, and historically created in accordance with uniform standards [39]. Note that block groups have been used in some US studies, but block-group-level estimates are generally quite unreliable with relatively large margins of error than the census-tract-level estimates.

In this study, five area-based indexes developed in the US [20,21,22,23,24] were considered for comparing composite measures of neighborhood SES (or deprivation) that are comprised of different census-tract-level socioeconomic indicators and are based on different computational methods. The choice of five area-based indexes was also intended to build upon the previous study conducted in four US cities [27]. To avoid duplication, other common area-based indexes (which are typically modified, refined, or renamed versions of an older area-based index developed around the turn of this century) were excluded in this study. For example, the area-based indexes of area deprivation developed by Kind et al. [40] and SED developed by Singh [22] share the same conceptual and methodological approach [40], and the area-based indexes of SES developed by Yost et al. [41] and SEP developed by Krieger et al. [21] have been shown to yield very similar results in an empirical study [26]. Therefore, these area-based indexes [40,41], among others, were not considered in this study.

### 2.3. Statistical Analysis

For examining the relationships between two simple measures and five composite measures of neighborhood SES (or deprivation), separate correlation analyses were conducted for each study area and for each time period in R 4.1.3 [37]. The cor function in the base R stats package was used to carry out a series of correlation analyses, and the results are summarized in Table 3 (for the State of California) and Table 4, Table 5, Table 6, Table 7, Table 8, Table 9 and Table 10 (for the seven MSAs). In handling the missingness of census-tract-level socioeconomic indicators in the 2000 Census data as well as the 2005–2009, 2010–2014, and 2015–2019 ACS data, complete cases were only considered in the correlation analysis. As shown in Table 3, Table 4, Table 5, Table 6, Table 7, Table 8, Table 9 and Table 10, the Pearson’s correlation coefficient (*r*) was used to understand the relationships between seven measures of neighborhood SES (or deprivation) across the study areas and over the four time periods.

While the analytical approach of this study was descriptive in nature, correlation coefficients have been commonly used to assess the consistency of a measure [42,43]. The main reason for using correlation analysis lays in the fact that a value of correlation coefficient (which ranges between −1.00 to 1.00) is a dimensionless number (i.e., a pure number without any units) and is invariant to the different units of measurement (i.e., units-invariant) [44,45,46]. In other words, the strength of a linear correlation between two measures (the closer the value to −1.00 or 1.00, the stronger the linear correlation) may be affected by the amount of variability in a dataset, the different shapes of frequency distributions, the presence of an outlier or outliers, and/or the measurement error(s) [47], not by the units of measurement. This unit-invariant property was especially important in this study because composite measures of neighborhood SES (or deprivation) derived from the five area-based indexes [20,21,22,23,24] do not share the same unit of measurement. For these reasons, the results from a series of correlation analyses (Table 3, Table 4, Table 5, Table 6, Table 7, Table 8, Table 9 and Table 10) provides an intuitive comparison of the strengths and directions of linear correlations not only in each study area and at each time period, but also across the study areas and over the four time periods.

The same principles apply to further efforts on ensuring the measurement validity in different geographic areas. To set forth a foundation for such efforts, preliminary results from a series of correlation analyses for other 47 states and the District of Columbia (not considering any MSAs within each state) in the conterminous US (i.e., the contiguous US) are provided in Supplementary Materials (Tables S1–S48).

## 3. Results

Despite the differences in how five composite measures of neighborhood SES (or deprivation) were derived from selected area-based indexes (Table 1) and how population growth took place over the course of two decades (Table 2), simple measures of MHI and MFI showed consistent relationships with composite measures of SEA, SEP, SED, SES, and DEP (Table 3, Table 4, Table 5, Table 6, Table 7, Table 8, Table 9 and Table 10): both MHI and MFI were very strongly and positively correlated with each other (0.90 ≤ *r* ≤ 0.98); both MHI and MFI were strongly or very strongly and positively correlated with SEA (0.82 ≤ *r* ≤ 0.93); both MHI and MFI were strongly or very strongly, but negatively correlated with SEP (−0.84 ≤ *r* ≤ −0.94); both MHI and MFI were strongly or very strongly, but negatively correlated with SED (−0.84 ≤ *r* ≤ −0.96); both MHI and MFI were strongly or very strongly, but negatively correlated with SES (−0.81 ≤ *r* ≤ −0.95); both MHI and MFI were strongly, but negatively correlated with DEP (−0.72 ≤ *r* ≤ −0.89). Similar to the previous study conducted in four US cities [27], the relationships of MHI and MFI with DEP were slightly weaker than those with SEA, SEP, SED, and SES.

Within the consistent relationships shown in Table 3, Table 4, Table 5, Table 6, Table 7, Table 8, Table 9 and Table 10, a very minor, but noticeable inconsistency worth mentioning. On one hand, MHI and/or MFI were strongly, but negatively correlated with DEP (−0.72 ≤ *r* ≤ −0.79) in the State of California (Table 3) and five MSAs (Table 5, Table 6, Table 7, Table 8 and Table 9). On the other hand, the strengths of linear correlations were consistently stronger (−0.80 ≤ *r* ≤ −0.89) in the Los Angeles–Long Beach–Anaheim MSA (Table 4) and the Fresno MSA (Table 10). This is the main reason why the overall relationships of MHI and MFI with DEP were coupled with larger dispersions relative to those with SEA, SEP, SED, and SES. While a specific source of such minor inconsistency cannot be identified from this study, DEP may be more sensitive to the size and/or internal structure of a study area than SEA, SEP, SED, and SES. Otherwise, the strengths of linear correlations between two simple measures and other four composite measures fluctuated to a certain degree, but the degrees of fluctuations were negligible.

In addition to the consistent relationships between two types of measures, similar relationships were also evident among five composite measures across the study areas and over the four time periods (Table 3, Table 4, Table 5, Table 6, Table 7, Table 8, Table 9 and Table 10): SEA was very strongly, but negatively correlated with SEP, SED, SES, and DEP (−0.85 ≤ *r* ≤ −0.98); SEP was very strongly and positively correlated with SED, SES, and DEP (0.92 ≤ *r* ≤ 0.99); SED was very strongly and positively correlated with SEP, SES, and DEP (0.92 ≤ *r* ≤ 0.98); SES was very strongly and positively correlated with SEP, SED, and DEP (0.87 ≤ *r* ≤ 0.99); and DEP was very strongly and positively correlated with SEP, SED, and SES (0.87 ≤ *r* ≤ 0.98). Unlike the slightly weaker relationships with MHI and MFI, DEP were very strongly correlated, either negatively or positively, with SEA, SEP, SED, and SES with small dispersions.

Overall, spatial–temporal variations in the size and internal structure of geographic ranges [34] do not appear to have had an influence on the measurement of neighborhood SES (or deprivation) in the State of California and its seven MSAs (Table 3, Table 4, Table 5, Table 6, Table 7, Table 8, Table 9 and Table 10); the only exception was a subtle fluctuation of the strengths of linear correlations between MHI, MFI, and DEP. To a similar extent, these consistent relationships were also evident in other 44 contiguous states and the District of Columbia (Tables S1–S23, S25–S31, S33–S38 and S40–S47). Note that DEP showed slightly weaker relationships with two simple measures and/or four measures in some states. Unlike the rest of 44 contiguous states and the District of Columbia, the inconsistent relationships apparent in four states appertain to the strengths of linear correlations between seven measures that fluctuated at different time periods, where some of them showed much weaker relationships, in the State of Montana (Table S24), the State of North Dakota (Table S32), the State of South Dakota (Table S39), and the State of Wyoming (Table S48).

While more detailed examinations are needed in four states, the results shown in Table 3, Table 4, Table 5, Table 6, Table 7, Table 8, Table 9 and Table 10 as well as in Tables S1–S23, S25–S31, S33–S38 and S40–S47 validate the results from four US cities [27] by accounting for potential influences of geographic selection bias and demographic changes over time.

## 4. Discussion

Given the spatial and temporal considerations in study design (Table 2), the results of this study (Table 3, Table 4, Table 5, Table 6, Table 7, Table 8, Table 9 and Table 10) support an analytical thinking that simple measures of MHI and MFI may capture the same dimension of neighborhood SES (or deprivation) as the five composite measures of SEA, SEP, SED, SES, and DEP [27], and that either MHI or MFI may be used as a unidimensional measure of neighborhood SES (or deprivation) in a regression analysis [28,29]. In reference to the results from method comparison studies [25,26,28,29], a regression analysis using one of the strongly or very strongly correlated measures of neighborhood SES (or deprivation) would yield similar parameter estimates as the ones using its alternative measures (the stronger the linear correlation between the measures, the greater the similarity between the regression outputs). Therefore, time and labor devoted to developing a perfect area-based index and/or to dissecting out fairly small differences among comparable measures may not be productive research endeavors [48]. Taking these under consideration, a use of MHI or MFI in health research may be regarded as a simpler approach (or a time- and labor-saving approach) to the measurement of neighborhood SES (or deprivation) [27] in the State of California.

Notwithstanding the usefulness of MHI and MFI [27] validated in this study (Table 3, Table 4, Table 5, Table 6, Table 7, Table 8, Table 9 and Table 10), well-established conceptual and methodological approaches to the measurement of neighborhood SES (or deprivation), not limited to the ones [20,21,22,23,24] considered in this study, may overshadow the potential application of MHI or MFI [28,29] in research studies. This logical conflict stems from a longstanding premise that SES (including socioeconomic position and social class) has been conceived as a multifactorial construct of several socioeconomic domains (e.g., education, employment, income, occupation, and wealth) [49,50,51]. The same can be said for deprivation where it has been conceived as a multifaceted condition of people’s experiences in their daily lives (e.g., community engagement, housing conditions, recreational amenities, and unemployment) [49,50,51]. Grounded on the multicomponent conceptualization of SES and deprivation, both share a common basis to oppose against the use of single socioeconomic indicator for capturing a multifactorial or multifaceted phenomenon of neighborhood SES (or deprivation). Within the realm of measurement validity, however, conceptual or theoretical constructs cannot be measured directly and can only be inferred from observations of phenomena that are thought to represent the construct [42,43]. Since the complexity of a real-world situation evades even a well-rounded concept or theory, a comprehensive approach to the measurement of a construct or a phenomenon, in some situations, does not necessarily guarantee its superiority over a simpler approach or a reductionist approach [52,53,54]. By analogy, these principles provide a basic means for illuminating the similarities between two types of measures considered in this study (Table 3, Table 4, Table 5, Table 6, Table 7, Table 8, Table 9 and Table 10) and for recognizing such conceptually and methodologically different measures as comparable measures. From a measurement perspective [52,53,54], therefore, as long as strong or very strong linear correlations between two types of measures (at the very least, one of each) can be confirmed in a given study area [27], either MHI or MFI may be used as a unidimensional measure of neighborhood SES (or deprivation) in a regression analysis [28,29].

A main advantage of using MHI or MFI as a unidimensional measure of neighborhood SES (or deprivation) over its composite counterparts, not limited to the ones [20,21,22,23,24] considered in this study, rests on the fact that reducing the potential influences of an outlier or outliers [55,56,57,58] becomes a much easier task. In simplest forms, an outlier presents itself as an abnormal error in the computation of an unweighted or weighted summary score and/or as an extreme manifestation of the random variability in a dataset [59]. The former pertains to composite measures and the later to both types of measures. Since the computational methods for deriving composite measures (in a general sense) involve both forms of outliers, measurement error may mask certain patterns in a dataset [60]. This mostly attributes to the unforeseeable nature of a multivariate outlier (i.e., an outlier induced by combining a set of census-tract-level socioeconomic indicators) where it emerges even in an outlier-free dataset [61]. Additionally, detecting or identifying a multivariate outlier may not be a trivial task and may require a thorough understanding of the statistical and computational procedures in outlier analysis [62]. Because useful dimension reduction techniques (e.g., a sum of z-scores, factor analysis, and principal component analysis), to a certain extent, act like a “black box” operation [61], one or more “hidden” multivariate outliers may not only distort the shape of a frequency distribution [60], but may also alter the spatial distribution of a derived composite measure [55,56,57,58]. Unlike multivariate outliers, one or more univariate outliers (i.e., an outlier or outliers manifested in a census-tract-level socioeconomic indicator) may be sufficiently handled by implementing either the areal mean filer or the areal median filter [63]. Note that these two areal filtering approaches calculate a mean or a median by pooling information from the adjacent or surrounding enumeration units (e.g., census tracts), not from the adjacent or contiguous cells in a spreadsheet (e.g., Microsoft Excel). Bearing in mind the possible implementation of areal filtering approaches for smoothing univariate outliers (not multivariate outliers) [63], therefore, either MHI or MFI may provide an outlier-resistant approach to the measurement of neighborhood SES (or deprivation) in a given study area.

In addition to the conceptual and methodological differences in the measurement of neighborhood SES (or deprivation), a misconception about census-tract-level (or block-group-level) socioeconomic data may also play a role in overshadowing the potential application of MHI or MFI [28,29] in research studies. This conceptional conflict stems, in part, from empirical studies [64,65] that have provided analytical frameworks (or justifications) for using census-tract-level (or block-group-level) socioeconomic data as proxies for individual-level socioeconomic data. The underlying motivation for such a conceptualization was to overcome the absence of individual-level socioeconomic data in many patient registries (e.g., health services registries and disease or condition registries) that are routinely collected and widely used for scientific, clinical, and health policy purposes in the US. While these analytical frameworks [64,65] have been developed to address the obstacles imposed on some academic disciplines and professionals (or practitioners), quite a few empirical studies [66,67,68,69,70] have demonstrated the inappropriateness of treating census-tract-level (or block-group-level) socioeconomic data as if they were individual-level data. Dating back as early as the mid-1990s, these empirical studies [66,67,68,69,70] revealed low degrees of agreement (concordance) between two or more census-tract-level (or block-group-level) socioeconomic indicators of income (including poverty and wealth), education, and/or occupation and its individual-level counterparts, and also demonstrated such socioeconomic indicators to provide complementary information at each level in a regression analysis. Here, fairly large degrees of disagreement between census-tract-level (or block-group-level) and individual-level socioeconomic indicators, as well as their statistical independence in a regression analysis, were due to substantial heterogeneity of demographic characteristics within census tracts (or block groups) [66,67,68,69,70]. Similar to how different types of census-tract-level (or block-group-level) socioeconomic indicators have been determined inappropriate as proxies for individual-level socioeconomic indicators [66,67,68,69,70], census-tract- and block-group-level MHI [68] and block-group-level MFI [70] have been deemed noninterchangeable with individual-level income.

Building upon the findings of and suggestions from these empirical studies [66,67,68,69,70] as well as many other studies conducted in the US and elsewhere, census-tract-level socioeconomic data have been used as crude measures for quantifying compositional or contextual characteristics of neighborhoods that may shape the health of individuals residing in them [1,2] (see also Refs. [71,72] for informative reviews on this topic). In other words, small-area- and individual-level data have different meanings, and thus census-tract-level socioeconomic data have been merged with a health data as a means to compensate for uncollected or unobserved data on individual’s place of residence. Rooted in such conceptual and theoretical foundations of neighborhoods and health research [1,2], composite measures of neighborhood SES (or deprivation) used in a large body of previous studies [3,4,5,6,7,8,9,10,11,12,13,14,15] have been used as a proxy for material and social deprivation [49,50,51,73]. While direct measurement is not possible, a relative level of deprivation has been conceived to increase from highest to lowest SES (or least to most deprived) neighborhoods [49,50,51,73]. Among the various forms of deprivation [73], material deprivation refers to a deprivation of basic goods, infrastructure, and services related to people’s daily lives (e.g., adequate housing, owing a vehicle, having an active telephone line, and access to areas or facilities for recreational activities), and social deprivation refers to a deprivation of community resources related to people’s social ties with their societal members (e.g., mutual trust between each other, perception of fairness among one another, helpfulness towards others, and respect for social rules). Here, material deprivation [73] evokes the concept of poverty (including economic well-being and capabilities for human well-being) [49,50,51] and social deprivation [73] educes the concept of social cohesion (including social capital and social network) [49,50,51]. Taken together, in much the same way as previous studies [3,4,5,6,7,8,9,10,11,12,13,14,15] have been using composite measures of neighborhood SES (or deprivation) to inquire into the neighborhood effects on health [1,2,71,72], MHI or MFI may also be used as a proxy for material and social deprivation [49,50,51,73] by which its relative level coincides with the level of neighborhood SES (or deprivation) in a given study area.

Above-mentioned analytical and conceptual reasoning illuminate additional values to the potential application of MHI or MFI [28,29] in research studies. To promote a meaningful synthesis of scientific evidence or scientific knowledge [30,31,32,33], however, one or more scaling techniques need to be applied for ensuring a meaningful comparison of research findings with previously reported findings [3,4,5,6,7,8,9,10,11,12,13,14,15] based on multilevel analyses. By definition, an increase in MHI and MFI corresponds to a change from lowest to highest SES (or most to least deprived) neighborhoods. Comparable to the composite measure of SEA [20], MHI and MFI may be used to examine a protective effect on health. If research studies aim to examine an adverse effect on health, then MHI and MFI need to be multiplied or divided by −1 (denoted as MHI* and MFI*, respectively) to reverse (or flip) the direction of their integers [27]. Comparable to the composite measures of SEP [21], SED [22], SES [23], and DEP [24], an increase in MHI* and MFI* corresponds to a change from highest to lowest SES (or least to most deprived) neighborhoods. Note that this scaling of MHI and MFI only changes the sign of integers and does not affect the width and height of their frequency distributions.

Prior to incorporating MHI or MFI, or alternatively MHI* or MFI*, as a continuous covariate (x) in a multilevel analysis, standardization (for a continuous or binary outcome of interest) and normalization (for a binary outcome of interest) provide an easier comparison of regression coefficients between and across different research studies [29]. Since MHI and MFI, and thus MHI* and MFI*, generally follow a slightly skewed distribution, a common process of standardization is to subtract its median ($\tilde{x}$) and then divide by its interquartile range (IQR): (x − $\tilde{x}$)/IQR. Or, another common process of standardization is to simply divide by its IQR: x/IQR. Note that the IQR is defined as the distance between the 25th and 75th percentiles of a frequency distribution, and median-centering [74] is particularly important to bring two or more continuous covariates into proportion with one another, and when an interaction effect between neighborhood-level covariates or between neighborhood- and individual-level covariates were to be examined. For improving the interpretability of a regression coefficient not only in terms of a typical deviation from the center (i.e., the median), but also a deviation between both ends of the spectrum, a common process of normalization is to modify its original range into a range between 0 and 1: (x − x_min_)/(x_max_ − x_min_) [75]. While normalization is not applicable or relevant for multilevel linear regression models, interpreting a standardized and normalized measures of MHI or MFI, or alternatively MHI* or MFI*, from multilevel logistic regression models lead to a more rounded view of the protective or adverse effects of neighborhood SES (or deprivation) on health within a certain geographic area as well as across different geographic areas [29].

Conforming to how composite measures of neighborhood SES (or deprivation), not limited to the ones [20,21,22,23,24] considered in this study, have often been analyzed in previous studies [3,4,5,6,7,8,9,10,11,12,13,14,15], MHI or MFI, or alternatively MHI* or MFI*, may be converted into multiple categories (or groups) of equal size for an easier comparison between one or more categories (or groups) and its reference category (or group). A common process of categorization is to split into two, three, four, and five categories (or groups), respectively, by the median (2-quantiles), tertiles (3-quantiles), quartiles (4-quantiles), and quintiles (5-quantiles) of a frequency distribution [76]. However, splitting a continuous covariate into two categories (or groups) has long been known to come at a cost of losing statistical power (or efficiency) and residual confounding [77,78,79], and increasing the number of cutoff points do not satisfy basic assumptions about within-category (or within-group) homogeneity and between-category (or between-group) equivalence [80,81,82]. Put differently, a rather arbitrary and data-driven process of categorization leads to an accentuation of differences within and similarities between respective levels of neighborhood SES (or deprivation), which undermines the statistical validity of a multilevel analysis. Unless incorporating MHI or MFI, or alternatively MHI* or MFI*, as a continuous covariate violates the underlying assumptions of regression analysis [83,84,85], excluding the common misconception about normally distributed response and/or covariates [84], therefore, a categorization of MHI or MFI, or alternatively MHI* or MFI*, is not recommended [27].

Instead of investigating into an optimal number and location of cutoff points (or a threshold effect) of MHI or MFI, or alternatively MHI* or MFI*, more fruitful efforts are to explore a use of generalized geoadditive mixed models (GGAMMs) [86] for uncovering their nonlinear effects. Note that GGAMMs are a spatial-multilevel version of generalized additive models (GAMs) [87,88,89] and a spatial version of generalized additive mixed models (GAMMs) [90]. The geo (i.e., spatial) component in GGAMMs is particularly important to take into account for spatial autocorrelation in the geographic (or geospatial) aspect of a dataset (e.g., census-tract-level socioeconomic indicators from the decennial census and the ACS). Note that spatial autocorrelation refers to the dependencies among observations resulting from a clustering of similar characteristics (positive spatial autocorrelation) or dissimilar characteristics (negative spatial autocorrelation) in geographic space [91]. The presence of spatial autocorrelation in a regression analysis violates one of the key assumptions that residual errors are independent and identically distributed, and thus calls into question the statistical validity of hypothesis testing [92,93]. While multilevel regression (or hierarchical) models [16,17,18,19] commonly used in previous studies [3,4,5,6,7,8,9,10,11,12,13,14,15] are capable of accounting for within-neighborhood (i.e., within-census-tract) dependencies, these models are incapable of accounting for between-neighborhood (i.e., between-census-tract) dependencies. Therefore, GGAMMs [86] are likely to provide statistical accuracy and precision than the multilevel (or hierarchical) regression models [16,17,18,19] by removing spatial dependencies inherent to area-based measures of neighborhood characteristics, such as neighborhood SES (or deprivation) and population density. Given the computational intricacy of GGAMMs [86], however, its applications in neighborhoods and health research [1,2] are only recommended for researchers who have an extensive knowledge of modeling complex spatial and hierarchical data structures. Hence, a use of GGAMMs [86] must be explored with much caution.

A sequence of practical arguments discussed thus far collectively enlightens the usefulness of MHI and MFI [27] and its application [28,29] in research studies within the context of data analytics and multilevel analyses. From a statistical point of view, the results of this study (Table 3, Table 4, Table 5, Table 6, Table 7, Table 8, Table 9 and Table 10) are likely to be generalizable to smaller or larger geographic ranges of human settlements within the State of California (i.e., counties with a total population of less than one million or contiguous counties with a total population of greater than 18.7 million, but less than 39.3 million). However, further efforts are needed to validate the strong linear correlation(s) of MHI and/or MFI, or alternatively MHI* and/or MFI*, with one or more composite measures of neighborhoods SES (or deprivation) in different spatial and/or temporal settings. In doing so, a choice of area-based index(es) needs not be exclusive to the ones [20,21,22,23,24] considered in this study, but to be inclusive of those used in previous studies [3,4,5,6,7,8,9,10,11,12,13,14,15] or in empirical studies, which may not be included in existing review articles. Moreover, similar efforts are also needed in other 47 contiguous states and the District of Columbia (Tables S1–S48), but call for assessing a wide array of spatial-temporal variations in the size and internal structure of geographic ranges [34] in each state (e.g., multiple counties, contiguous counties, and MSAs). Since MHI or MFI has already been used in some previous studies [94,95,96,97,98,99], more detailed examinations on the validity of MHI or MFI, or alternatively MHI* or MFI*, in different geographic areas are likely to foster a comprehensive synthesis of scientific evidence or scientific knowledge [30,31,32,33] for a wide variety of academic disciplines and professionals (or practitioners) across the conterminous US.

Outside the US, area-based indexes have also been developed in industrialized countries, such as in Canada [100,101,102], Denmark [103,104], France [105,106], Spain [107,108], Sweden [109,110], and the United Kingdom [111,112,113] to name a few. While composite measures of neighborhood SES (or deprivation) derived from such area-based indexes have been used in research studies, the usefulness of MHI and MFI [27] and its application [28,29] as well as the results of this study (Table 3, Table 4, Table 5, Table 6, Table 7, Table 8, Table 9 and Table 10) and a sequence of practical arguments discussed above, may be inapplicable or irrelevant to those countries. This is because not all industrialized countries routinely collect information on income (or wealth) in their respective population censuses or make such information easily accessible for scientific, clinical, and health policy purposes. Hence, a conceptualization of MHI and MFI along with their reversed form, MHI* and MFI*, as a unidimensional measures of neighborhood SES (or deprivation) and its application in research studies may only be applicable or relevant to non-US countries with small-area-level income estimates equivalent to the census-tract-level MHI or MFI estimates from the US Census Bureau.

## 5. Conclusions

The results of this study (Table 3, Table 4, Table 5, Table 6, Table 7, Table 8, Table 9 and Table 10) suggest that MHI and MFI may be considered comparable to the composite measure of SEA [20] for capturing a change from lowest to highest SES (or most to least deprived) neighborhoods, and that, by multiplying or dividing MHI and MFI by −1, MHI* and MFI* may be considered comparable to the composite measures of SEP [21], SED [22], SES [23], and DEP [24] for capturing a change from highest to lowest SES (or least to most deprived) neighborhoods. In reference to the method comparison studies [28,29], either MHI or MFI, or alternatively MHI* or MFI*, may be used as a unidimensional measure of neighborhood SES (or deprivation) to inquire into the neighborhood effects on health [1,2,71,72] in the State of California. To avoid collinearity or multicollinearity in a multilevel analysis, however, either MHI or MFI may be used for examining a protective effect on health, and either MHI* or MFI* may be used for examining an adverse effect on health.

Besides the practical arguments discussed above, a conceptualization of MHI and MFI along with their reversed form, MHI* and MFI*, as a unidimensional measure of neighborhood SES (or deprivation) come with a set of practical benefits:

1. little time for preparation (i.e., readily available from the US Census Bureau’s website),
2. less effort on exploratory data analysis (e.g., calculating summary statistics and displaying a boxplot or a histogram in Microsoft Excel) and map visualization (e.g., using a Map chart in Microsoft Excel),
3. very few missing estimates within a given study area (e.g., defined by a city boundary, a county boundary, or a combination of contiguous county boundaries),
4. reasonable standard of precision (with a margin of error at the 90% confidence level) across different geographic areas,
5. consistent interpretation and straightforward comparison of research findings for research synthesis, and
6. effective dissemination and mutual understanding of scientific evidence or scientific knowledge across academic disciplines and professional fields.

While empirical validation studies are needed in the future, a use of MHI or MFI, or alternatively MHI* or MFI*, in health research may be regarded as a simpler approach (or a time- and labor-saving approach) to the measurement of neighborhood SES (or deprivation) in a wide range of geographic areas.

## Figures and Tables

**Table 1:** Description of five area-based indexes developed in the United States.

Area-Based Indexes	Census-Tract-Level Socioeconomic Indicators	Computational Method
Socioeconomic Advantage (SEA) [20]	Log of median household income (US $), log of median housing value (US $), receiving interest, dividend, or net rental income (%), completed high school education (%), completed college education (%), and executive, managerial, or professional occupations (%).	Sum of Z-scores
Socioeconomic Position (SEP) [21]	Working class (%), unemployed (%), below poverty (%), less than high school degree (%), expensive homes (%), and median household income (US $).	Sum of Z-scores
Socioeconomic Deprivation (SED) [22]	Less than 9 the grade education (%), more than high school education (%), white-collar occupations (%), median family income (US $), income disparity, median home value (US $), median gross rent ($), median monthly mortgage ($), owner-occupied housing units (%), unemployed (%), below poverty (%), below 150% of the poverty threshold (%), single-parent households with dependents (%), without a motor vehicle (%), without a telephone (%), without complete plumbing (%), and log of households with more than one person per room (%).	Factor Analysis
Socioeconomic Status (SES) [23]	High school graduates (%), median family income (US $), median housing value ($), blue-collar occupations (%), and unemployed (%).	Principal Component Analysis
Deprivation (DEP) [24]	Males in management and professional occupations (%), households with more than one person per room (%), below poverty (%), female-headed households with dependents (%), with public assistance income (%), household income less than $30,000 (%), less than high school education (%), and unemployed (%).	Factor Analysis

**Table 2:** Geographic and population characteristics of the study areas.

	Delineated Counties (#)	Census Tracts (#)		Total Land Area (km^2^) ^a^
		2000	2010	
State of California	58	7049	8037	402,887
Los Angeles–Long Beach–Anaheim MSA	2	2631	2924	12,572
San Francisco–Oakland–Berkeley MSA	5	871	976	6396
Riverside–San Bernardino–Ontario MSA	2	587	822	70,476
San Diego–Chula Vista–Carlsbad MSA	1	605	627	10,897
Sacramento–Roseville–Folsom MSA	4	403	484	13,191
San Jose–Sunnyvale–Santa Clara MSA	2	349	383	6930
Fresno MSA	1	158	199	15,447
	**Total Population (#) ^b^**			
	**2000**	**2005–2009**	**2010–2014**	**2015–2019**
State of California	33,871,648	36,308,527	38,061,951	39,278,430
Los Angeles–Long Beach–Anaheim MSA	12,365,627	12,762,126	13,055,565	13,244,547
San Francisco–Oakland–Berkeley MSA	4,123,740	4,218,534	4,466,251	4,701,332
Riverside-San Bernardino-Ontario MSA	3,254,821	4,022,939	4,345,485	4,560,470
San Diego–Chula Vista–Carlsbad MSA	2,813,833	2,987,543	3,183,143	3,316,073
Sacramento–Roseville–Folsom MSA	1,796,857	2,076,579	2,197,422	2,315,980
San Jose–Sunnyvale–Santa Clara MSA	1,735,819	1,784,130	1,898,457	1,987,846
Fresno MSA	799,407	890,750	948,844	984,521

^a^ Calculated using ArcGIS 10.2 by the author. ^b^ Summation of census-tract-level estimates. Abbreviation: MSA, Metropolitan Statistical Area.

**Table 3:** Relationships between simple and composite measures of neighborhood socioeconomic status in the State of California.

**2000 ^a^**	**MHI**	**MFI**	**SEA**	**SEP**	**SED**	**SES**	**DEP**
MHI	1.00	0.96	0.87	−0.88	−0.87	−0.87	−0.76
MFI	0.96	1.00	0.91	−0.91	−0.89	−0.92	−0.79
SEA	0.87	0.91	1.00	−0.96	−0.95	−0.98	−0.90
SEP	−0.88	−0.91	−0.96	1.00	0.97	0.98	0.93
SED	−0.87	−0.89	−0.95	0.97	1.00	0.94	0.97
SES	−0.87	−0.92	−0.98	0.98	0.94	1.00	0.90
DEP	−0.76	−0.79	−0.90	0.93	0.97	0.90	1.00
**2005–2009 ^b^**	**MHI**	**MFI**	**SEA**	**SEP**	**SED**	**SES**	**DEP**
MHI	1.00	0.94	0.87	−0.88	−0.88	−0.86	−0.78
MFI	0.94	1.00	0.90	−0.90	−0.89	−0.91	−0.79
SEA	0.87	0.90	1.00	−0.96	−0.93	−0.97	−0.89
SEP	−0.88	−0.90	−0.96	1.00	0.96	0.98	0.93
SED	−0.88	−0.89	−0.93	0.96	1.00	0.94	0.96
SES	−0.86	−0.91	−0.97	0.98	0.94	1.00	0.89
DEP	−0.78	−0.79	−0.89	0.93	0.96	0.89	1.00
**2010–2014 ^c^**	**MHI**	**MFI**	**SEA**	**SEP**	**SED**	**SES**	**DEP**
MHI	1.00	0.95	0.87	−0.89	−0.88	−0.86	−0.77
MFI	0.95	1.00	0.91	−0.91	−0.90	−0.91	−0.80
SEA	0.87	0.91	1.00	−0.96	−0.94	−0.97	−0.89
SEP	−0.89	−0.91	−0.96	1.00	0.96	0.98	0.93
SED	−0.88	−0.90	−0.94	0.96	1.00	0.94	0.96
SES	−0.86	−0.91	−0.97	0.98	0.94	1.00	0.89
DEP	−0.77	−0.80	−0.89	0.93	0.96	0.89	1.00
**2015–2019 ^d^**	**MHI**	**MFI**	**SEA**	**SEP**	**SED**	**SES**	**DEP**
MHI	1.00	0.95	0.89	−0.89	−0.89	−0.87	−0.77
MFI	0.95	1.00	0.92	−0.91	−0.92	−0.93	−0.80
SEA	0.89	0.92	1.00	−0.95	−0.93	−0.97	−0.87
SEP	−0.89	−0.91	−0.95	1.00	0.96	0.98	0.92
SED	−0.89	−0.92	−0.93	0.96	1.00	0.94	0.96
SES	−0.87	−0.93	−0.97	0.98	0.94	1.00	0.88
DEP	−0.77	−0.80	−0.87	0.92	0.96	0.88	1.00

^a^ Correlation matrix based on 6970 census tracts; 79 out of 7049 census tracts (1.12%) were omitted due to missing data. ^b^ Correlation matrix based on 6922 census tracts; 127 out of 7049 census tracts (1.80%) were omitted due to missing data. ^c^ Correlation matrix based on 7863 census tracts; 194 out of 8057 census tracts (2.41%) were omitted due to missing data. ^d^ Correlation matrix based on 7807 census tracts; 250 out of 8057 census tracts (3.10%) were omitted due to missing data. Abbreviations: MHI, Median Household Income; MFI, Median Family Income; SEA, Socioeconomic Advantage [20]; SEP, Socioeconomic Position [21]; SED, Socioeconomic Deprivation [22]; SES, Socioeconomic Status [23]; DEP, Deprivation [24].

**Table 4:** Relationships between simple and composite measures of neighborhood socioeconomic status in the Los Angeles–Long Beach–Anaheim Metropolitan Statistical Area.

**2000 ^a^**	**MHI**	**MFI**	**SEA**	**SEP**	**SED**	**SES**	**DEP**
MHI	1.00	0.96	0.87	−0.90	−0.87	−0.88	−0.80
MFI	0.96	1.00	0.91	−0.93	−0.90	−0.93	−0.82
SEA	0.87	0.91	1.00	−0.97	−0.96	−0.98	−0.93
SEP	−0.90	−0.93	−0.97	1.00	0.97	0.98	0.94
SED	−0.87	−0.90	−0.96	0.97	1.00	0.95	0.98
SES	−0.88	−0.93	−0.98	0.98	0.95	1.00	0.91
DEP	−0.80	−0.82	−0.93	0.94	0.98	0.91	1.00
**2005–2009 ^b^**	**MHI**	**MFI**	**SEA**	**SEP**	**SED**	**SES**	**DEP**
MHI	1.00	0.94	0.87	−0.88	−0.88	−0.86	−0.80
MFI	0.94	1.00	0.90	−0.90	−0.89	−0.91	−0.81
SEA	0.87	0.90	1.00	−0.96	−0.94	−0.97	−0.91
SEP	−0.88	−0.90	−0.96	1.00	0.96	0.98	0.94
SED	−0.88	−0.89	−0.94	0.96	1.00	0.94	0.97
SES	−0.86	−0.91	−0.97	0.98	0.94	1.00	0.91
DEP	−0.80	−0.81	−0.91	0.94	0.97	0.91	1.00
**2010–2014 ^c^**	**MHI**	**MFI**	**SEA**	**SEP**	**SED**	**SES**	**DEP**
MHI	1.00	0.94	0.87	−0.89	−0.88	−0.85	−0.80
MFI	0.94	1.00	0.90	−0.91	−0.90	−0.91	−0.82
SEA	0.87	0.90	1.00	−0.96	−0.95	−0.97	−0.91
SEP	−0.89	−0.91	−0.96	1.00	0.97	0.98	0.94
SED	−0.88	−0.90	−0.95	0.97	1.00	0.95	0.97
SES	−0.85	−0.91	−0.97	0.98	0.95	1.00	0.92
DEP	−0.80	−0.82	−0.91	0.94	0.97	0.92	1.00
**2015–2019 ^d^**	**MHI**	**MFI**	**SEA**	**SEP**	**SED**	**SES**	**DEP**
MHI	1.00	0.94	0.88	−0.89	−0.89	−0.86	−0.79
MFI	0.94	1.00	0.92	−0.91	−0.92	−0.92	−0.82
SEA	0.88	0.92	1.00	−0.95	−0.94	−0.97	−0.89
SEP	−0.89	−0.91	−0.95	1.00	0.96	0.97	0.94
SED	−0.89	−0.92	−0.94	0.96	1.00	0.94	0.96
SES	−0.86	−0.92	−0.97	0.97	0.94	1.00	0.91
DEP	−0.79	−0.82	−0.89	0.94	0.96	0.91	1.00

^a^ Correlation matrix based on 2600 census tracts; 31 out of 2631 census tracts (1.18%) were omitted due to missing data. ^b^ Correlation matrix based on 2573 census tracts; 58 out of 2631 census tracts (2.20%) were omitted due to missing data. ^c^ Correlation matrix based on 2836 census tracts; 93 out of 2929 census tracts (3.18%) were omitted due to missing data. ^d^ Correlation matrix based on 2803 census tracts; 126 out of 2929 census tracts (4.30%) were omitted due to missing data. Abbreviations: MHI, Median Household Income; MFI, Median Family Income; SEA, Socioeconomic Advantage [20]; SEP, Socioeconomic Position [21]; SED, Socioeconomic Deprivation [22]; SES, Socioeconomic Status [23]; DEP, Deprivation [24].

**Table 5:** Relationships between simple and composite measures of neighborhood socioeconomic status in the San Francisco–Oakland–Berkeley Metropolitan Statistical Area.

**2000 ^a^**	**MHI**	**MFI**	**SEA**	**SEP**	**SED**	**SES**	**DEP**
MHI	1.00	0.95	0.85	−0.86	−0.86	−0.82	−0.76
MFI	0.95	1.00	0.89	−0.90	−0.88	−0.89	−0.78
SEA	0.85	0.89	1.00	−0.96	−0.91	−0.97	−0.89
SEP	−0.86	−0.90	−0.96	1.00	0.95	0.98	0.94
SED	−0.86	−0.88	−0.91	0.95	1.00	0.92	0.95
SES	−0.82	−0.89	−0.97	0.98	0.92	1.00	0.90
DEP	−0.76	−0.78	−0.89	0.94	0.95	0.90	1.00
**2005–2009 ^b^**	**MHI**	**MFI**	**SEA**	**SEP**	**SED**	**SES**	**DEP**
MHI	1.00	0.92	0.87	−0.87	−0.88	−0.83	−0.78
MFI	0.92	1.00	0.88	−0.88	−0.89	−0.89	−0.80
SEA	0.87	0.88	1.00	−0.96	−0.92	−0.97	−0.89
SEP	−0.87	−0.88	−0.96	1.00	0.96	0.98	0.95
SED	−0.88	−0.89	−0.92	0.96	1.00	0.93	0.95
SES	−0.83	−0.89	−0.97	0.98	0.93	1.00	0.91
DEP	−0.78	−0.80	−0.89	0.95	0.95	0.91	1.00
**2010–2014 ^c^**	**MHI**	**MFI**	**SEA**	**SEP**	**SED**	**SES**	**DEP**
MHI	1.00	0.93	0.87	−0.87	−0.87	−0.83	−0.78
MFI	0.93	1.00	0.90	−0.90	−0.89	−0.90	−0.81
SEA	0.87	0.90	1.00	−0.96	−0.92	−0.97	−0.89
SEP	−0.87	−0.90	−0.96	1.00	0.95	0.98	0.95
SED	−0.87	−0.89	−0.92	0.95	1.00	0.92	0.96
SES	−0.83	−0.90	−0.97	0.98	0.92	1.00	0.91
DEP	−0.78	−0.81	−0.89	0.95	0.96	0.91	1.00
**2015–2019 ^d^**	**MHI**	**MFI**	**SEA**	**SEP**	**SED**	**SES**	**DEP**
MHI	1.00	0.93	0.89	−0.89	−0.89	−0.85	−0.79
MFI	0.93	1.00	0.92	−0.91	−0.92	−0.93	−0.83
SEA	0.89	0.92	1.00	−0.95	−0.90	−0.96	−0.87
SEP	−0.89	−0.91	−0.95	1.00	0.94	0.97	0.93
SED	−0.89	−0.92	−0.90	0.94	1.00	0.91	0.95
SES	−0.85	−0.93	−0.96	0.97	0.91	1.00	0.90
DEP	−0.79	−0.83	−0.87	0.93	0.95	0.90	1.00

^a^ Correlation matrix based on 861 census tracts; 10 out of 871 census tracts (1.15%) were omitted due to missing data. ^b^ Correlation matrix based on 854 census tracts; 17 out of 871 census tracts (1.95%) were omitted due to missing data. ^c^ Correlation matrix based on 956 census tracts; 24 out of 980 census tracts (2.45%) were omitted due to missing data. ^d^ Correlation matrix based on 950 census tracts; 30 out of 980 census tracts (3.06%) were omitted due to missing data. Abbreviations: MHI, Median Household Income; MFI, Median Family Income; SEA, Socioeconomic Advantage [20]; SEP, Socioeconomic Position [21]; SED, Socioeconomic Deprivation [22]; SES, Socioeconomic Status [23]; DEP, Deprivation [24].

**Table 6:** Relationships between simple and composite measures of neighborhood socioeconomic status in the Riverside–San Bernardino–Ontario Metropolitan Statistical Area.

**2000 ^a^**	**MHI**	**MFI**	**SEA**	**SEP**	**SED**	**SES**	**DEP**
MHI	1.00	0.97	0.84	−0.84	−0.89	−0.87	−0.77
MFI	0.97	1.00	0.90	−0.90	−0.94	−0.93	−0.84
SEA	0.84	0.90	1.00	−0.95	−0.94	−0.96	−0.90
SEP	−0.84	−0.90	−0.95	1.00	0.96	0.98	0.94
SED	−0.89	−0.94	−0.94	0.96	1.00	0.95	0.96
SES	−0.87	−0.93	−0.96	0.98	0.95	1.00	0.92
DEP	−0.77	−0.84	−0.90	0.94	0.96	0.92	1.00
**2005–2009 ^b^**	**MHI**	**MFI**	**SEA**	**SEP**	**SED**	**SES**	**DEP**
MHI	1.00	0.95	0.84	−0.85	−0.90	−0.86	−0.77
MFI	0.95	1.00	0.89	−0.90	−0.94	−0.92	−0.83
SEA	0.84	0.89	1.00	−0.93	−0.92	−0.96	−0.89
SEP	−0.85	−0.90	−0.93	1.00	0.94	0.98	0.93
SED	−0.90	−0.94	−0.92	0.94	1.00	0.94	0.95
SES	−0.86	−0.92	−0.96	0.98	0.94	1.00	0.91
DEP	−0.77	−0.83	−0.89	0.93	0.95	0.91	1.00
**2010–2014 ^c^**	**MHI**	**MFI**	**SEA**	**SEP**	**SED**	**SES**	**DEP**
MHI	1.00	0.95	0.82	−0.84	−0.89	−0.84	−0.75
MFI	0.95	1.00	0.87	−0.89	−0.93	−0.90	−0.81
SEA	0.82	0.87	1.00	−0.94	−0.92	−0.95	−0.88
SEP	−0.84	−0.89	−0.94	1.00	0.95	0.98	0.93
SED	−0.89	−0.93	−0.92	0.95	1.00	0.94	0.95
SES	−0.84	−0.90	−0.95	0.98	0.94	1.00	0.90
DEP	−0.75	−0.81	−0.88	0.93	0.95	0.90	1.00
**2015–2019 ^d^**	**MHI**	**MFI**	**SEA**	**SEP**	**SED**	**SES**	**DEP**
MHI	1.00	0.94	0.84	−0.84	−0.91	−0.85	−0.76
MFI	0.94	1.00	0.90	−0.88	−0.94	−0.92	−0.82
SEA	0.84	0.90	1.00	−0.93	−0.92	−0.95	−0.87
SEP	−0.84	−0.88	−0.93	1.00	0.94	0.97	0.93
SED	−0.91	−0.94	−0.92	0.94	1.00	0.95	0.94
SES	−0.85	−0.92	−0.95	0.97	0.95	1.00	0.90
DEP	−0.76	−0.82	−0.87	0.93	0.94	0.90	1.00

^a^ Correlation matrix based on 581 census tracts; 6 out of 587 census tracts (1.02%) were omitted due to missing data. ^b^ Correlation matrix based on 580 census tracts; 7 out of 587 census tracts (1.19%) were omitted due to missing data. ^c^ Correlation matrix based on 815 census tracts; 7 out of 822 census tracts (0.85%) were omitted due to missing data. ^d^ Correlation matrix based on 814 census tracts; 8 out of 822 census tracts (0.97%) were omitted due to missing data. Abbreviations: MHI, Median Household Income; MFI, Median Family Income; SEA, Socioeconomic Advantage [20]; SEP, Socioeconomic Position [21]; SED, Socioeconomic Deprivation [22]; SES, Socioeconomic Status [23]; DEP, Deprivation [24].

**Table 7:** Relationships between simple and composite measures of neighborhood socioeconomic status in the San Diego–Chula Vista–Carlsbad Metropolitan Statistical Area.

**2000 ^a^**	**MHI**	**MFI**	**SEA**	**SEP**	**SED**	**SES**	**DEP**
MHI	1.00	0.96	0.86	−0.86	−0.84	−0.86	−0.72
MFI	0.96	1.00	0.91	−0.90	−0.87	−0.91	−0.76
SEA	0.86	0.91	1.00	−0.95	−0.94	−0.97	−0.89
SEP	−0.86	−0.90	−0.95	1.00	0.96	0.98	0.92
SED	−0.84	−0.87	−0.94	0.96	1.00	0.94	0.96
SES	−0.86	−0.91	−0.97	0.98	0.94	1.00	0.89
DEP	−0.72	−0.76	−0.89	0.92	0.96	0.89	1.00
**2005–2009 ^b^**	**MHI**	**MFI**	**SEA**	**SEP**	**SED**	**SES**	**DEP**
MHI	1.00	0.93	0.87	−0.86	−0.86	−0.84	−0.74
MFI	0.93	1.00	0.90	−0.89	−0.89	−0.91	−0.78
SEA	0.87	0.90	1.00	−0.94	−0.93	−0.96	−0.87
SEP	−0.86	−0.89	−0.94	1.00	0.96	0.98	0.92
SED	−0.86	−0.89	−0.93	0.96	1.00	0.93	0.96
SES	−0.84	−0.91	−0.96	0.98	0.93	1.00	0.89
DEP	−0.74	−0.78	−0.87	0.92	0.96	0.89	1.00
**2010–2014 ^c^**	**MHI**	**MFI**	**SEA**	**SEP**	**SED**	**SES**	**DEP**
MHI	1.00	0.94	0.88	−0.87	−0.88	−0.84	−0.75
MFI	0.94	1.00	0.92	−0.92	−0.92	−0.92	−0.80
SEA	0.88	0.92	1.00	−0.95	−0.93	−0.96	−0.87
SEP	−0.87	−0.92	−0.95	1.00	0.96	0.98	0.92
SED	−0.88	−0.92	−0.93	0.96	1.00	0.94	0.96
SES	−0.84	−0.92	−0.96	0.98	0.94	1.00	0.89
DEP	−0.75	−0.80	−0.87	0.92	0.96	0.89	1.00
**2015–2019 ^d^**	**MHI**	**MFI**	**SEA**	**SEP**	**SED**	**SES**	**DEP**
MHI	1.00	0.95	0.89	−0.86	−0.90	−0.84	−0.79
MFI	0.95	1.00	0.92	−0.88	−0.92	−0.90	−0.81
SEA	0.89	0.92	1.00	−0.94	−0.93	−0.96	−0.88
SEP	−0.86	−0.88	−0.94	1.00	0.95	0.97	0.93
SED	−0.90	−0.92	−0.93	0.95	1.00	0.93	0.96
SES	−0.84	−0.90	−0.96	0.97	0.93	1.00	0.90
DEP	−0.79	−0.81	−0.88	0.93	0.96	0.90	1.00

^a^ Correlation matrix based on 597 census tracts; 8 out of 605 census tracts (1.32%) were omitted due to missing data. ^b^ Correlation matrix based on 594 census tracts; 11 out of 605 census tracts (1.82%) were omitted due to missing data. ^c^ Correlation matrix based on 616 census tracts; 12 out of 628 census tracts (1.91%) were omitted due to missing data. ^d^ Correlation matrix based on 610 census tracts; 18 out of 628 census tracts (2.87%) were omitted due to missing data. Abbreviations: MHI, Median Household Income; MFI, Median Family Income; SEA, Socioeconomic Advantage [20]; SEP, Socioeconomic Position [21]; SED, Socioeconomic Deprivation [22]; SES, Socioeconomic Status [23]; DEP, Deprivation [24].

**Table 8:** Relationships between simple and composite measures of neighborhood socioeconomic status in the Sacramento–Roseville–Folsom Metropolitan Statistical Area.

**2000 ^a^**	**MHI**	**MFI**	**SEA**	**SEP**	**SED**	**SES**	**DEP**
MHI	1.00	0.92	0.85	−0.85	−0.89	−0.82	−0.79
MFI	0.92	1.00	0.92	−0.88	−0.90	−0.92	−0.80
SEA	0.85	0.92	1.00	−0.92	−0.92	−0.97	−0.87
SEP	−0.85	−0.88	−0.92	1.00	0.94	0.96	0.94
SED	−0.89	−0.90	−0.92	0.94	1.00	0.92	0.95
SES	−0.82	−0.92	−0.97	0.96	0.92	1.00	0.88
DEP	−0.79	−0.80	−0.87	0.94	0.95	0.88	1.00
**2005–2009 ^b^**	**MHI**	**MFI**	**SEA**	**SEP**	**SED**	**SES**	**DEP**
MHI	1.00	0.91	0.83	−0.86	−0.88	−0.81	−0.75
MFI	0.91	1.00	0.88	−0.87	−0.89	−0.89	−0.78
SEA	0.83	0.88	1.00	−0.93	−0.90	−0.95	−0.86
SEP	−0.86	−0.87	−0.93	1.00	0.95	0.97	0.93
SED	−0.88	−0.89	−0.90	0.95	1.00	0.92	0.95
SES	−0.81	−0.89	−0.95	0.97	0.92	1.00	0.90
DEP	−0.75	−0.78	−0.86	0.93	0.95	0.90	1.00
**2010–2014 ^c^**	**MHI**	**MFI**	**SEA**	**SEP**	**SED**	**SES**	**DEP**
MHI	1.00	0.91	0.82	−0.86	−0.89	−0.81	−0.77
MFI	0.91	1.00	0.90	−0.90	−0.91	−0.91	−0.79
SEA	0.82	0.90	1.00	−0.93	−0.90	−0.96	−0.85
SEP	−0.86	−0.90	−0.93	1.00	0.96	0.97	0.92
SED	−0.89	−0.91	−0.90	0.96	1.00	0.92	0.95
SES	−0.81	−0.91	−0.96	0.97	0.92	1.00	0.87
DEP	−0.77	−0.79	−0.85	0.92	0.95	0.87	1.00
**2015–2019 ^d^**	**MHI**	**MFI**	**SEA**	**SEP**	**SED**	**SES**	**DEP**
MHI	1.00	0.90	0.84	−0.85	−0.89	−0.82	−0.78
MFI	0.90	1.00	0.91	−0.86	−0.91	−0.91	−0.79
SEA	0.84	0.91	1.00	−0.91	−0.91	−0.96	−0.85
SEP	−0.85	−0.86	−0.91	1.00	0.95	0.94	0.93
SED	−0.89	−0.91	−0.91	0.95	1.00	0.93	0.94
SES	−0.82	−0.91	−0.96	0.94	0.93	1.00	0.87
DEP	−0.78	−0.79	−0.85	0.93	0.94	0.87	1.00

^a^ Correlation matrix based on 400 census tracts; 3 out of 403 census tracts (0.74%) were omitted due to missing data. ^b^ Correlation matrix based on 398 census tracts; 5 out of 403 census tracts (1.24%) were omitted due to missing data. ^c^ Correlation matrix based on 478 census tracts; 8 out of 486 census tracts (1.65%) were omitted due to missing data. ^d^ Correlation matrix based on 476 census tracts; 10 out of 486 census tracts (2.06%) were omitted due to missing data. Abbreviations: MHI, Median Household Income; MFI, Median Family Income; SEA, Socioeconomic Advantage [20]; SEP, Socioeconomic Position [21]; SED, Socioeconomic Deprivation [22]; SES, Socioeconomic Status [23]; DEP, Deprivation [24].

**Table 9:** Relationships between simple and composite measures of neighborhood socioeconomic status in the San Jose–Sunnyvale–Santa Clara Metropolitan Statistical Area.

**2000 ^a^**	**MHI**	**MFI**	**SEA**	**SEP**	**SED**	**SES**	**DEP**
MHI	1.00	0.96	0.86	−0.86	−0.86	−0.83	−0.73
MFI	0.96	1.00	0.90	−0.91	−0.90	−0.90	−0.80
SEA	0.86	0.90	1.00	−0.95	−0.93	−0.97	−0.90
SEP	−0.86	−0.91	−0.95	1.00	0.97	0.98	0.94
SED	−0.86	−0.90	−0.93	0.97	1.00	0.94	0.96
SES	−0.83	−0.90	−0.97	0.98	0.94	1.00	0.93
DEP	−0.73	−0.80	−0.90	0.94	0.96	0.93	1.00
**2005–2009 ^b^**	**MHI**	**MFI**	**SEA**	**SEP**	**SED**	**SES**	**DEP**
MHI	1.00	0.95	0.88	−0.87	−0.88	−0.85	−0.78
MFI	0.95	1.00	0.91	−0.91	−0.91	−0.91	−0.83
SEA	0.88	0.91	1.00	−0.94	−0.93	−0.96	−0.88
SEP	−0.87	−0.91	−0.94	1.00	0.96	0.98	0.95
SED	−0.88	−0.91	−0.93	0.96	1.00	0.94	0.96
SES	−0.85	−0.91	−0.96	0.98	0.94	1.00	0.92
DEP	−0.78	−0.83	−0.88	0.95	0.96	0.92	1.00
**2010–2014 ^c^**	**MHI**	**MFI**	**SEA**	**SEP**	**SED**	**SES**	**DEP**
MHI	1.00	0.95	0.89	−0.89	−0.88	−0.86	−0.80
MFI	0.95	1.00	0.92	−0.92	−0.92	−0.92	−0.83
SEA	0.89	0.92	1.00	−0.96	−0.94	−0.97	−0.90
SEP	−0.89	−0.92	−0.96	1.00	0.97	0.98	0.95
SED	−0.88	−0.92	−0.94	0.97	1.00	0.95	0.97
SES	−0.86	−0.92	−0.97	0.98	0.95	1.00	0.92
DEP	−0.80	−0.83	−0.90	0.95	0.97	0.92	1.00
**2015–2019 ^d^**	**MHI**	**MFI**	**SEA**	**SEP**	**SED**	**SES**	**DEP**
MHI	1.00	0.95	0.90	−0.88	−0.90	−0.86	−0.79
MFI	0.95	1.00	0.93	−0.91	−0.94	−0.92	−0.84
SEA	0.90	0.93	1.00	−0.94	−0.93	−0.97	−0.88
SEP	−0.88	−0.91	−0.94	1.00	0.95	0.97	0.93
SED	−0.90	−0.94	−0.93	0.95	1.00	0.94	0.96
SES	−0.86	−0.92	−0.97	0.97	0.94	1.00	0.91
DEP	−0.79	−0.84	−0.88	0.93	0.96	0.91	1.00

^a^ Correlation matrix based on 345 census tracts; 4 out of 349 census tracts (1.15%) were omitted due to missing data. ^b^ Correlation matrix based on 345 census tracts; 4 out of 349 census tracts (1.15%) were omitted due to missing data. ^c^ Correlation matrix based on 380 census tracts; 3 out of 383 census tracts (0.78%) were omitted due to missing data. ^d^ Correlation matrix based on 378 census tracts; 5 out of 383 census tracts (1.31%) were omitted due to missing data. Abbreviations: MHI, Median Household Income; MFI, Median Family Income; SEA, Socioeconomic Advantage [20]; SEP, Socioeconomic Position [21]; SED, Socioeconomic Deprivation [22]; SES, Socioeconomic Status [23]; DEP, Deprivation [24].

**Table 10:** Relationships between simple and composite measures of neighborhood socioeconomic status in the Fresno Metropolitan Statistical Area.

**2000 ^a^**	**MHI**	**MFI**	**SEA**	**SEP**	**SED**	**SES**	**DEP**
MHI	1.00	0.98	0.91	−0.92	−0.91	−0.93	−0.85
MFI	0.98	1.00	0.93	−0.94	−0.93	−0.95	−0.87
SEA	0.91	0.93	1.00	−0.97	−0.96	−0.98	−0.94
SEP	−0.92	−0.94	−0.97	1.00	0.96	0.98	0.94
SED	−0.91	−0.93	−0.96	0.96	1.00	0.96	0.97
SES	−0.93	−0.95	−0.98	0.98	0.96	1.00	0.92
DEP	−0.85	−0.87	−0.94	0.94	0.97	0.92	1.00
**2005–2009 ^b^**	**MHI**	**MFI**	**SEA**	**SEP**	**SED**	**SES**	**DEP**
MHI	1.00	0.98	0.90	−0.93	−0.93	−0.93	−0.85
MFI	0.98	1.00	0.91	−0.93	−0.95	−0.95	−0.86
SEA	0.90	0.91	1.00	−0.95	−0.95	−0.97	−0.92
SEP	−0.93	−0.93	−0.95	1.00	0.96	0.99	0.94
SED	−0.93	−0.95	−0.95	0.96	1.00	0.96	0.96
SES	−0.93	−0.95	−0.97	0.99	0.96	1.00	0.92
DEP	−0.85	−0.86	−0.92	0.94	0.96	0.92	1.00
**2010–2014 ^c^**	**MHI**	**MFI**	**SEA**	**SEP**	**SED**	**SES**	**DEP**
MHI	1.00	0.97	0.90	−0.92	−0.94	−0.93	−0.87
MFI	0.97	1.00	0.91	−0.94	−0.96	−0.95	−0.89
SEA	0.90	0.91	1.00	−0.95	−0.95	−0.97	−0.92
SEP	−0.92	−0.94	−0.95	1.00	0.97	0.98	0.95
SED	−0.94	−0.96	−0.95	0.97	1.00	0.97	0.97
SES	−0.93	−0.95	−0.97	0.98	0.97	1.00	0.94
DEP	−0.87	−0.89	−0.92	0.95	0.97	0.94	1.00
**2015–2019 ^d^**	**MHI**	**MFI**	**SEA**	**SEP**	**SED**	**SES**	**DEP**
MHI	1.00	0.97	0.93	−0.91	−0.94	−0.93	−0.89
MFI	0.97	1.00	0.93	−0.92	−0.94	−0.95	−0.89
SEA	0.93	0.93	1.00	−0.95	−0.96	−0.97	−0.93
SEP	−0.91	−0.92	−0.95	1.00	0.95	0.97	0.94
SED	−0.94	−0.94	−0.96	0.95	1.00	0.97	0.97
SES	−0.93	−0.95	−0.97	0.97	0.97	1.00	0.93
DEP	−0.89	−0.89	−0.93	0.94	0.97	0.93	1.00

^a^ Correlation matrix based on 156 census tracts; 2 out of 158 census tracts (1.27%) were omitted due to missing data. ^b^ Correlation matrix based on 156 census tracts; 2 out of 158 census tracts (1.27%) were omitted due to missing data. ^c^ Correlation matrix based on 196 census tracts; 3 out of 199 census tracts (1.51%) were omitted due to missing data. ^d^ Correlation matrix based on 196 census tracts; 3 out of 199 census tracts (1.51%) were omitted due to missing data. Abbreviations: MHI, Median Household Income; MFI, Median Family Income; SEA, Socioeconomic Advantage [20]; SEP, Socioeconomic Position [21]; SED, Socioeconomic Deprivation [22]; SES, Socioeconomic Status [23]; DEP, Deprivation [24].

## Data Availability

Publicly available datasets were analyzed in this study. These data can be downloaded from the US Bureau’s website: https://www.census.gov/data.html (accessed on 20 November 2022).

## Supplementary Materials

The following supporting information can be downloaded at: https://www.mdpi.com/article/10.3390/ijerph20010211/s1, A series of correlation analyses for 47 states and the District of Columbia (excluding the State of California, which is shown in Table 3) in the conterminous US (i.e., the contiguous US) are provided in Tables S1–S48.
