# Supplementary material for: Census-Tract-Level Median Household Income and Median Family Income Estimates: A Unidimensional Measure of Neighborhood Socioeconomic Status?
Source: Int J Environ Res Public Health. 2022 Dec 23;20(1):211. doi: 10.3390/ijerph20010211 (PMC9819545; doi:10.3390/ijerph20010211)
Supplement: Supplementary file 1 [file ijerph-20-00211-s001.zip › ijerph-2078812-supplementary.pdf]

Table S1. Relationships between simple and composite measures of neighborhood socioeconomic status in the State of Alabama.

| 2000[a] | MHI | MFI | SEA | SEP | SED | SES | DEP |
|---|---|---|---|---|---|---|---|
| MHI | 1.00 | 0.95 | 0.91 | −0.92 | −0.92 | −0.89 | −0.86 |
| MFI | 0.95 | 1.00 | 0.94 | −0.93 | −0.93 | −0.94 | −0.84 |
| SEA | 0.91 | 0.94 | 1.00 | −0.95 | −0.94 | −0.97 | −0.86 |
| SEP | −0.92 | −0.93 | −0.95 | 1.00 | 0.95 | 0.97 | 0.90 |
| SED | −0.92 | −0.93 | −0.94 | 0.95 | 1.00 | 0.92 | 0.95 |
| SES | −0.89 | −0.94 | −0.97 | 0.97 | 0.92 | 1.00 | 0.83 |
| DEP | −0.86 | −0.84 | −0.86 | 0.90 | 0.95 | 0.83 | 1.00 |
| 2005–2009[b] | MHI | MFI | SEA | SEP | SED | SES | DEP |
| MHI | 1.00 | 0.91 | 0.90 | −0.89 | −0.91 | −0.87 | −0.84 |
| MFI | 0.91 | 1.00 | 0.91 | −0.91 | −0.91 | −0.93 | −0.81 |
| SEA | 0.90 | 0.91 | 1.00 | −0.95 | −0.93 | −0.96 | −0.85 |
| SEP | −0.89 | −0.91 | −0.95 | 1.00 | 0.96 | 0.97 | 0.91 |
| SED | −0.91 | −0.91 | −0.93 | 0.96 | 1.00 | 0.93 | 0.95 |
| SES | −0.87 | −0.93 | −0.96 | 0.97 | 0.93 | 1.00 | 0.83 |
| DEP | −0.84 | −0.81 | −0.85 | 0.91 | 0.95 | 0.83 | 1.00 |
| 2010–2014[c] | MHI | MFI | SEA | SEP | SED | SES | DEP |
| MHI | 1.00 | 0.92 | 0.90 | −0.90 | −0.93 | −0.88 | −0.88 |
| MFI | 0.92 | 1.00 | 0.92 | −0.91 | −0.94 | −0.93 | −0.85 |
| SEA | 0.90 | 0.92 | 1.00 | −0.95 | −0.94 | −0.96 | −0.87 |
| SEP | −0.90 | −0.91 | −0.95 | 1.00 | 0.96 | 0.97 | 0.92 |
| SED | −0.93 | −0.94 | −0.94 | 0.96 | 1.00 | 0.94 | 0.96 |
| SES | −0.88 | −0.93 | −0.96 | 0.97 | 0.94 | 1.00 | 0.86 |
| DEP | −0.88 | −0.85 | −0.87 | 0.92 | 0.96 | 0.86 | 1.00 |
| 2015–2019[d] | MHI | MFI | SEA | SEP | SED | SES | DEP |
| MHI | 1.00 | 0.93 | 0.90 | −0.89 | −0.93 | −0.88 | −0.86 |
| MFI | 0.93 | 1.00 | 0.92 | −0.90 | −0.94 | −0.94 | −0.83 |
| SEA | 0.90 | 0.92 | 1.00 | −0.92 | −0.94 | −0.96 | −0.87 |
| SEP | −0.89 | −0.90 | −0.92 | 1.00 | 0.95 | 0.95 | 0.91 |
| SED | −0.93 | −0.94 | −0.94 | 0.95 | 1.00 | 0.94 | 0.95 |
| SES | −0.88 | −0.94 | −0.96 | 0.95 | 0.94 | 1.00 | 0.85 |
| DEP | −0.86 | −0.83 | −0.87 | 0.91 | 0.95 | 0.85 | 1.00 |

[a] Correlation matrix based on 1,079 census tracts; 2 out of 1,081 census tracts (0.19%) were omitted due to missing data.

[b] Correlation matrix based on 1,075 census tracts; 7 out of 1,082 census tracts (0.65%) were omitted due to missing data.

[c] Correlation matrix based on 1,168 census tracts; 13 out of 1,181 census tracts (1.10%) were omitted due to missing data.

[d] Correlation matrix based on 1,155 census tracts; 26 out of 1,181 census tracts (2.20%) were omitted due to missing data.

Abbreviations: MHI, Median Household Income; MFI, Median Family Income; SEA, Socioeconomic Advantage [20]; SEP, Socioeconomic Position [21]; SED, Socioeconomic Deprivation [22]; SES, Socioeconomic Status [23]; DEP, Deprivation [24].

Table S2. Relationships between simple and composite measures of neighborhood socioeconomic status in the State of Arizona.

| 2000[a] | MHI | MFI | SEA | SEP | SED | SES | DEP |
|---|---|---|---|---|---|---|---|
| MHI | 1.00 | 0.96 | 0.86 | −0.88 | −0.85 | −0.88 | −0.73 |
| MFI | 0.96 | 1.00 | 0.89 | −0.91 | −0.87 | −0.93 | −0.76 |
| SEA | 0.86 | 0.89 | 1.00 | −0.94 | −0.94 | −0.97 | −0.89 |
| SEP | −0.88 | −0.91 | −0.94 | 1.00 | 0.96 | 0.97 | 0.92 |
| SED | −0.85 | −0.87 | −0.94 | 0.96 | 1.00 | 0.94 | 0.97 |
| SES | −0.88 | −0.93 | −0.97 | 0.97 | 0.94 | 1.00 | 0.88 |
| DEP | −0.73 | −0.76 | −0.89 | 0.92 | 0.97 | 0.88 | 1.00 |
| 2005–2009[b] | MHI | MFI | SEA | SEP | SED | SES | DEP |
| MHI | 1.00 | 0.91 | 0.85 | −0.87 | −0.86 | −0.86 | −0.74 |
| MFI | 0.91 | 1.00 | 0.87 | −0.88 | −0.87 | −0.92 | −0.76 |
| SEA | 0.85 | 0.87 | 1.00 | −0.94 | −0.93 | −0.96 | −0.88 |
| SEP | −0.87 | −0.88 | −0.94 | 1.00 | 0.95 | 0.98 | 0.92 |
| SED | −0.86 | −0.87 | −0.93 | 0.95 | 1.00 | 0.93 | 0.96 |
| SES | −0.86 | −0.92 | −0.96 | 0.98 | 0.93 | 1.00 | 0.88 |
| DEP | −0.74 | −0.76 | −0.88 | 0.92 | 0.96 | 0.88 | 1.00 |
| 2010–2014[c] | MHI | MFI | SEA | SEP | SED | SES | DEP |
| MHI | 1.00 | 0.94 | 0.84 | −0.88 | −0.87 | −0.87 | −0.77 |
| MFI | 0.94 | 1.00 | 0.88 | −0.90 | −0.90 | −0.92 | −0.79 |
| SEA | 0.84 | 0.88 | 1.00 | −0.94 | −0.92 | −0.96 | −0.87 |
| SEP | −0.88 | −0.90 | −0.94 | 1.00 | 0.96 | 0.98 | 0.92 |
| SED | −0.87 | −0.90 | −0.92 | 0.96 | 1.00 | 0.94 | 0.97 |
| SES | −0.87 | −0.92 | −0.96 | 0.98 | 0.94 | 1.00 | 0.88 |
| DEP | −0.77 | −0.79 | −0.87 | 0.92 | 0.97 | 0.88 | 1.00 |
| 2015–2019[d] | MHI | MFI | SEA | SEP | SED | SES | DEP |
| MHI | 1.00 | 0.94 | 0.86 | −0.88 | −0.87 | −0.87 | −0.74 |
| MFI | 0.94 | 1.00 | 0.90 | −0.89 | −0.89 | −0.93 | −0.77 |
| SEA | 0.86 | 0.90 | 1.00 | −0.92 | −0.93 | −0.95 | −0.87 |
| SEP | −0.88 | −0.89 | −0.92 | 1.00 | 0.95 | 0.97 | 0.91 |
| SED | −0.87 | −0.89 | −0.93 | 0.95 | 1.00 | 0.93 | 0.96 |
| SES | −0.87 | −0.93 | −0.95 | 0.97 | 0.93 | 1.00 | 0.87 |
| DEP | −0.74 | −0.77 | −0.87 | 0.91 | 0.96 | 0.87 | 1.00 |

[a] Correlation matrix based on 1,095 census tracts; 12 out of 1,107 census tracts (1.08%) were omitted due to missing data.

[b] Correlation matrix based on 1,085 census tracts; 22 out of 1,107 census tracts (1.99%) were omitted due to missing data.

[c] Correlation matrix based on 1,500 census tracts; 26 out of 1,526 census tracts (1.70%) were omitted due to missing data.

[d] Correlation matrix based on 1,481 census tracts; 45 out of 1,526 census tracts (2.95%) were omitted due to missing data.

Abbreviations: MHI, Median Household Income; MFI, Median Family Income; SEA, Socioeconomic Advantage [20]; SEP, Socioeconomic Position [21]; SED, Socioeconomic Deprivation [22]; SES, Socioeconomic Status [23]; DEP, Deprivation [24].

Table S3. Relationships between simple and composite measures of neighborhood socioeconomic status in the State of Arkansas.

| 2000[a] | MHI | MFI | SEA | SEP | SED | SES | DEP |
|---|---|---|---|---|---|---|---|
| MHI | 1.00 | 0.95 | 0.88 | −0.90 | −0.93 | −0.88 | −0.83 |
| MFI | 0.95 | 1.00 | 0.91 | −0.91 | −0.94 | −0.94 | −0.81 |
| SEA | 0.88 | 0.91 | 1.00 | −0.93 | −0.94 | −0.97 | −0.84 |
| SEP | −0.90 | −0.91 | −0.93 | 1.00 | 0.95 | 0.94 | 0.88 |
| SED | −0.93 | −0.94 | −0.94 | 0.95 | 1.00 | 0.94 | 0.92 |
| SES | −0.88 | −0.94 | −0.97 | 0.94 | 0.94 | 1.00 | 0.79 |
| DEP | −0.83 | −0.81 | −0.84 | 0.88 | 0.92 | 0.79 | 1.00 |
| 2005–2009[b] | MHI | MFI | SEA | SEP | SED | SES | DEP |
| MHI | 1.00 | 0.91 | 0.86 | −0.88 | −0.91 | −0.85 | −0.87 |
| MFI | 0.91 | 1.00 | 0.88 | −0.89 | −0.92 | −0.91 | −0.82 |
| SEA | 0.86 | 0.88 | 1.00 | −0.93 | −0.93 | −0.96 | −0.85 |
| SEP | −0.88 | −0.89 | −0.93 | 1.00 | 0.95 | 0.95 | 0.91 |
| SED | −0.91 | −0.92 | −0.93 | 0.95 | 1.00 | 0.93 | 0.95 |
| SES | −0.85 | −0.91 | −0.96 | 0.95 | 0.93 | 1.00 | 0.83 |
| DEP | −0.87 | −0.82 | −0.85 | 0.91 | 0.95 | 0.83 | 1.00 |
| 2010–2014[c] | MHI | MFI | SEA | SEP | SED | SES | DEP |
| MHI | 1.00 | 0.93 | 0.86 | −0.88 | −0.90 | −0.86 | −0.80 |
| MFI | 0.93 | 1.00 | 0.89 | −0.90 | −0.92 | −0.92 | −0.79 |
| SEA | 0.86 | 0.89 | 1.00 | −0.94 | −0.94 | −0.96 | −0.85 |
| SEP | −0.88 | −0.90 | −0.94 | 1.00 | 0.95 | 0.96 | 0.90 |
| SED | −0.90 | −0.92 | −0.94 | 0.95 | 1.00 | 0.94 | 0.93 |
| SES | −0.86 | −0.92 | −0.96 | 0.96 | 0.94 | 1.00 | 0.82 |
| DEP | −0.80 | −0.79 | −0.85 | 0.90 | 0.93 | 0.82 | 1.00 |
| 2015–2019[d] | MHI | MFI | SEA | SEP | SED | SES | DEP |
| MHI | 1.00 | 0.92 | 0.86 | −0.85 | −0.90 | −0.84 | −0.81 |
| MFI | 0.92 | 1.00 | 0.87 | −0.85 | −0.92 | −0.90 | −0.79 |
| SEA | 0.86 | 0.87 | 1.00 | −0.92 | −0.94 | −0.95 | −0.86 |
| SEP | −0.85 | −0.85 | −0.92 | 1.00 | 0.95 | 0.94 | 0.90 |
| SED | −0.90 | −0.92 | −0.94 | 0.95 | 1.00 | 0.94 | 0.94 |
| SES | −0.84 | −0.90 | −0.95 | 0.94 | 0.94 | 1.00 | 0.82 |
| DEP | −0.81 | −0.79 | −0.86 | 0.90 | 0.94 | 0.82 | 1.00 |

[a] Correlation matrix based on 619 census tracts; 5 out of 624 census tracts (0.80%) were omitted due to missing data.

[b] Correlation matrix based on 615 census tracts; 8 out of 623 census tracts (1.28%) were omitted due to missing data.

[c] Correlation matrix based on 681 census tracts; 5 out of 686 census tracts (0.73%) were omitted due to missing data.

[d] Correlation matrix based on 678 census tracts; 8 out of 686 census tracts (1.17%) were omitted due to missing data.

Abbreviations: MHI, Median Household Income; MFI, Median Family Income; SEA, Socioeconomic Advantage [20]; SEP, Socioeconomic Position [21]; SED, Socioeconomic Deprivation [22]; SES, Socioeconomic Status [23]; DEP, Deprivation [24].

Table S4. Relationships between simple and composite measures of neighborhood socioeconomic status in the State of Colorado.

| 2000[a] | MHI | MFI | SEA | SEP | SED | SES | DEP |
|---|---|---|---|---|---|---|---|
| MHI | 1.00 | 0.96 | 0.87 | −0.86 | −0.87 | −0.86 | −0.77 |
| MFI | 0.96 | 1.00 | 0.91 | −0.89 | −0.89 | −0.92 | −0.79 |
| SEA | 0.87 | 0.91 | 1.00 | −0.93 | −0.92 | −0.97 | −0.88 |
| SEP | −0.86 | −0.89 | −0.93 | 1.00 | 0.96 | 0.96 | 0.93 |
| SED | −0.87 | −0.89 | −0.92 | 0.96 | 1.00 | 0.93 | 0.97 |
| SES | −0.86 | −0.92 | −0.97 | 0.96 | 0.93 | 1.00 | 0.88 |
| DEP | −0.77 | −0.79 | −0.88 | 0.93 | 0.97 | 0.88 | 1.00 |
| 2005–2009[b] | MHI | MFI | SEA | SEP | SED | SES | DEP |
| MHI | 1.00 | 0.91 | 0.85 | −0.85 | −0.88 | −0.82 | −0.78 |
| MFI | 0.91 | 1.00 | 0.90 | −0.89 | −0.90 | −0.92 | −0.80 |
| SEA | 0.85 | 0.90 | 1.00 | −0.93 | −0.92 | −0.96 | −0.87 |
| SEP | −0.85 | −0.89 | −0.93 | 1.00 | 0.95 | 0.97 | 0.94 |
| SED | −0.88 | −0.90 | −0.92 | 0.95 | 1.00 | 0.92 | 0.96 |
| SES | −0.82 | −0.92 | −0.96 | 0.97 | 0.92 | 1.00 | 0.88 |
| DEP | −0.78 | −0.80 | −0.87 | 0.94 | 0.96 | 0.88 | 1.00 |
| 2010–2014[c] | MHI | MFI | SEA | SEP | SED | SES | DEP |
| MHI | 1.00 | 0.91 | 0.85 | −0.86 | −0.87 | −0.82 | −0.78 |
| MFI | 0.91 | 1.00 | 0.89 | −0.89 | −0.89 | −0.91 | −0.79 |
| SEA | 0.85 | 0.89 | 1.00 | −0.93 | −0.91 | −0.95 | −0.86 |
| SEP | −0.86 | −0.89 | −0.93 | 1.00 | 0.95 | 0.97 | 0.94 |
| SED | −0.87 | −0.89 | −0.91 | 0.95 | 1.00 | 0.92 | 0.96 |
| SES | −0.82 | −0.91 | −0.95 | 0.97 | 0.92 | 1.00 | 0.89 |
| DEP | −0.78 | −0.79 | −0.86 | 0.94 | 0.96 | 0.89 | 1.00 |
| 2015–2019[d] | MHI | MFI | SEA | SEP | SED | SES | DEP |
| MHI | 1.00 | 0.92 | 0.86 | −0.85 | −0.89 | −0.83 | −0.77 |
| MFI | 0.92 | 1.00 | 0.91 | −0.87 | −0.91 | −0.92 | −0.77 |
| SEA | 0.86 | 0.91 | 1.00 | −0.91 | −0.91 | −0.96 | −0.84 |
| SEP | −0.85 | −0.87 | −0.91 | 1.00 | 0.94 | 0.95 | 0.92 |
| SED | −0.89 | −0.91 | −0.91 | 0.94 | 1.00 | 0.92 | 0.94 |
| SES | −0.83 | −0.92 | −0.96 | 0.95 | 0.92 | 1.00 | 0.85 |
| DEP | −0.77 | −0.77 | −0.84 | 0.92 | 0.94 | 0.85 | 1.00 |

[a] Correlation matrix based on 1,050 census tracts; 12 out of 1,062 census tracts (1.13%) were omitted due to missing data.

[b] Correlation matrix based on 1,049 census tracts; 26 out of 1,075 census tracts (2.42%) were omitted due to missing data.

[c] Correlation matrix based on 1,220 census tracts; 29 out of 1,249 census tracts (2.32%) were omitted due to missing data.

[d] Correlation matrix based on 1,221 census tracts; 28 out of 1,249 census tracts (2.24%) were omitted due to missing data.

Abbreviations: MHI, Median Household Income; MFI, Median Family Income; SEA, Socioeconomic Advantage [20]; SEP, Socioeconomic Position [21]; SED, Socioeconomic Deprivation [22]; SES, Socioeconomic Status [23]; DEP, Deprivation [24].

Table S5. Relationships between simple and composite measures of neighborhood socioeconomic status in the State of Connecticut.

| 2000[a] | MHI | MFI | SEA | SEP | SED | SES | DEP |
|---|---|---|---|---|---|---|---|
| MHI | 1.00 | 0.98 | 0.90 | −0.90 | −0.80 | −0.92 | −0.70 |
| MFI | 0.98 | 1.00 | 0.92 | −0.92 | −0.83 | −0.95 | −0.73 |
| SEA | 0.90 | 0.92 | 1.00 | −0.96 | −0.93 | −0.97 | −0.88 |
| SEP | −0.90 | −0.92 | −0.96 | 1.00 | 0.95 | 0.98 | 0.90 |
| SED | −0.80 | −0.83 | −0.93 | 0.95 | 1.00 | 0.91 | 0.98 |
| SES | −0.92 | −0.95 | −0.97 | 0.98 | 0.91 | 1.00 | 0.85 |
| DEP | −0.70 | −0.73 | −0.88 | 0.90 | 0.98 | 0.85 | 1.00 |

| 2005–2009[b] | MHI | MFI | SEA | SEP | SED | SES | DEP |
|---|---|---|---|---|---|---|---|
| MHI | 1.00 | 0.97 | 0.90 | −0.89 | −0.82 | −0.89 | −0.70 |
| MFI | 0.97 | 1.00 | 0.92 | −0.91 | −0.84 | −0.93 | −0.72 |
| SEA | 0.90 | 0.92 | 1.00 | −0.96 | −0.92 | −0.97 | −0.84 |
| SEP | −0.89 | −0.91 | −0.96 | 1.00 | 0.96 | 0.99 | 0.91 |
| SED | −0.82 | −0.84 | −0.92 | 0.96 | 1.00 | 0.93 | 0.97 |
| SES | −0.89 | −0.93 | −0.97 | 0.99 | 0.93 | 1.00 | 0.86 |
| DEP | −0.70 | −0.72 | −0.84 | 0.91 | 0.97 | 0.86 | 1.00 |

| 2010–2014[c] | MHI | MFI | SEA | SEP | SED | SES | DEP |
|---|---|---|---|---|---|---|---|
| MHI | 1.00 | 0.96 | 0.90 | −0.89 | −0.83 | −0.88 | −0.72 |
| MFI | 0.96 | 1.00 | 0.94 | −0.92 | −0.87 | −0.93 | −0.76 |
| SEA | 0.90 | 0.94 | 1.00 | −0.96 | −0.93 | −0.98 | −0.86 |
| SEP | −0.89 | −0.92 | −0.96 | 1.00 | 0.96 | 0.99 | 0.92 |
| SED | −0.83 | −0.87 | −0.93 | 0.96 | 1.00 | 0.94 | 0.97 |
| SES | −0.88 | −0.93 | −0.98 | 0.99 | 0.94 | 1.00 | 0.89 |
| DEP | −0.72 | −0.76 | −0.86 | 0.92 | 0.97 | 0.89 | 1.00 |

| 2015–2019[d] | MHI | MFI | SEA | SEP | SED | SES | DEP |
|---|---|---|---|---|---|---|---|
| MHI | 1.00 | 0.96 | 0.92 | −0.89 | −0.85 | −0.89 | −0.74 |
| MFI | 0.96 | 1.00 | 0.94 | −0.91 | −0.89 | −0.94 | −0.78 |
| SEA | 0.92 | 0.94 | 1.00 | −0.94 | −0.91 | −0.96 | −0.84 |
| SEP | −0.89 | −0.91 | −0.94 | 1.00 | 0.95 | 0.98 | 0.92 |
| SED | −0.85 | −0.89 | −0.91 | 0.95 | 1.00 | 0.94 | 0.96 |
| SES | −0.89 | −0.94 | −0.96 | 0.98 | 0.94 | 1.00 | 0.88 |
| DEP | −0.74 | −0.78 | −0.84 | 0.92 | 0.96 | 0.88 | 1.00 |

[a] Correlation matrix based on 808 census tracts; 11 out of 819 census tracts (1.34%) were omitted due to missing data.

[b] Correlation matrix based on 800 census tracts; 19 out of 819 census tracts (2.32%) were omitted due to missing data.

[c] Correlation matrix based on 817 census tracts; 16 out of 833 census tracts (1.92%) were omitted due to missing data.

[d] Correlation matrix based on 813 census tracts; 20 out of 833 census tracts (2.40%) were omitted due to missing data.

Abbreviations: MHI, Median Household Income; MFI, Median Family Income; SEA, Socioeconomic Advantage [20]; SEP, Socioeconomic Position [21]; SED, Socioeconomic Deprivation [22]; SES, Socioeconomic Status [23]; DEP, Deprivation [24].

Table S6. Relationships between simple and composite measures of neighborhood socioeconomic status in the State of Delaware.

| 2000[a] | MHI | MFI | SEA | SEP | SED | SES | DEP |
|---|---|---|---|---|---|---|---|
| MHI | 1.00 | 0.95 | 0.91 | −0.87 | −0.87 | −0.90 | −0.75 |
| MFI | 0.95 | 1.00 | 0.94 | −0.89 | −0.87 | −0.95 | −0.76 |
| SEA | 0.91 | 0.94 | 1.00 | −0.93 | −0.92 | −0.97 | −0.84 |
| SEP | −0.87 | −0.89 | −0.93 | 1.00 | 0.95 | 0.96 | 0.90 |
| SED | −0.87 | −0.87 | −0.92 | 0.95 | 1.00 | 0.91 | 0.96 |
| SES | −0.90 | −0.95 | −0.97 | 0.96 | 0.91 | 1.00 | 0.83 |
| DEP | −0.75 | −0.76 | −0.84 | 0.90 | 0.96 | 0.83 | 1.00 |
| 2005–2009[b] | MHI | MFI | SEA | SEP | SED | SES | DEP |
| MHI | 1.00 | 0.90 | 0.88 | −0.86 | −0.88 | −0.85 | −0.78 |
| MFI | 0.90 | 1.00 | 0.89 | −0.85 | −0.84 | −0.91 | −0.75 |
| SEA | 0.88 | 0.89 | 1.00 | −0.95 | −0.92 | −0.97 | −0.87 |
| SEP | −0.86 | −0.85 | −0.95 | 1.00 | 0.94 | 0.97 | 0.91 |
| SED | −0.88 | −0.84 | −0.92 | 0.94 | 1.00 | 0.90 | 0.96 |
| SES | −0.85 | −0.91 | −0.97 | 0.97 | 0.90 | 1.00 | 0.86 |
| DEP | −0.78 | −0.75 | −0.87 | 0.91 | 0.96 | 0.86 | 1.00 |
| 2010–2014[c] | MHI | MFI | SEA | SEP | SED | SES | DEP |
| MHI | 1.00 | 0.91 | 0.85 | −0.86 | −0.86 | −0.82 | −0.80 |
| MFI | 0.91 | 1.00 | 0.88 | −0.88 | −0.83 | −0.91 | −0.77 |
| SEA | 0.85 | 0.88 | 1.00 | −0.95 | −0.89 | −0.96 | −0.88 |
| SEP | −0.86 | −0.88 | −0.95 | 1.00 | 0.94 | 0.97 | 0.93 |
| SED | −0.86 | −0.83 | −0.89 | 0.94 | 1.00 | 0.89 | 0.98 |
| SES | −0.82 | −0.91 | −0.96 | 0.97 | 0.89 | 1.00 | 0.88 |
| DEP | −0.80 | −0.77 | −0.88 | 0.93 | 0.98 | 0.88 | 1.00 |
| 2015–2019[d] | MHI | MFI | SEA | SEP | SED | SES | DEP |
| MHI | 1.00 | 0.91 | 0.89 | −0.88 | −0.90 | −0.85 | −0.82 |
| MFI | 0.91 | 1.00 | 0.92 | −0.89 | −0.90 | −0.93 | −0.82 |
| SEA | 0.89 | 0.92 | 1.00 | −0.93 | −0.90 | −0.96 | −0.86 |
| SEP | −0.88 | −0.89 | −0.93 | 1.00 | 0.93 | 0.96 | 0.91 |
| SED | −0.90 | −0.90 | −0.90 | 0.93 | 1.00 | 0.89 | 0.95 |
| SES | −0.85 | −0.93 | −0.96 | 0.96 | 0.89 | 1.00 | 0.84 |
| DEP | −0.82 | −0.82 | −0.86 | 0.91 | 0.95 | 0.84 | 1.00 |

[a] Correlation matrix based on 196 census tracts; 1 out of 197 census tracts (0.51%) were omitted due to missing data.

[b] Correlation matrix based on 193 census tracts; 4 out of 197 census tracts (2.03%) were omitted due to missing data.

[c] Correlation matrix based on 213 census tracts; 5 out of 218 census tracts (2.29%) were omitted due to missing data.

[d] Correlation matrix based on 212 census tracts; 6 out of 218 census tracts (2.75%) were omitted due to missing data.

Abbreviations: MHI, Median Household Income; MFI, Median Family Income; SEA, Socioeconomic Advantage [20]; SEP, Socioeconomic Position [21]; SED, Socioeconomic Deprivation [22]; SES, Socioeconomic Status [23]; DEP, Deprivation [24].

Table S7. Relationships between simple and composite measures of neighborhood socioeconomic status in the District of Columbia.

| 2000[a] | MHI | MFI | SEA | SEP | SED | SES | DEP |
|---|---|---|---|---|---|---|---|
| MHI | 1.00 | 0.91 | 0.92 | −0.90 | −0.88 | −0.88 | −0.79 |
| MFI | 0.91 | 1.00 | 0.93 | −0.92 | −0.90 | −0.94 | −0.81 |
| SEA | 0.92 | 0.93 | 1.00 | −0.96 | −0.94 | −0.98 | −0.88 |
| SEP | −0.90 | −0.92 | −0.96 | 1.00 | 0.98 | 0.98 | 0.93 |
| SED | −0.88 | −0.90 | −0.94 | 0.98 | 1.00 | 0.95 | 0.96 |
| SES | −0.88 | −0.94 | −0.98 | 0.98 | 0.95 | 1.00 | 0.89 |
| DEP | −0.79 | −0.81 | −0.88 | 0.93 | 0.96 | 0.89 | 1.00 |
| 2005–2009[b] | MHI | MFI | SEA | SEP | SED | SES | DEP |
| MHI | 1.00 | 0.90 | 0.92 | −0.92 | −0.91 | −0.89 | −0.82 |
| MFI | 0.90 | 1.00 | 0.92 | −0.92 | −0.92 | −0.94 | −0.83 |
| SEA | 0.92 | 0.92 | 1.00 | −0.97 | −0.95 | −0.98 | −0.89 |
| SEP | −0.92 | −0.92 | −0.97 | 1.00 | 0.98 | 0.98 | 0.93 |
| SED | −0.91 | −0.92 | −0.95 | 0.98 | 1.00 | 0.97 | 0.96 |
| SES | −0.89 | −0.94 | −0.98 | 0.98 | 0.97 | 1.00 | 0.91 |
| DEP | −0.82 | −0.83 | −0.89 | 0.93 | 0.96 | 0.91 | 1.00 |
| 2010–2014[c] | MHI | MFI | SEA | SEP | SED | SES | DEP |
| MHI | 1.00 | 0.89 | 0.92 | −0.92 | −0.91 | −0.90 | −0.83 |
| MFI | 0.89 | 1.00 | 0.91 | −0.91 | −0.91 | −0.94 | −0.82 |
| SEA | 0.92 | 0.91 | 1.00 | −0.97 | −0.96 | −0.98 | −0.91 |
| SEP | −0.92 | −0.91 | −0.97 | 1.00 | 0.98 | 0.99 | 0.95 |
| SED | −0.91 | −0.91 | −0.96 | 0.98 | 1.00 | 0.97 | 0.96 |
| SES | −0.90 | −0.94 | −0.98 | 0.99 | 0.97 | 1.00 | 0.92 |
| DEP | −0.83 | −0.82 | −0.91 | 0.95 | 0.96 | 0.92 | 1.00 |
| 2015–2019[d] | MHI | MFI | SEA | SEP | SED | SES | DEP |
| MHI | 1.00 | 0.90 | 0.94 | −0.89 | −0.91 | −0.88 | −0.83 |
| MFI | 0.90 | 1.00 | 0.92 | −0.92 | −0.95 | −0.94 | −0.85 |
| SEA | 0.94 | 0.92 | 1.00 | −0.95 | −0.95 | −0.97 | −0.90 |
| SEP | −0.89 | −0.92 | −0.95 | 1.00 | 0.97 | 0.98 | 0.94 |
| SED | −0.91 | −0.95 | −0.95 | 0.97 | 1.00 | 0.97 | 0.95 |
| SES | −0.88 | −0.94 | −0.97 | 0.98 | 0.97 | 1.00 | 0.93 |
| DEP | −0.83 | −0.85 | −0.90 | 0.94 | 0.95 | 0.93 | 1.00 |

[a] Correlation matrix based on 178 census tracts; 10 out of 188 census tracts (5.32%) were omitted due to missing data.

[b] Correlation matrix based on 174 census tracts; 14 out of 188 census tracts (7.45%) were omitted due to missing data.

[c] Correlation matrix based on 174 census tracts; 5 out of 179 census tracts (2.79%) were omitted due to missing data.

[d] Correlation matrix based on 169 census tracts; 10 out of 179 census tracts (5.59%) were omitted due to missing data.

Abbreviations: MHI, Median Household Income; MFI, Median Family Income; SEA, Socioeconomic Advantage [20]; SEP, Socioeconomic Position [21]; SED, Socioeconomic Deprivation [22]; SES, Socioeconomic Status [23]; DEP, Deprivation [24].

Table S8. Relationships between simple and composite measures of neighborhood socioeconomic status in the State of Florida.

| 2000[a] | MHI | MFI | SEA | SEP | SED | SES | DEP |
|---|---|---|---|---|---|---|---|
| MHI | 1.00 | 0.95 | 0.87 | −0.87 | −0.82 | −0.89 | −0.71 |
| MFI | 0.95 | 1.00 | 0.90 | −0.90 | −0.84 | −0.94 | −0.73 |
| SEA | 0.87 | 0.90 | 1.00 | −0.95 | −0.93 | −0.96 | −0.88 |
| SEP | −0.87 | −0.90 | −0.95 | 1.00 | 0.95 | 0.97 | 0.91 |
| SED | −0.82 | −0.84 | −0.93 | 0.95 | 1.00 | 0.91 | 0.97 |
| SES | −0.89 | −0.94 | −0.96 | 0.97 | 0.91 | 1.00 | 0.85 |
| DEP | −0.71 | −0.73 | −0.88 | 0.91 | 0.97 | 0.85 | 1.00 |
| 2005–2009[b] | MHI | MFI | SEA | SEP | SED | SES | DEP |
| MHI | 1.00 | 0.91 | 0.86 | −0.87 | −0.85 | −0.86 | −0.75 |
| MFI | 0.91 | 1.00 | 0.89 | −0.88 | −0.85 | −0.92 | −0.75 |
| SEA | 0.86 | 0.89 | 1.00 | −0.94 | −0.91 | −0.96 | −0.86 |
| SEP | −0.87 | −0.88 | −0.94 | 1.00 | 0.95 | 0.97 | 0.93 |
| SED | −0.85 | −0.85 | −0.91 | 0.95 | 1.00 | 0.91 | 0.97 |
| SES | −0.86 | −0.92 | −0.96 | 0.97 | 0.91 | 1.00 | 0.86 |
| DEP | −0.75 | −0.75 | −0.86 | 0.93 | 0.97 | 0.86 | 1.00 |
| 2010–2014[c] | MHI | MFI | SEA | SEP | SED | SES | DEP |
| MHI | 1.00 | 0.92 | 0.86 | −0.87 | −0.86 | −0.86 | −0.75 |
| MFI | 0.92 | 1.00 | 0.89 | −0.90 | −0.88 | −0.92 | −0.77 |
| SEA | 0.86 | 0.89 | 1.00 | −0.94 | −0.92 | −0.95 | −0.87 |
| SEP | −0.87 | −0.90 | −0.94 | 1.00 | 0.96 | 0.98 | 0.92 |
| SED | −0.86 | −0.88 | −0.92 | 0.96 | 1.00 | 0.93 | 0.96 |
| SES | −0.86 | −0.92 | −0.95 | 0.98 | 0.93 | 1.00 | 0.87 |
| DEP | −0.75 | −0.77 | −0.87 | 0.92 | 0.96 | 0.87 | 1.00 |
| 2015–2019[d] | MHI | MFI | SEA | SEP | SED | SES | DEP |
| MHI | 1.00 | 0.93 | 0.87 | −0.87 | −0.86 | −0.87 | −0.75 |
| MFI | 0.93 | 1.00 | 0.90 | −0.88 | −0.88 | −0.92 | −0.77 |
| SEA | 0.87 | 0.90 | 1.00 | −0.92 | −0.92 | −0.95 | −0.86 |
| SEP | −0.87 | −0.88 | −0.92 | 1.00 | 0.95 | 0.97 | 0.92 |
| SED | −0.86 | −0.88 | −0.92 | 0.95 | 1.00 | 0.93 | 0.96 |
| SES | −0.87 | −0.92 | −0.95 | 0.97 | 0.93 | 1.00 | 0.87 |
| DEP | −0.75 | −0.77 | −0.86 | 0.92 | 0.96 | 0.87 | 1.00 |

[a] Correlation matrix based on 3,137 census tracts; 17 out of 3,154 census tracts (0.54%) were omitted due to missing data.

[b] Correlation matrix based on 3,129 census tracts; 25 out of 3,154 census tracts (0.79%) were omitted due to missing data.

[c] Correlation matrix based on 4,134 census tracts; 111 out of 4,245 census tracts (2.61%) were omitted due to missing data.

[d] Correlation matrix based on 4,113 census tracts; 132 out of 4,245 census tracts (3.11%) were omitted due to missing data.

Abbreviations: MHI, Median Household Income; MFI, Median Family Income; SEA, Socioeconomic Advantage [20]; SEP, Socioeconomic Position [21]; SED, Socioeconomic Deprivation [22]; SES, Socioeconomic Status [23]; DEP, Deprivation [24].

Table S9. Relationships between simple and composite measures of neighborhood socioeconomic status in the State of Georgia.

| 2000[a] | MHI | MFI | SEA | SEP | SED | SES | DEP |
|---|---|---|---|---|---|---|---|
| MHI | 1.00 | 0.93 | 0.90 | −0.89 | −0.90 | −0.88 | −0.82 |
| MFI | 0.93 | 1.00 | 0.92 | −0.90 | −0.90 | −0.94 | −0.80 |
| SEA | 0.90 | 0.92 | 1.00 | −0.93 | −0.94 | −0.97 | −0.86 |
| SEP | −0.89 | −0.90 | −0.93 | 1.00 | 0.96 | 0.95 | 0.92 |
| SED | −0.90 | −0.90 | −0.94 | 0.96 | 1.00 | 0.93 | 0.95 |
| SES | −0.88 | −0.94 | −0.97 | 0.95 | 0.93 | 1.00 | 0.83 |
| DEP | −0.82 | −0.80 | −0.86 | 0.92 | 0.95 | 0.83 | 1.00 |
| 2005–2009[b] | MHI | MFI | SEA | SEP | SED | SES | DEP |
| MHI | 1.00 | 0.91 | 0.89 | −0.89 | −0.90 | −0.86 | −0.83 |
| MFI | 0.91 | 1.00 | 0.89 | −0.90 | −0.88 | −0.92 | −0.79 |
| SEA | 0.89 | 0.89 | 1.00 | −0.93 | −0.92 | −0.96 | −0.85 |
| SEP | −0.89 | −0.90 | −0.93 | 1.00 | 0.95 | 0.96 | 0.91 |
| SED | −0.90 | −0.88 | −0.92 | 0.95 | 1.00 | 0.91 | 0.96 |
| SES | −0.86 | −0.92 | −0.96 | 0.96 | 0.91 | 1.00 | 0.83 |
| DEP | −0.83 | −0.79 | −0.85 | 0.91 | 0.96 | 0.83 | 1.00 |
| 2010–2014[c] | MHI | MFI | SEA | SEP | SED | SES | DEP |
| MHI | 1.00 | 0.91 | 0.90 | −0.89 | −0.91 | −0.87 | −0.85 |
| MFI | 0.91 | 1.00 | 0.90 | −0.90 | −0.90 | −0.93 | −0.81 |
| SEA | 0.90 | 0.90 | 1.00 | −0.95 | −0.94 | −0.96 | −0.88 |
| SEP | −0.89 | −0.90 | −0.95 | 1.00 | 0.96 | 0.97 | 0.93 |
| SED | −0.91 | −0.90 | −0.94 | 0.96 | 1.00 | 0.94 | 0.97 |
| SES | −0.87 | −0.93 | −0.96 | 0.97 | 0.94 | 1.00 | 0.88 |
| DEP | −0.85 | −0.81 | −0.88 | 0.93 | 0.97 | 0.88 | 1.00 |
| 2015–2019[d] | MHI | MFI | SEA | SEP | SED | SES | DEP |
| MHI | 1.00 | 0.92 | 0.90 | −0.88 | −0.92 | −0.88 | −0.85 |
| MFI | 0.92 | 1.00 | 0.92 | −0.89 | −0.92 | −0.94 | −0.81 |
| SEA | 0.90 | 0.92 | 1.00 | −0.93 | −0.94 | −0.97 | −0.88 |
| SEP | −0.88 | −0.89 | −0.93 | 1.00 | 0.95 | 0.96 | 0.92 |
| SED | −0.92 | −0.92 | −0.94 | 0.95 | 1.00 | 0.94 | 0.96 |
| SES | −0.88 | −0.94 | −0.97 | 0.96 | 0.94 | 1.00 | 0.86 |
| DEP | −0.85 | −0.81 | −0.88 | 0.92 | 0.96 | 0.86 | 1.00 |

[a] Correlation matrix based on 1,606 census tracts; 12 out of 1,618 census tracts (0.74%) were omitted due to missing data.

[b] Correlation matrix based on 1,598 census tracts; 20 out of 1,618 census tracts (1.24%) were omitted due to missing data.

[c] Correlation matrix based on 1,930 census tracts; 39 out of 1,969 census tracts (1.98%) were omitted due to missing data.

[d] Correlation matrix based on 1,911 census tracts; 58 out of 1,969 census tracts (2.95%) were omitted due to missing data.

Abbreviations: MHI, Median Household Income; MFI, Median Family Income; SEA, Socioeconomic Advantage [20]; SEP, Socioeconomic Position [21]; SED, Socioeconomic Deprivation [22]; SES, Socioeconomic Status [23]; DEP, Deprivation [24].

Table S10. Relationships between simple and composite measures of neighborhood socioeconomic status in the State of Idaho.

| 2000[a] | MHI | MFI | SEA | SEP | SED | SES | DEP |
|---|---|---|---|---|---|---|---|
| MHI | 1.00 | 0.93 | 0.86 | −0.87 | −0.92 | −0.85 | −0.87 |
| MFI | 0.93 | 1.00 | 0.89 | −0.87 | −0.89 | −0.90 | −0.81 |
| SEA | 0.86 | 0.89 | 1.00 | −0.92 | −0.90 | −0.95 | −0.84 |
| SEP | −0.87 | −0.87 | −0.92 | 1.00 | 0.94 | 0.96 | 0.93 |
| SED | −0.92 | −0.89 | −0.90 | 0.94 | 1.00 | 0.90 | 0.94 |
| SES | −0.85 | −0.90 | −0.95 | 0.96 | 0.90 | 1.00 | 0.84 |
| DEP | −0.87 | −0.81 | −0.84 | 0.93 | 0.94 | 0.84 | 1.00 |
| 2005–2009[b] | MHI | MFI | SEA | SEP | SED | SES | DEP |
| MHI | 1.00 | 0.89 | 0.81 | −0.83 | −0.89 | −0.79 | −0.82 |
| MFI | 0.89 | 1.00 | 0.83 | −0.85 | −0.92 | −0.88 | −0.80 |
| SEA | 0.81 | 0.83 | 1.00 | −0.88 | −0.85 | −0.93 | −0.75 |
| SEP | −0.83 | −0.85 | −0.88 | 1.00 | 0.92 | 0.95 | 0.88 |
| SED | −0.89 | −0.92 | −0.85 | 0.92 | 1.00 | 0.88 | 0.93 |
| SES | −0.79 | −0.88 | −0.93 | 0.95 | 0.88 | 1.00 | 0.75 |
| DEP | −0.82 | −0.80 | −0.75 | 0.88 | 0.93 | 0.75 | 1.00 |
| 2010–2014[c] | MHI | MFI | SEA | SEP | SED | SES | DEP |
| MHI | 1.00 | 0.92 | 0.84 | −0.84 | −0.90 | −0.79 | −0.81 |
| MFI | 0.92 | 1.00 | 0.87 | −0.86 | −0.91 | −0.88 | −0.79 |
| SEA | 0.84 | 0.87 | 1.00 | −0.94 | −0.92 | −0.94 | −0.84 |
| SEP | −0.84 | −0.86 | −0.94 | 1.00 | 0.94 | 0.96 | 0.87 |
| SED | −0.90 | −0.91 | −0.92 | 0.94 | 1.00 | 0.91 | 0.92 |
| SES | −0.79 | −0.88 | −0.94 | 0.96 | 0.91 | 1.00 | 0.77 |
| DEP | −0.81 | −0.79 | −0.84 | 0.87 | 0.92 | 0.77 | 1.00 |
| 2015–2019[d] | MHI | MFI | SEA | SEP | SED | SES | DEP |
| MHI | 1.00 | 0.88 | 0.84 | −0.84 | −0.90 | −0.77 | −0.82 |
| MFI | 0.88 | 1.00 | 0.89 | −0.84 | −0.92 | −0.89 | −0.79 |
| SEA | 0.84 | 0.89 | 1.00 | −0.90 | −0.91 | −0.94 | −0.83 |
| SEP | −0.84 | −0.84 | −0.90 | 1.00 | 0.93 | 0.91 | 0.88 |
| SED | −0.90 | −0.92 | −0.91 | 0.93 | 1.00 | 0.90 | 0.91 |
| SES | −0.77 | −0.89 | −0.94 | 0.91 | 0.90 | 1.00 | 0.77 |
| DEP | −0.82 | −0.79 | −0.83 | 0.88 | 0.91 | 0.77 | 1.00 |

[a] Correlation matrix based on 280 census tracts; out of 280 census tracts (0.00%) were omitted due to missing data.

[b] Correlation matrix based on 279 census tracts; 1 out of 280 census tracts (0.36%) were omitted due to missing data.

[c] Correlation matrix based on 296 census tracts; 2 out of 298 census tracts (0.67%) were omitted due to missing data.

[d] Correlation matrix based on 295 census tracts; 3 out of 298 census tracts (1.01%) were omitted due to missing data.

Abbreviations: MHI, Median Household Income; MFI, Median Family Income; SEA, Socioeconomic Advantage [20]; SEP, Socioeconomic Position [21]; SED, Socioeconomic Deprivation [22]; SES, Socioeconomic Status [23]; DEP, Deprivation [24].

Table S11. Relationships between simple and composite measures of neighborhood socioeconomic status in the State of Illinois.

| 2000[a] | MHI | MFI | SEA | SEP | SED | SES | DEP |
|---|---|---|---|---|---|---|---|
| MHI | 1.00 | 0.94 | 0.88 | −0.87 | −0.83 | −0.87 | −0.72 |
| MFI | 0.94 | 1.00 | 0.90 | −0.89 | −0.84 | −0.92 | −0.72 |
| SEA | 0.88 | 0.90 | 1.00 | −0.94 | −0.91 | −0.97 | −0.84 |
| SEP | −0.87 | −0.89 | −0.94 | 1.00 | 0.94 | 0.96 | 0.90 |
| SED | −0.83 | −0.84 | −0.91 | 0.94 | 1.00 | 0.89 | 0.96 |
| SES | −0.87 | −0.92 | −0.97 | 0.96 | 0.89 | 1.00 | 0.81 |
| DEP | −0.72 | −0.72 | −0.84 | 0.90 | 0.96 | 0.81 | 1.00 |
| 2005–2009[b] | MHI | MFI | SEA | SEP | SED | SES | DEP |
| MHI | 1.00 | 0.91 | 0.88 | −0.87 | −0.85 | −0.84 | −0.74 |
| MFI | 0.91 | 1.00 | 0.89 | −0.88 | −0.85 | −0.91 | −0.73 |
| SEA | 0.88 | 0.89 | 1.00 | −0.93 | −0.89 | −0.96 | −0.81 |
| SEP | −0.87 | −0.88 | −0.93 | 1.00 | 0.93 | 0.97 | 0.89 |
| SED | −0.85 | −0.85 | −0.89 | 0.93 | 1.00 | 0.87 | 0.95 |
| SES | −0.84 | −0.91 | −0.96 | 0.97 | 0.87 | 1.00 | 0.80 |
| DEP | −0.74 | −0.73 | −0.81 | 0.89 | 0.95 | 0.80 | 1.00 |
| 2010–2014[c] | MHI | MFI | SEA | SEP | SED | SES | DEP |
| MHI | 1.00 | 0.91 | 0.89 | −0.88 | −0.86 | −0.85 | −0.76 |
| MFI | 0.91 | 1.00 | 0.91 | −0.91 | −0.88 | −0.93 | −0.77 |
| SEA | 0.89 | 0.91 | 1.00 | −0.95 | −0.91 | −0.96 | −0.84 |
| SEP | −0.88 | −0.91 | −0.95 | 1.00 | 0.95 | 0.97 | 0.91 |
| SED | −0.86 | −0.88 | −0.91 | 0.95 | 1.00 | 0.91 | 0.96 |
| SES | −0.85 | −0.93 | −0.96 | 0.97 | 0.91 | 1.00 | 0.84 |
| DEP | −0.76 | −0.77 | −0.84 | 0.91 | 0.96 | 0.84 | 1.00 |
| 2015–2019[d] | MHI | MFI | SEA | SEP | SED | SES | DEP |
| MHI | 1.00 | 0.92 | 0.90 | −0.88 | −0.89 | −0.87 | −0.79 |
| MFI | 0.92 | 1.00 | 0.91 | −0.90 | −0.91 | −0.94 | −0.77 |
| SEA | 0.90 | 0.91 | 1.00 | −0.93 | −0.92 | −0.96 | −0.85 |
| SEP | −0.88 | −0.90 | −0.93 | 1.00 | 0.94 | 0.96 | 0.91 |
| SED | −0.89 | −0.91 | −0.92 | 0.94 | 1.00 | 0.91 | 0.95 |
| SES | −0.87 | −0.94 | −0.96 | 0.96 | 0.91 | 1.00 | 0.83 |
| DEP | −0.79 | −0.77 | −0.85 | 0.91 | 0.95 | 0.83 | 1.00 |

[a] Correlation matrix based on 2,927 census tracts; 39 out of 2,966 census tracts (1.31%) were omitted due to missing data.

[b] Correlation matrix based on 2,897 census tracts; 69 out of 2,966 census tracts (2.33%) were omitted due to missing data.

[c] Correlation matrix based on 3,097 census tracts; 26 out of 3,123 census tracts (0.83%) were omitted due to missing data.

[d] Correlation matrix based on 3,072 census tracts; 51 out of 3,123 census tracts (1.63%) were omitted due to missing data.

Abbreviations: MHI, Median Household Income; MFI, Median Family Income; SEA, Socioeconomic Advantage [20]; SEP, Socioeconomic Position [21]; SED, Socioeconomic Deprivation [22]; SES, Socioeconomic Status [23]; DEP, Deprivation [24].

Table S12. Relationships between simple and composite measures of neighborhood socioeconomic status in the State of Indiana.

| 2000[a] | MHI | MFI | SEA | SEP | SED | SES | DEP |
|---|---|---|---|---|---|---|---|
| MHI | 1.00 | 0.94 | 0.88 | −0.89 | −0.90 | −0.87 | −0.82 |
| MFI | 0.94 | 1.00 | 0.91 | −0.90 | −0.92 | −0.93 | −0.82 |
| SEA | 0.88 | 0.91 | 1.00 | −0.94 | −0.93 | −0.97 | −0.88 |
| SEP | −0.89 | −0.90 | −0.94 | 1.00 | 0.94 | 0.95 | 0.91 |
| SED | −0.90 | −0.92 | −0.93 | 0.94 | 1.00 | 0.93 | 0.94 |
| SES | −0.87 | −0.93 | −0.97 | 0.95 | 0.93 | 1.00 | 0.85 |
| DEP | −0.82 | −0.82 | −0.88 | 0.91 | 0.94 | 0.85 | 1.00 |
| 2005–2009[b] | MHI | MFI | SEA | SEP | SED | SES | DEP |
| MHI | 1.00 | 0.90 | 0.88 | −0.88 | −0.90 | −0.84 | −0.84 |
| MFI | 0.90 | 1.00 | 0.90 | −0.87 | −0.90 | −0.92 | −0.82 |
| SEA | 0.88 | 0.90 | 1.00 | −0.94 | −0.93 | −0.95 | −0.88 |
| SEP | −0.88 | −0.87 | −0.94 | 1.00 | 0.96 | 0.96 | 0.93 |
| SED | −0.90 | −0.90 | −0.93 | 0.96 | 1.00 | 0.93 | 0.95 |
| SES | −0.84 | −0.92 | −0.95 | 0.96 | 0.93 | 1.00 | 0.86 |
| DEP | −0.84 | −0.82 | −0.88 | 0.93 | 0.95 | 0.86 | 1.00 |
| 2010–2014[c] | MHI | MFI | SEA | SEP | SED | SES | DEP |
| MHI | 1.00 | 0.94 | 0.89 | −0.89 | −0.92 | −0.87 | −0.84 |
| MFI | 0.94 | 1.00 | 0.92 | −0.91 | −0.95 | −0.93 | −0.85 |
| SEA | 0.89 | 0.92 | 1.00 | −0.94 | −0.93 | −0.96 | −0.89 |
| SEP | −0.89 | −0.91 | −0.94 | 1.00 | 0.95 | 0.96 | 0.93 |
| SED | −0.92 | −0.95 | −0.93 | 0.95 | 1.00 | 0.94 | 0.96 |
| SES | −0.87 | −0.93 | −0.96 | 0.96 | 0.94 | 1.00 | 0.89 |
| DEP | −0.84 | −0.85 | −0.89 | 0.93 | 0.96 | 0.89 | 1.00 |
| 2015–2019[d] | MHI | MFI | SEA | SEP | SED | SES | DEP |
| MHI | 1.00 | 0.93 | 0.89 | −0.87 | −0.90 | −0.87 | −0.84 |
| MFI | 0.93 | 1.00 | 0.92 | −0.88 | −0.94 | −0.93 | −0.83 |
| SEA | 0.89 | 0.92 | 1.00 | −0.93 | −0.93 | −0.96 | −0.89 |
| SEP | −0.87 | −0.88 | −0.93 | 1.00 | 0.94 | 0.94 | 0.92 |
| SED | −0.90 | −0.94 | −0.93 | 0.94 | 1.00 | 0.94 | 0.95 |
| SES | −0.87 | −0.93 | −0.96 | 0.94 | 0.94 | 1.00 | 0.86 |
| DEP | −0.84 | −0.83 | −0.89 | 0.92 | 0.95 | 0.86 | 1.00 |

[a] Correlation matrix based on 1,404 census tracts; 10 out of 1,414 census tracts (0.71%) were omitted due to missing data.

[b] Correlation matrix based on 1,395 census tracts; 19 out of 1,414 census tracts (1.34%) were omitted due to missing data.

[c] Correlation matrix based on 1,494 census tracts; 17 out of 1,511 census tracts (1.13%) were omitted due to missing data.

[d] Correlation matrix based on 1,483 census tracts; 28 out of 1,511 census tracts (1.85%) were omitted due to missing data.

Abbreviations: MHI, Median Household Income; MFI, Median Family Income; SEA, Socioeconomic Advantage [20]; SEP, Socioeconomic Position [21]; SED, Socioeconomic Deprivation [22]; SES, Socioeconomic Status [23]; DEP, Deprivation [24].

Table S13. Relationships between simple and composite measures of neighborhood socioeconomic status in the State of Iowa.

| 2000[a] | MHI | MFI | SEA | SEP | SED | SES | DEP |
|---|---|---|---|---|---|---|---|
| MHI | 1.00 | 0.91 | 0.85 | −0.83 | −0.89 | −0.83 | −0.73 |
| MFI | 0.91 | 1.00 | 0.89 | −0.80 | −0.89 | −0.92 | −0.71 |
| SEA | 0.85 | 0.89 | 1.00 | −0.90 | −0.93 | −0.96 | −0.85 |
| SEP | −0.83 | −0.80 | −0.90 | 1.00 | 0.93 | 0.90 | 0.91 |
| SED | −0.89 | −0.89 | −0.93 | 0.93 | 1.00 | 0.93 | 0.90 |
| SES | −0.83 | −0.92 | −0.96 | 0.90 | 0.93 | 1.00 | 0.82 |
| DEP | −0.73 | −0.71 | −0.85 | 0.91 | 0.90 | 0.82 | 1.00 |
| 2005–2009[b] | MHI | MFI | SEA | SEP | SED | SES | DEP |
| MHI | 1.00 | 0.87 | 0.85 | −0.85 | −0.88 | −0.82 | −0.79 |
| MFI | 0.87 | 1.00 | 0.87 | −0.82 | −0.88 | −0.91 | −0.75 |
| SEA | 0.85 | 0.87 | 1.00 | −0.91 | −0.90 | −0.95 | −0.84 |
| SEP | −0.85 | −0.82 | −0.91 | 1.00 | 0.94 | 0.91 | 0.92 |
| SED | −0.88 | −0.88 | −0.90 | 0.94 | 1.00 | 0.90 | 0.94 |
| SES | −0.82 | −0.91 | −0.95 | 0.91 | 0.90 | 1.00 | 0.82 |
| DEP | −0.79 | −0.75 | −0.84 | 0.92 | 0.94 | 0.82 | 1.00 |
| 2010–2014[c] | MHI | MFI | SEA | SEP | SED | SES | DEP |
| MHI | 1.00 | 0.89 | 0.86 | −0.84 | −0.88 | −0.82 | −0.77 |
| MFI | 0.89 | 1.00 | 0.89 | −0.85 | −0.91 | −0.91 | −0.77 |
| SEA | 0.86 | 0.89 | 1.00 | −0.92 | −0.92 | −0.95 | −0.85 |
| SEP | −0.84 | −0.85 | −0.92 | 1.00 | 0.94 | 0.93 | 0.92 |
| SED | −0.88 | −0.91 | −0.92 | 0.94 | 1.00 | 0.92 | 0.92 |
| SES | −0.82 | −0.91 | −0.95 | 0.93 | 0.92 | 1.00 | 0.84 |
| DEP | −0.77 | −0.77 | −0.85 | 0.92 | 0.92 | 0.84 | 1.00 |
| 2015–2019[d] | MHI | MFI | SEA | SEP | SED | SES | DEP |
| MHI | 1.00 | 0.89 | 0.85 | −0.82 | −0.87 | −0.82 | −0.77 |
| MFI | 0.89 | 1.00 | 0.87 | −0.80 | −0.88 | −0.91 | −0.74 |
| SEA | 0.85 | 0.87 | 1.00 | −0.89 | −0.90 | −0.95 | −0.83 |
| SEP | −0.82 | −0.80 | −0.89 | 1.00 | 0.94 | 0.91 | 0.93 |
| SED | −0.87 | −0.88 | −0.90 | 0.94 | 1.00 | 0.92 | 0.94 |
| SES | −0.82 | −0.91 | −0.95 | 0.91 | 0.92 | 1.00 | 0.84 |
| DEP | −0.77 | −0.74 | −0.83 | 0.93 | 0.94 | 0.84 | 1.00 |

[a] Correlation matrix based on 788 census tracts; 5 out of 793 census tracts (0.63%) were omitted due to missing data.

[b] Correlation matrix based on 791 census tracts; 5 out of 796 census tracts (0.63%) were omitted due to missing data.

[c] Correlation matrix based on 821 census tracts; 4 out of 825 census tracts (0.48%) were omitted due to missing data.

[d] Correlation matrix based on 815 census tracts; 10 out of 825 census tracts (1.21%) were omitted due to missing data.

Abbreviations: MHI, Median Household Income; MFI, Median Family Income; SEA, Socioeconomic Advantage [20]; SEP, Socioeconomic Position [21]; SED, Socioeconomic Deprivation [22]; SES, Socioeconomic Status [23]; DEP, Deprivation [24].

Table S14. Relationships between simple and composite measures of neighborhood socioeconomic status in the State of Kansas.

| 2000[a] | MHI | MFI | SEA | SEP | SED | SES | DEP |
|---|---|---|---|---|---|---|---|
| MHI | 1.00 | 0.97 | 0.88 | −0.84 | −0.87 | −0.88 | −0.70 |
| MFI | 0.97 | 1.00 | 0.91 | −0.87 | −0.90 | −0.92 | −0.72 |
| SEA | 0.88 | 0.91 | 1.00 | −0.94 | −0.96 | −0.97 | −0.87 |
| SEP | −0.84 | −0.87 | −0.94 | 1.00 | 0.95 | 0.96 | 0.92 |
| SED | −0.87 | −0.90 | −0.96 | 0.95 | 1.00 | 0.95 | 0.92 |
| SES | −0.88 | −0.92 | −0.97 | 0.96 | 0.95 | 1.00 | 0.87 |
| DEP | −0.70 | −0.72 | −0.87 | 0.92 | 0.92 | 0.87 | 1.00 |
| 2005–2009[b] | MHI | MFI | SEA | SEP | SED | SES | DEP |
| MHI | 1.00 | 0.94 | 0.89 | −0.84 | −0.88 | −0.87 | −0.72 |
| MFI | 0.94 | 1.00 | 0.91 | −0.86 | −0.90 | −0.92 | −0.73 |
| SEA | 0.89 | 0.91 | 1.00 | −0.93 | −0.94 | −0.97 | −0.85 |
| SEP | −0.84 | −0.86 | −0.93 | 1.00 | 0.95 | 0.96 | 0.92 |
| SED | −0.88 | −0.90 | −0.94 | 0.95 | 1.00 | 0.94 | 0.92 |
| SES | −0.87 | −0.92 | −0.97 | 0.96 | 0.94 | 1.00 | 0.85 |
| DEP | −0.72 | −0.73 | −0.85 | 0.92 | 0.92 | 0.85 | 1.00 |
| 2010–2014[c] | MHI | MFI | SEA | SEP | SED | SES | DEP |
| MHI | 1.00 | 0.95 | 0.88 | −0.85 | −0.87 | −0.86 | −0.74 |
| MFI | 0.95 | 1.00 | 0.92 | −0.88 | −0.90 | −0.93 | −0.76 |
| SEA | 0.88 | 0.92 | 1.00 | −0.94 | −0.95 | −0.97 | −0.86 |
| SEP | −0.85 | −0.88 | −0.94 | 1.00 | 0.96 | 0.96 | 0.92 |
| SED | −0.87 | −0.90 | −0.95 | 0.96 | 1.00 | 0.95 | 0.93 |
| SES | −0.86 | −0.93 | −0.97 | 0.96 | 0.95 | 1.00 | 0.86 |
| DEP | −0.74 | −0.76 | −0.86 | 0.92 | 0.93 | 0.86 | 1.00 |
| 2015–2019[d] | MHI | MFI | SEA | SEP | SED | SES | DEP |
| MHI | 1.00 | 0.95 | 0.87 | −0.84 | −0.88 | −0.86 | −0.75 |
| MFI | 0.95 | 1.00 | 0.91 | −0.85 | −0.91 | −0.92 | −0.76 |
| SEA | 0.87 | 0.91 | 1.00 | −0.92 | −0.95 | −0.97 | −0.87 |
| SEP | −0.84 | −0.85 | −0.92 | 1.00 | 0.94 | 0.95 | 0.92 |
| SED | −0.88 | −0.91 | −0.95 | 0.94 | 1.00 | 0.95 | 0.93 |
| SES | −0.86 | −0.92 | −0.97 | 0.95 | 0.95 | 1.00 | 0.86 |
| DEP | −0.75 | −0.76 | −0.87 | 0.92 | 0.93 | 0.86 | 1.00 |

[a] Correlation matrix based on 718 census tracts; 9 out of 727 census tracts (1.24%) were omitted due to missing data.

[b] Correlation matrix based on 714 census tracts; 13 out of 727 census tracts (1.79%) were omitted due to missing data.

[c] Correlation matrix based on 757 census tracts; 13 out of 770 census tracts (1.69%) were omitted due to missing data.

[d] Correlation matrix based on 752 census tracts; 18 out of 770 census tracts (2.34%) were omitted due to missing data.

Abbreviations: MHI, Median Household Income; MFI, Median Family Income; SEA, Socioeconomic Advantage [20]; SEP, Socioeconomic Position [21]; SED, Socioeconomic Deprivation [22]; SES, Socioeconomic Status [23]; DEP, Deprivation [24].

Table S15. Relationships between simple and composite measures of neighborhood socioeconomic status in the State of Kentucky.

| 2000[a] | MHI | MFI | SEA | SEP | SED | SES | DEP |
|---|---|---|---|---|---|---|---|
| MHI | 1.00 | 0.96 | 0.91 | −0.91 | −0.93 | −0.90 | −0.87 |
| MFI | 0.96 | 1.00 | 0.95 | −0.94 | −0.95 | −0.96 | −0.87 |
| SEA | 0.91 | 0.95 | 1.00 | −0.96 | −0.95 | −0.97 | −0.90 |
| SEP | −0.91 | −0.94 | −0.96 | 1.00 | 0.96 | 0.97 | 0.91 |
| SED | −0.93 | −0.95 | −0.95 | 0.96 | 1.00 | 0.95 | 0.94 |
| SES | −0.90 | −0.96 | −0.97 | 0.97 | 0.95 | 1.00 | 0.86 |
| DEP | −0.87 | −0.87 | −0.90 | 0.91 | 0.94 | 0.86 | 1.00 |
| 2005–2009[b] | MHI | MFI | SEA | SEP | SED | SES | DEP |
| MHI | 1.00 | 0.93 | 0.89 | −0.89 | −0.93 | −0.87 | −0.86 |
| MFI | 0.93 | 1.00 | 0.91 | −0.91 | −0.93 | −0.93 | −0.84 |
| SEA | 0.89 | 0.91 | 1.00 | −0.94 | −0.93 | −0.96 | −0.87 |
| SEP | −0.89 | −0.91 | −0.94 | 1.00 | 0.95 | 0.96 | 0.92 |
| SED | −0.93 | −0.93 | −0.93 | 0.95 | 1.00 | 0.93 | 0.94 |
| SES | −0.87 | −0.93 | −0.96 | 0.96 | 0.93 | 1.00 | 0.85 |
| DEP | −0.86 | −0.84 | −0.87 | 0.92 | 0.94 | 0.85 | 1.00 |
| 2010–2014[c] | MHI | MFI | SEA | SEP | SED | SES | DEP |
| MHI | 1.00 | 0.94 | 0.89 | −0.90 | −0.92 | −0.88 | −0.87 |
| MFI | 0.94 | 1.00 | 0.92 | −0.92 | −0.94 | −0.93 | −0.87 |
| SEA | 0.89 | 0.92 | 1.00 | −0.95 | −0.94 | −0.96 | −0.88 |
| SEP | −0.90 | −0.92 | −0.95 | 1.00 | 0.96 | 0.97 | 0.92 |
| SED | −0.92 | −0.94 | −0.94 | 0.96 | 1.00 | 0.95 | 0.96 |
| SES | −0.88 | −0.93 | −0.96 | 0.97 | 0.95 | 1.00 | 0.87 |
| DEP | −0.87 | −0.87 | −0.88 | 0.92 | 0.96 | 0.87 | 1.00 |
| 2015–2019[d] | MHI | MFI | SEA | SEP | SED | SES | DEP |
| MHI | 1.00 | 0.92 | 0.89 | −0.89 | −0.91 | −0.87 | −0.85 |
| MFI | 0.92 | 1.00 | 0.92 | −0.90 | −0.93 | −0.94 | −0.83 |
| SEA | 0.89 | 0.92 | 1.00 | −0.94 | −0.93 | −0.96 | −0.87 |
| SEP | −0.89 | −0.90 | −0.94 | 1.00 | 0.96 | 0.95 | 0.93 |
| SED | −0.91 | −0.93 | −0.93 | 0.96 | 1.00 | 0.95 | 0.95 |
| SES | −0.87 | −0.94 | −0.96 | 0.95 | 0.95 | 1.00 | 0.86 |
| DEP | −0.85 | −0.83 | −0.87 | 0.93 | 0.95 | 0.86 | 1.00 |

[a] Correlation matrix based on 992 census tracts; 2 out of 994 census tracts (0.20%) were omitted due to missing data.

[b] Correlation matrix based on 988 census tracts; 6 out of 994 census tracts (0.60%) were omitted due to missing data.

[c] Correlation matrix based on 1,096 census tracts; 19 out of 1,115 census tracts (1.70%) were omitted due to missing data.

[d] Correlation matrix based on 1,093 census tracts; 22 out of 1,115 census tracts (1.97%) were omitted due to missing data.

Abbreviations: MHI, Median Household Income; MFI, Median Family Income; SEA, Socioeconomic Advantage [20]; SEP, Socioeconomic Position [21]; SED, Socioeconomic Deprivation [22]; SES, Socioeconomic Status [23]; DEP, Deprivation [24].

Table S16. Relationships between simple and composite measures of neighborhood socioeconomic status in the State of Louisiana.

| 2000[a] | MHI | MFI | SEA | SEP | SED | SES | DEP |
|---|---|---|---|---|---|---|---|
| MHI | 1.00 | 0.88 | 0.86 | −0.87 | −0.88 | −0.84 | −0.81 |
| MFI | 0.88 | 1.00 | 0.91 | −0.89 | −0.90 | −0.93 | −0.80 |
| SEA | 0.86 | 0.91 | 1.00 | −0.94 | −0.94 | −0.97 | −0.87 |
| SEP | −0.87 | −0.89 | −0.94 | 1.00 | 0.95 | 0.96 | 0.91 |
| SED | −0.88 | −0.90 | −0.94 | 0.95 | 1.00 | 0.93 | 0.96 |
| SES | −0.84 | −0.93 | −0.97 | 0.96 | 0.93 | 1.00 | 0.84 |
| DEP | −0.81 | −0.80 | −0.87 | 0.91 | 0.96 | 0.84 | 1.00 |
| 2005–2009[b] | MHI | MFI | SEA | SEP | SED | SES | DEP |
| MHI | 1.00 | 0.87 | 0.86 | −0.87 | −0.89 | −0.83 | −0.85 |
| MFI | 0.87 | 1.00 | 0.86 | −0.88 | −0.91 | −0.91 | −0.80 |
| SEA | 0.86 | 0.86 | 1.00 | −0.93 | −0.92 | −0.96 | −0.84 |
| SEP | −0.87 | −0.88 | −0.93 | 1.00 | 0.95 | 0.96 | 0.91 |
| SED | −0.89 | −0.91 | −0.92 | 0.95 | 1.00 | 0.93 | 0.95 |
| SES | −0.83 | −0.91 | −0.96 | 0.96 | 0.93 | 1.00 | 0.83 |
| DEP | −0.85 | −0.80 | −0.84 | 0.91 | 0.95 | 0.83 | 1.00 |
| 2010–2014[c] | MHI | MFI | SEA | SEP | SED | SES | DEP |
| MHI | 1.00 | 0.90 | 0.87 | −0.89 | −0.91 | −0.84 | −0.88 |
| MFI | 0.90 | 1.00 | 0.91 | −0.91 | −0.93 | −0.92 | −0.84 |
| SEA | 0.87 | 0.91 | 1.00 | −0.94 | −0.94 | −0.96 | −0.86 |
| SEP | −0.89 | −0.91 | −0.94 | 1.00 | 0.97 | 0.97 | 0.93 |
| SED | −0.91 | −0.93 | −0.94 | 0.97 | 1.00 | 0.94 | 0.96 |
| SES | −0.84 | −0.92 | −0.96 | 0.97 | 0.94 | 1.00 | 0.85 |
| DEP | −0.88 | −0.84 | −0.86 | 0.93 | 0.96 | 0.85 | 1.00 |
| 2015–2019[d] | MHI | MFI | SEA | SEP | SED | SES | DEP |
| MHI | 1.00 | 0.92 | 0.88 | −0.89 | −0.92 | −0.85 | −0.86 |
| MFI | 0.92 | 1.00 | 0.92 | −0.90 | −0.94 | −0.92 | −0.84 |
| SEA | 0.88 | 0.92 | 1.00 | −0.93 | −0.93 | −0.96 | −0.86 |
| SEP | −0.89 | −0.90 | −0.93 | 1.00 | 0.97 | 0.95 | 0.93 |
| SED | −0.92 | −0.94 | −0.93 | 0.97 | 1.00 | 0.93 | 0.95 |
| SES | −0.85 | −0.92 | −0.96 | 0.95 | 0.93 | 1.00 | 0.83 |
| DEP | −0.86 | −0.84 | −0.86 | 0.93 | 0.95 | 0.83 | 1.00 |

[a] Correlation matrix based on 1,099 census tracts; 7 out of 1,106 census tracts (0.63%) were omitted due to missing data.

[b] Correlation matrix based on 1,086 census tracts; 20 out of 1,106 census tracts (1.81%) were omitted due to missing data.

[c] Correlation matrix based on 1,111 census tracts; 37 out of 1,148 census tracts (3.22%) were omitted due to missing data.

[d] Correlation matrix based on 1,097 census tracts; 51 out of 1,148 census tracts (4.44%) were omitted due to missing data.

Abbreviations: MHI, Median Household Income; MFI, Median Family Income; SEA, Socioeconomic Advantage [20]; SEP, Socioeconomic Position [21]; SED, Socioeconomic Deprivation [22]; SES, Socioeconomic Status [23]; DEP, Deprivation [24].

Table S17. Relationships between simple and composite measures of neighborhood socioeconomic status in the State of Maine.

| 2000[a] | MHI | MFI | SEA | SEP | SED | SES | DEP |
|---|---|---|---|---|---|---|---|
| MHI | 1.00 | 0.92 | 0.86 | −0.87 | −0.93 | −0.84 | −0.88 |
| MFI | 0.92 | 1.00 | 0.91 | −0.90 | −0.94 | −0.92 | −0.86 |
| SEA | 0.86 | 0.91 | 1.00 | −0.94 | −0.92 | −0.98 | −0.84 |
| SEP | −0.87 | −0.90 | −0.94 | 1.00 | 0.95 | 0.96 | 0.93 |
| SED | −0.93 | −0.94 | −0.92 | 0.95 | 1.00 | 0.93 | 0.95 |
| SES | −0.84 | −0.92 | −0.98 | 0.96 | 0.93 | 1.00 | 0.85 |
| DEP | −0.88 | −0.86 | −0.84 | 0.93 | 0.95 | 0.85 | 1.00 |
| 2005–2009[b] | MHI | MFI | SEA | SEP | SED | SES | DEP |
| MHI | 1.00 | 0.91 | 0.84 | −0.86 | −0.91 | −0.82 | −0.85 |
| MFI | 0.91 | 1.00 | 0.86 | −0.87 | −0.92 | −0.89 | −0.83 |
| SEA | 0.84 | 0.86 | 1.00 | −0.92 | −0.87 | −0.95 | −0.78 |
| SEP | −0.86 | −0.87 | −0.92 | 1.00 | 0.94 | 0.96 | 0.90 |
| SED | −0.91 | −0.92 | −0.87 | 0.94 | 1.00 | 0.90 | 0.94 |
| SES | −0.82 | −0.89 | −0.95 | 0.96 | 0.90 | 1.00 | 0.82 |
| DEP | −0.85 | −0.83 | −0.78 | 0.90 | 0.94 | 0.82 | 1.00 |
| 2010–2014[c] | MHI | MFI | SEA | SEP | SED | SES | DEP |
| MHI | 1.00 | 0.89 | 0.83 | −0.86 | −0.90 | −0.81 | −0.85 |
| MFI | 0.89 | 1.00 | 0.86 | −0.85 | −0.90 | −0.88 | −0.77 |
| SEA | 0.83 | 0.86 | 1.00 | −0.93 | −0.88 | −0.96 | −0.78 |
| SEP | −0.86 | −0.85 | −0.93 | 1.00 | 0.94 | 0.96 | 0.89 |
| SED | −0.90 | −0.90 | −0.88 | 0.94 | 1.00 | 0.89 | 0.93 |
| SES | −0.81 | −0.88 | −0.96 | 0.96 | 0.89 | 1.00 | 0.78 |
| DEP | −0.85 | −0.77 | −0.78 | 0.89 | 0.93 | 0.78 | 1.00 |
| 2015–2019[d] | MHI | MFI | SEA | SEP | SED | SES | DEP |
| MHI | 1.00 | 0.90 | 0.87 | −0.87 | −0.92 | −0.85 | −0.86 |
| MFI | 0.90 | 1.00 | 0.88 | −0.86 | −0.91 | −0.92 | −0.78 |
| SEA | 0.87 | 0.88 | 1.00 | −0.91 | −0.89 | −0.95 | −0.80 |
| SEP | −0.87 | −0.86 | −0.91 | 1.00 | 0.94 | 0.94 | 0.91 |
| SED | −0.92 | −0.91 | −0.89 | 0.94 | 1.00 | 0.91 | 0.93 |
| SES | −0.85 | −0.92 | −0.95 | 0.94 | 0.91 | 1.00 | 0.81 |
| DEP | −0.86 | −0.78 | −0.80 | 0.91 | 0.93 | 0.81 | 1.00 |

[a] Correlation matrix based on 344 census tracts; 4 out of 348 census tracts (1.15%) were omitted due to missing data.

[b] Correlation matrix based on 342 census tracts; 6 out of 348 census tracts (1.72%) were omitted due to missing data.

[c] Correlation matrix based on 350 census tracts; 8 out of 358 census tracts (2.23%) were omitted due to missing data.

[d] Correlation matrix based on 350 census tracts; 8 out of 358 census tracts (2.23%) were omitted due to missing data.

Abbreviations: MHI, Median Household Income; MFI, Median Family Income; SEA, Socioeconomic Advantage [20]; SEP, Socioeconomic Position [21]; SED, Socioeconomic Deprivation [22]; SES, Socioeconomic Status [23]; DEP, Deprivation [24].

Table S18. Relationships between simple and composite measures of neighborhood socioeconomic status in the State of Maryland.

| 2000[a] | MHI | MFI | SEA | SEP | SED | SES | DEP |
|---|---|---|---|---|---|---|---|
| MHI | 1.00 | 0.97 | 0.90 | −0.88 | −0.88 | −0.90 | −0.75 |
| MFI | 0.97 | 1.00 | 0.93 | −0.90 | −0.89 | −0.94 | −0.76 |
| SEA | 0.90 | 0.93 | 1.00 | −0.94 | −0.93 | −0.97 | −0.87 |
| SEP | −0.88 | −0.90 | −0.94 | 1.00 | 0.96 | 0.97 | 0.91 |
| SED | −0.88 | −0.89 | −0.93 | 0.96 | 1.00 | 0.94 | 0.96 |
| SES | −0.90 | −0.94 | −0.97 | 0.97 | 0.94 | 1.00 | 0.86 |
| DEP | −0.75 | −0.76 | −0.87 | 0.91 | 0.96 | 0.86 | 1.00 |
| 2005–2009[b] | MHI | MFI | SEA | SEP | SED | SES | DEP |
| MHI | 1.00 | 0.95 | 0.90 | −0.90 | −0.89 | −0.89 | −0.78 |
| MFI | 0.95 | 1.00 | 0.92 | −0.92 | −0.90 | −0.93 | −0.79 |
| SEA | 0.90 | 0.92 | 1.00 | −0.95 | −0.93 | −0.97 | −0.88 |
| SEP | −0.90 | −0.92 | −0.95 | 1.00 | 0.96 | 0.98 | 0.92 |
| SED | −0.89 | −0.90 | −0.93 | 0.96 | 1.00 | 0.94 | 0.96 |
| SES | −0.89 | −0.93 | −0.97 | 0.98 | 0.94 | 1.00 | 0.89 |
| DEP | −0.78 | −0.79 | −0.88 | 0.92 | 0.96 | 0.89 | 1.00 |
| 2010–2014[c] | MHI | MFI | SEA | SEP | SED | SES | DEP |
| MHI | 1.00 | 0.95 | 0.89 | −0.89 | −0.88 | −0.88 | −0.76 |
| MFI | 0.95 | 1.00 | 0.92 | −0.92 | −0.90 | −0.93 | −0.78 |
| SEA | 0.89 | 0.92 | 1.00 | −0.95 | −0.92 | −0.97 | −0.86 |
| SEP | −0.89 | −0.92 | −0.95 | 1.00 | 0.96 | 0.98 | 0.92 |
| SED | −0.88 | −0.90 | −0.92 | 0.96 | 1.00 | 0.94 | 0.96 |
| SES | −0.88 | −0.93 | −0.97 | 0.98 | 0.94 | 1.00 | 0.88 |
| DEP | −0.76 | −0.78 | −0.86 | 0.92 | 0.96 | 0.88 | 1.00 |
| 2015–2019[d] | MHI | MFI | SEA | SEP | SED | SES | DEP |
| MHI | 1.00 | 0.95 | 0.91 | −0.87 | −0.89 | −0.89 | −0.76 |
| MFI | 0.95 | 1.00 | 0.93 | −0.89 | −0.91 | −0.93 | −0.78 |
| SEA | 0.91 | 0.93 | 1.00 | −0.93 | −0.92 | −0.97 | −0.84 |
| SEP | −0.87 | −0.89 | −0.93 | 1.00 | 0.95 | 0.97 | 0.92 |
| SED | −0.89 | −0.91 | −0.92 | 0.95 | 1.00 | 0.93 | 0.95 |
| SES | −0.89 | −0.93 | −0.97 | 0.97 | 0.93 | 1.00 | 0.86 |
| DEP | −0.76 | −0.78 | −0.84 | 0.92 | 0.95 | 0.86 | 1.00 |

[a] Correlation matrix based on 1,204 census tracts; 14 out of 1,218 census tracts (1.15%) were omitted due to missing data.

[b] Correlation matrix based on 1,194 census tracts; 25 out of 1,219 census tracts (2.05%) were omitted due to missing data.

[c] Correlation matrix based on 1,368 census tracts; 38 out of 1,406 census tracts (2.70%) were omitted due to missing data.

[d] Correlation matrix based on 1,364 census tracts; 42 out of 1,406 census tracts (2.99%) were omitted due to missing data.

Abbreviations: MHI, Median Household Income; MFI, Median Family Income; SEA, Socioeconomic Advantage [20]; SEP, Socioeconomic Position [21]; SED, Socioeconomic Deprivation [22]; SES, Socioeconomic Status [23]; DEP, Deprivation [24].

Table S19. Relationships between simple and composite measures of neighborhood socioeconomic status in the State of Massachusetts.

| 2000[a] | MHI | MFI | SEA | SEP | SED | SES | DEP |
|---|---|---|---|---|---|---|---|
| MHI | 1.00 | 0.95 | 0.89 | −0.89 | −0.87 | −0.89 | −0.79 |
| MFI | 0.95 | 1.00 | 0.91 | −0.91 | −0.87 | −0.93 | −0.79 |
| SEA | 0.89 | 0.91 | 1.00 | −0.96 | −0.91 | −0.98 | −0.87 |
| SEP | −0.89 | −0.91 | −0.96 | 1.00 | 0.94 | 0.97 | 0.91 |
| SED | −0.87 | −0.87 | −0.91 | 0.94 | 1.00 | 0.90 | 0.98 |
| SES | −0.89 | −0.93 | −0.98 | 0.97 | 0.90 | 1.00 | 0.84 |
| DEP | −0.79 | −0.79 | −0.87 | 0.91 | 0.98 | 0.84 | 1.00 |
| 2005–2009[b] | MHI | MFI | SEA | SEP | SED | SES | DEP |
| MHI | 1.00 | 0.92 | 0.88 | −0.88 | −0.86 | −0.86 | −0.78 |
| MFI | 0.92 | 1.00 | 0.90 | −0.90 | −0.87 | −0.92 | −0.78 |
| SEA | 0.88 | 0.90 | 1.00 | −0.95 | −0.89 | −0.97 | −0.83 |
| SEP | −0.88 | −0.90 | −0.95 | 1.00 | 0.94 | 0.98 | 0.91 |
| SED | −0.86 | −0.87 | −0.89 | 0.94 | 1.00 | 0.89 | 0.96 |
| SES | −0.86 | −0.92 | −0.97 | 0.98 | 0.89 | 1.00 | 0.84 |
| DEP | −0.78 | −0.78 | −0.83 | 0.91 | 0.96 | 0.84 | 1.00 |
| 2010–2014[c] | MHI | MFI | SEA | SEP | SED | SES | DEP |
| MHI | 1.00 | 0.92 | 0.88 | −0.89 | −0.86 | −0.86 | −0.78 |
| MFI | 0.92 | 1.00 | 0.90 | −0.91 | −0.88 | −0.92 | −0.78 |
| SEA | 0.88 | 0.90 | 1.00 | −0.96 | −0.89 | −0.97 | −0.84 |
| SEP | −0.89 | −0.91 | −0.96 | 1.00 | 0.95 | 0.98 | 0.92 |
| SED | −0.86 | −0.88 | −0.89 | 0.95 | 1.00 | 0.91 | 0.97 |
| SES | −0.86 | −0.92 | −0.97 | 0.98 | 0.91 | 1.00 | 0.87 |
| DEP | −0.78 | −0.78 | −0.84 | 0.92 | 0.97 | 0.87 | 1.00 |
| 2015–2019[d] | MHI | MFI | SEA | SEP | SED | SES | DEP |
| MHI | 1.00 | 0.93 | 0.89 | −0.88 | −0.86 | −0.87 | −0.77 |
| MFI | 0.93 | 1.00 | 0.90 | −0.90 | −0.88 | −0.92 | −0.77 |
| SEA | 0.89 | 0.90 | 1.00 | −0.93 | −0.87 | −0.96 | −0.81 |
| SEP | −0.88 | −0.90 | −0.93 | 1.00 | 0.94 | 0.97 | 0.91 |
| SED | −0.86 | −0.88 | −0.87 | 0.94 | 1.00 | 0.89 | 0.97 |
| SES | −0.87 | −0.92 | −0.96 | 0.97 | 0.89 | 1.00 | 0.84 |
| DEP | −0.77 | −0.77 | −0.81 | 0.91 | 0.97 | 0.84 | 1.00 |

[a] Correlation matrix based on 1,352 census tracts; 15 out of 1,367 census tracts (1.10%) were omitted due to missing data.

[b] Correlation matrix based on 1,335 census tracts; 31 out of 1,366 census tracts (2.27%) were omitted due to missing data.

[c] Correlation matrix based on 1,438 census tracts; 40 out of 1,478 census tracts (2.71%) were omitted due to missing data.

[d] Correlation matrix based on 1,430 census tracts; 48 out of 1,478 census tracts (3.25%) were omitted due to missing data.

Abbreviations: MHI, Median Household Income; MFI, Median Family Income; SEA, Socioeconomic Advantage [20]; SEP, Socioeconomic Position [21]; SED, Socioeconomic Deprivation [22]; SES, Socioeconomic Status [23]; DEP, Deprivation [24].

Table S20. Relationships between simple and composite measures of neighborhood socioeconomic status in the State of Michigan.

| 2000[a] | MHI | MFI | SEA | SEP | SED | SES | DEP |
|---|---|---|---|---|---|---|---|
| MHI | 1.00 | 0.96 | 0.88 | −0.87 | −0.87 | −0.88 | −0.75 |
| MFI | 0.96 | 1.00 | 0.91 | −0.90 | −0.89 | −0.93 | −0.77 |
| SEA | 0.88 | 0.91 | 1.00 | −0.95 | −0.95 | −0.97 | −0.89 |
| SEP | −0.87 | −0.90 | −0.95 | 1.00 | 0.96 | 0.97 | 0.91 |
| SED | −0.87 | −0.89 | −0.95 | 0.96 | 1.00 | 0.94 | 0.96 |
| SES | −0.88 | −0.93 | −0.97 | 0.97 | 0.94 | 1.00 | 0.86 |
| DEP | −0.75 | −0.77 | −0.89 | 0.91 | 0.96 | 0.86 | 1.00 |
| 2005–2009[b] | MHI | MFI | SEA | SEP | SED | SES | DEP |
| MHI | 1.00 | 0.93 | 0.89 | −0.87 | −0.89 | −0.86 | −0.77 |
| MFI | 0.93 | 1.00 | 0.91 | −0.89 | −0.92 | −0.93 | −0.78 |
| SEA | 0.89 | 0.91 | 1.00 | −0.95 | −0.93 | −0.96 | −0.87 |
| SEP | −0.87 | −0.89 | −0.95 | 1.00 | 0.95 | 0.97 | 0.91 |
| SED | −0.89 | −0.92 | −0.93 | 0.95 | 1.00 | 0.93 | 0.95 |
| SES | −0.86 | −0.93 | −0.96 | 0.97 | 0.93 | 1.00 | 0.86 |
| DEP | −0.77 | −0.78 | −0.87 | 0.91 | 0.95 | 0.86 | 1.00 |
| 2010–2014[c] | MHI | MFI | SEA | SEP | SED | SES | DEP |
| MHI | 1.00 | 0.94 | 0.89 | −0.88 | −0.90 | −0.88 | −0.81 |
| MFI | 0.94 | 1.00 | 0.91 | −0.91 | −0.93 | −0.93 | −0.82 |
| SEA | 0.89 | 0.91 | 1.00 | −0.95 | −0.94 | −0.97 | −0.90 |
| SEP | −0.88 | −0.91 | −0.95 | 1.00 | 0.96 | 0.97 | 0.93 |
| SED | −0.90 | −0.93 | −0.94 | 0.96 | 1.00 | 0.95 | 0.96 |
| SES | −0.88 | −0.93 | −0.97 | 0.97 | 0.95 | 1.00 | 0.89 |
| DEP | −0.81 | −0.82 | −0.90 | 0.93 | 0.96 | 0.89 | 1.00 |
| 2015–2019[d] | MHI | MFI | SEA | SEP | SED | SES | DEP |
| MHI | 1.00 | 0.94 | 0.89 | −0.88 | −0.90 | −0.89 | −0.79 |
| MFI | 0.94 | 1.00 | 0.92 | −0.89 | −0.91 | −0.94 | −0.79 |
| SEA | 0.89 | 0.92 | 1.00 | −0.94 | −0.94 | −0.97 | −0.88 |
| SEP | −0.88 | −0.89 | −0.94 | 1.00 | 0.96 | 0.96 | 0.92 |
| SED | −0.90 | −0.91 | −0.94 | 0.96 | 1.00 | 0.95 | 0.96 |
| SES | −0.89 | −0.94 | −0.97 | 0.96 | 0.95 | 1.00 | 0.87 |
| DEP | −0.79 | −0.79 | −0.88 | 0.92 | 0.96 | 0.87 | 1.00 |

[a] Correlation matrix based on 2,683 census tracts; 74 out of 2,757 census tracts (2.68%) were omitted due to missing data.

[b] Correlation matrix based on 2,674 census tracts; 83 out of 2,757 census tracts (3.01%) were omitted due to missing data.

[c] Correlation matrix based on 2,716 census tracts; 97 out of 2,813 census tracts (3.45%) were omitted due to missing data.

[d] Correlation matrix based on 2,684 census tracts; 129 out of 2,813 census tracts (4.59%) were omitted due to missing data.

Abbreviations: MHI, Median Household Income; MFI, Median Family Income; SEA, Socioeconomic Advantage [20]; SEP, Socioeconomic Position [21]; SED, Socioeconomic Deprivation [22]; SES, Socioeconomic Status [23]; DEP, Deprivation [24].

Table S21. Relationships between simple and composite measures of neighborhood socioeconomic status in the State of Minnesota.

| 2000[a] | MHI | MFI | SEA | SEP | SED | SES | DEP |
|---|---|---|---|---|---|---|---|
| MHI | 1.00 | 0.94 | 0.88 | −0.87 | −0.88 | −0.88 | −0.70 |
| MFI | 0.94 | 1.00 | 0.93 | −0.90 | −0.91 | −0.94 | −0.73 |
| SEA | 0.88 | 0.93 | 1.00 | −0.92 | −0.92 | −0.96 | −0.81 |
| SEP | −0.87 | −0.90 | −0.92 | 1.00 | 0.94 | 0.96 | 0.86 |
| SED | −0.88 | −0.91 | −0.92 | 0.94 | 1.00 | 0.93 | 0.91 |
| SES | −0.88 | −0.94 | −0.96 | 0.96 | 0.93 | 1.00 | 0.79 |
| DEP | −0.70 | −0.73 | −0.81 | 0.86 | 0.91 | 0.79 | 1.00 |
| 2005–2009[b] | MHI | MFI | SEA | SEP | SED | SES | DEP |
| MHI | 1.00 | 0.88 | 0.87 | −0.86 | −0.86 | −0.84 | −0.70 |
| MFI | 0.88 | 1.00 | 0.89 | −0.87 | −0.86 | −0.93 | −0.69 |
| SEA | 0.87 | 0.89 | 1.00 | −0.91 | −0.87 | −0.95 | −0.75 |
| SEP | −0.86 | −0.87 | −0.91 | 1.00 | 0.94 | 0.95 | 0.89 |
| SED | −0.86 | −0.86 | −0.87 | 0.94 | 1.00 | 0.90 | 0.92 |
| SES | −0.84 | −0.93 | −0.95 | 0.95 | 0.90 | 1.00 | 0.78 |
| DEP | −0.70 | −0.69 | −0.75 | 0.89 | 0.92 | 0.78 | 1.00 |
| 2010–2014[c] | MHI | MFI | SEA | SEP | SED | SES | DEP |
| MHI | 1.00 | 0.92 | 0.87 | −0.86 | −0.85 | −0.86 | −0.71 |
| MFI | 0.92 | 1.00 | 0.91 | −0.89 | −0.87 | −0.93 | −0.72 |
| SEA | 0.87 | 0.91 | 1.00 | −0.92 | −0.86 | −0.96 | −0.76 |
| SEP | −0.86 | −0.89 | −0.92 | 1.00 | 0.94 | 0.96 | 0.90 |
| SED | −0.85 | −0.87 | −0.86 | 0.94 | 1.00 | 0.90 | 0.94 |
| SES | −0.86 | −0.93 | −0.96 | 0.96 | 0.90 | 1.00 | 0.80 |
| DEP | −0.71 | −0.72 | −0.76 | 0.90 | 0.94 | 0.80 | 1.00 |
| 2015–2019[d] | MHI | MFI | SEA | SEP | SED | SES | DEP |
| MHI | 1.00 | 0.91 | 0.86 | −0.84 | −0.84 | −0.85 | −0.69 |
| MFI | 0.91 | 1.00 | 0.91 | −0.86 | −0.86 | −0.94 | −0.70 |
| SEA | 0.86 | 0.91 | 1.00 | −0.89 | −0.86 | −0.96 | −0.75 |
| SEP | −0.84 | −0.86 | −0.89 | 1.00 | 0.94 | 0.93 | 0.89 |
| SED | −0.84 | −0.86 | −0.86 | 0.94 | 1.00 | 0.89 | 0.93 |
| SES | −0.85 | −0.94 | −0.96 | 0.93 | 0.89 | 1.00 | 0.79 |
| DEP | −0.69 | −0.70 | −0.75 | 0.89 | 0.93 | 0.79 | 1.00 |

[a] Correlation matrix based on 1,291 census tracts; 12 out of 1,303 census tracts (0.92%) were omitted due to missing data.

[b] Correlation matrix based on 1,291 census tracts; 12 out of 1,303 census tracts (0.92%) were omitted due to missing data.

[c] Correlation matrix based on 1,330 census tracts; 8 out of 1,338 census tracts (0.60%) were omitted due to missing data.

[d] Correlation matrix based on 1,326 census tracts; 12 out of 1,338 census tracts (0.90%) were omitted due to missing data.

Abbreviations: MHI, Median Household Income; MFI, Median Family Income; SEA, Socioeconomic Advantage [20]; SEP, Socioeconomic Position [21]; SED, Socioeconomic Deprivation [22]; SES, Socioeconomic Status [23]; DEP, Deprivation [24].

Table S22. Relationships between simple and composite measures of neighborhood socioeconomic status in the State of Mississippi.

| 2000[a] | MHI | MFI | SEA | SEP | SED | SES | DEP |
|---|---|---|---|---|---|---|---|
| MHI | 1.00 | 0.95 | 0.89 | −0.87 | −0.90 | −0.90 | −0.83 |
| MFI | 0.95 | 1.00 | 0.90 | −0.88 | −0.91 | −0.92 | −0.84 |
| SEA | 0.89 | 0.90 | 1.00 | −0.93 | −0.94 | −0.97 | −0.87 |
| SEP | −0.87 | −0.88 | −0.93 | 1.00 | 0.95 | 0.96 | 0.91 |
| SED | −0.90 | −0.91 | −0.94 | 0.95 | 1.00 | 0.95 | 0.95 |
| SES | −0.90 | −0.92 | −0.97 | 0.96 | 0.95 | 1.00 | 0.86 |
| DEP | −0.83 | −0.84 | −0.87 | 0.91 | 0.95 | 0.86 | 1.00 |
| 2005–2009[b] | MHI | MFI | SEA | SEP | SED | SES | DEP |
| MHI | 1.00 | 0.92 | 0.88 | −0.87 | −0.91 | −0.88 | −0.82 |
| MFI | 0.92 | 1.00 | 0.89 | −0.89 | −0.92 | −0.92 | −0.82 |
| SEA | 0.88 | 0.89 | 1.00 | −0.93 | −0.92 | −0.96 | −0.83 |
| SEP | −0.87 | −0.89 | −0.93 | 1.00 | 0.95 | 0.96 | 0.91 |
| SED | −0.91 | −0.92 | −0.92 | 0.95 | 1.00 | 0.94 | 0.95 |
| SES | −0.88 | −0.92 | −0.96 | 0.96 | 0.94 | 1.00 | 0.85 |
| DEP | −0.82 | −0.82 | −0.83 | 0.91 | 0.95 | 0.85 | 1.00 |
| 2010–2014[c] | MHI | MFI | SEA | SEP | SED | SES | DEP |
| MHI | 1.00 | 0.93 | 0.88 | −0.89 | −0.93 | −0.89 | −0.87 |
| MFI | 0.93 | 1.00 | 0.91 | −0.90 | −0.93 | −0.93 | −0.85 |
| SEA | 0.88 | 0.91 | 1.00 | −0.92 | −0.92 | −0.96 | −0.85 |
| SEP | −0.89 | −0.90 | −0.92 | 1.00 | 0.95 | 0.96 | 0.91 |
| SED | −0.93 | −0.93 | −0.92 | 0.95 | 1.00 | 0.95 | 0.96 |
| SES | −0.89 | −0.93 | −0.96 | 0.96 | 0.95 | 1.00 | 0.86 |
| DEP | −0.87 | −0.85 | −0.85 | 0.91 | 0.96 | 0.86 | 1.00 |
| 2015–2019[d] | MHI | MFI | SEA | SEP | SED | SES | DEP |
| MHI | 1.00 | 0.93 | 0.86 | −0.87 | −0.92 | −0.87 | −0.85 |
| MFI | 0.93 | 1.00 | 0.89 | −0.88 | −0.93 | −0.92 | −0.84 |
| SEA | 0.86 | 0.89 | 1.00 | −0.91 | −0.92 | −0.96 | −0.83 |
| SEP | −0.87 | −0.88 | −0.91 | 1.00 | 0.94 | 0.95 | 0.91 |
| SED | −0.92 | −0.93 | −0.92 | 0.94 | 1.00 | 0.94 | 0.94 |
| SES | −0.87 | −0.92 | −0.96 | 0.95 | 0.94 | 1.00 | 0.85 |
| DEP | −0.85 | −0.84 | −0.83 | 0.91 | 0.94 | 0.85 | 1.00 |

[a] Correlation matrix based on 602 census tracts; 3 out of 605 census tracts (0.50%) were omitted due to missing data.

[b] Correlation matrix based on 600 census tracts; 5 out of 605 census tracts (0.83%) were omitted due to missing data.

[c] Correlation matrix based on 653 census tracts; 11 out of 664 census tracts (1.66%) were omitted due to missing data.

[d] Correlation matrix based on 647 census tracts; 17 out of 664 census tracts (2.56%) were omitted due to missing data.

Abbreviations: MHI, Median Household Income; MFI, Median Family Income; SEA, Socioeconomic Advantage [20]; SEP, Socioeconomic Position [21]; SED, Socioeconomic Deprivation [22]; SES, Socioeconomic Status [23]; DEP, Deprivation [24].

Table S23. Relationships between simple and composite measures of neighborhood socioeconomic status in the State of Missouri.

| 2000[a] | MHI | MFI | SEA | SEP | SED | SES | DEP |
|---|---|---|---|---|---|---|---|
| MHI | 1.00 | 0.96 | 0.88 | −0.89 | −0.91 | −0.88 | −0.76 |
| MFI | 0.96 | 1.00 | 0.91 | −0.92 | −0.92 | −0.94 | −0.76 |
| SEA | 0.88 | 0.91 | 1.00 | −0.95 | −0.94 | −0.97 | −0.86 |
| SEP | −0.89 | −0.92 | −0.95 | 1.00 | 0.96 | 0.96 | 0.89 |
| SED | −0.91 | −0.92 | −0.94 | 0.96 | 1.00 | 0.94 | 0.93 |
| SES | −0.88 | −0.94 | −0.97 | 0.96 | 0.94 | 1.00 | 0.82 |
| DEP | −0.76 | −0.76 | −0.86 | 0.89 | 0.93 | 0.82 | 1.00 |
| 2005–2009[b] | MHI | MFI | SEA | SEP | SED | SES | DEP |
| MHI | 1.00 | 0.93 | 0.88 | −0.88 | −0.90 | −0.86 | −0.78 |
| MFI | 0.93 | 1.00 | 0.90 | −0.89 | −0.92 | −0.93 | −0.78 |
| SEA | 0.88 | 0.90 | 1.00 | −0.95 | −0.93 | −0.97 | −0.86 |
| SEP | −0.88 | −0.89 | −0.95 | 1.00 | 0.96 | 0.97 | 0.91 |
| SED | −0.90 | −0.92 | −0.93 | 0.96 | 1.00 | 0.94 | 0.94 |
| SES | −0.86 | −0.93 | −0.97 | 0.97 | 0.94 | 1.00 | 0.85 |
| DEP | −0.78 | −0.78 | −0.86 | 0.91 | 0.94 | 0.85 | 1.00 |
| 2010–2014[c] | MHI | MFI | SEA | SEP | SED | SES | DEP |
| MHI | 1.00 | 0.93 | 0.88 | −0.88 | −0.89 | −0.87 | −0.80 |
| MFI | 0.93 | 1.00 | 0.92 | −0.91 | −0.92 | −0.93 | −0.81 |
| SEA | 0.88 | 0.92 | 1.00 | −0.95 | −0.93 | −0.96 | −0.88 |
| SEP | −0.88 | −0.91 | −0.95 | 1.00 | 0.95 | 0.97 | 0.92 |
| SED | −0.89 | −0.92 | −0.93 | 0.95 | 1.00 | 0.94 | 0.95 |
| SES | −0.87 | −0.93 | −0.96 | 0.97 | 0.94 | 1.00 | 0.87 |
| DEP | −0.80 | −0.81 | −0.88 | 0.92 | 0.95 | 0.87 | 1.00 |
| 2015–2019[d] | MHI | MFI | SEA | SEP | SED | SES | DEP |
| MHI | 1.00 | 0.93 | 0.88 | −0.88 | −0.91 | −0.87 | −0.83 |
| MFI | 0.93 | 1.00 | 0.91 | −0.89 | −0.92 | −0.93 | −0.81 |
| SEA | 0.88 | 0.91 | 1.00 | −0.93 | −0.94 | −0.96 | −0.89 |
| SEP | −0.88 | −0.89 | −0.93 | 1.00 | 0.95 | 0.95 | 0.92 |
| SED | −0.91 | −0.92 | −0.94 | 0.95 | 1.00 | 0.95 | 0.95 |
| SES | −0.87 | −0.93 | −0.96 | 0.95 | 0.95 | 1.00 | 0.87 |
| DEP | −0.83 | −0.81 | −0.89 | 0.92 | 0.95 | 0.87 | 1.00 |

[a] Correlation matrix based on 1,300 census tracts; 20 out of 1,320 census tracts (1.52%) were omitted due to missing data.

[b] Correlation matrix based on 1,295 census tracts; 25 out of 1,320 census tracts (1.89%) were omitted due to missing data.

[c] Correlation matrix based on 1,379 census tracts; 14 out of 1,393 census tracts (1.01%) were omitted due to missing data.

[d] Correlation matrix based on 1,373 census tracts; 20 out of 1,393 census tracts (1.44%) were omitted due to missing data.

Abbreviations: MHI, Median Household Income; MFI, Median Family Income; SEA, Socioeconomic Advantage [20]; SEP, Socioeconomic Position [21]; SED, Socioeconomic Deprivation [22]; SES, Socioeconomic Status [23]; DEP, Deprivation [24].

Table S24. Relationships between simple and composite measures of neighborhood socioeconomic status in the State of Montana.

| 2000[a] | MHI | MFI | SEA | SEP | SED | SES | DEP |
|---|---|---|---|---|---|---|---|
| MHI | 1.00 | 0.92 | 0.74 | −0.72 | −0.81 | 0.76 | −0.63 |
| MFI | 0.92 | 1.00 | 0.77 | −0.71 | −0.81 | 0.83 | −0.66 |
| SEA | 0.74 | 0.77 | 1.00 | −0.87 | −0.86 | 0.91 | −0.77 |
| SEP | −0.72 | −0.71 | −0.87 | 1.00 | 0.85 | −0.86 | 0.88 |
| SED | −0.81 | −0.81 | −0.86 | 0.85 | 1.00 | −0.91 | 0.88 |
| SES | 0.76 | 0.83 | 0.91 | −0.86 | −0.91 | 1.00 | −0.79 |
| DEP | −0.63 | −0.66 | −0.77 | 0.88 | 0.88 | −0.79 | 1.00 |
| 2005–2009[b] | MHI | MFI | SEA | SEP | SED | SES | DEP |
| MHI | 1.00 | 0.91 | 0.78 | −0.72 | −0.81 | −0.75 | −0.54 |
| MFI | 0.91 | 1.00 | 0.81 | −0.74 | −0.87 | −0.86 | −0.61 |
| SEA | 0.78 | 0.81 | 1.00 | −0.86 | −0.86 | −0.91 | −0.66 |
| SEP | −0.72 | −0.74 | −0.86 | 1.00 | 0.87 | 0.88 | 0.81 |
| SED | −0.81 | −0.87 | −0.86 | 0.87 | 1.00 | 0.90 | 0.84 |
| SES | −0.75 | −0.86 | −0.91 | 0.88 | 0.90 | 1.00 | 0.71 |
| DEP | −0.54 | −0.61 | −0.66 | 0.81 | 0.84 | 0.71 | 1.00 |
| 2010–2014[c] | MHI | MFI | SEA | SEP | SED | SES | DEP |
| MHI | 1.00 | 0.87 | 0.77 | −0.81 | −0.84 | −0.77 | −0.59 |
| MFI | 0.87 | 1.00 | 0.78 | −0.81 | −0.88 | −0.86 | −0.65 |
| SEA | 0.77 | 0.78 | 1.00 | −0.89 | −0.86 | −0.92 | −0.70 |
| SEP | −0.81 | −0.81 | −0.89 | 1.00 | 0.92 | 0.94 | 0.84 |
| SED | −0.84 | −0.88 | −0.86 | 0.92 | 1.00 | 0.91 | 0.86 |
| SES | −0.77 | −0.86 | −0.92 | 0.94 | 0.91 | 1.00 | 0.76 |
| DEP | −0.59 | −0.65 | −0.70 | 0.84 | 0.86 | 0.76 | 1.00 |
| 2015–2019[d] | MHI | MFI | SEA | SEP | SED | SES | DEP |
| MHI | 1.00 | 0.88 | 0.77 | −0.80 | −0.85 | −0.80 | −0.61 |
| MFI | 0.88 | 1.00 | 0.78 | −0.80 | −0.88 | −0.89 | −0.66 |
| SEA | 0.77 | 0.78 | 1.00 | −0.85 | −0.81 | −0.91 | −0.63 |
| SEP | −0.80 | −0.80 | −0.85 | 1.00 | 0.90 | 0.91 | 0.83 |
| SED | −0.85 | −0.88 | −0.81 | 0.90 | 1.00 | 0.90 | 0.85 |
| SES | −0.80 | −0.89 | −0.91 | 0.91 | 0.90 | 1.00 | 0.71 |
| DEP | −0.61 | −0.66 | −0.63 | 0.83 | 0.85 | 0.71 | 1.00 |

[a] Correlation matrix based on 268 census tracts; 2 out of 270 census tracts (0.74%) were omitted due to missing data.

[b] Correlation matrix based on 258 census tracts; 12 out of 270 census tracts (4.44%) were omitted due to missing data.

[c] Correlation matrix based on 267 census tracts; 4 out of 271 census tracts (1.48%) were omitted due to missing data.

[d] Correlation matrix based on 265 census tracts; 6 out of 271 census tracts (2.21%) were omitted due to missing data.

Abbreviations: MHI, Median Household Income; MFI, Median Family Income; SEA, Socioeconomic Advantage [20]; SEP, Socioeconomic Position [21]; SED, Socioeconomic Deprivation [22]; SES, Socioeconomic Status [23]; DEP, Deprivation [24].

Table S25. Relationships between simple and composite measures of neighborhood socioeconomic status in the State of Nebraska.

| 2000[a] | MHI | MFI | SEA | SEP | SED | SES | DEP |
|---|---|---|---|---|---|---|---|
| MHI | 1.00 | 0.95 | 0.87 | −0.81 | −0.87 | −0.85 | −0.64 |
| MFI | 0.95 | 1.00 | 0.88 | −0.82 | −0.88 | −0.88 | −0.65 |
| SEA | 0.87 | 0.88 | 1.00 | −0.93 | −0.93 | −0.95 | −0.81 |
| SEP | −0.81 | −0.82 | −0.93 | 1.00 | 0.94 | 0.97 | 0.87 |
| SED | −0.87 | −0.88 | −0.93 | 0.94 | 1.00 | 0.93 | 0.89 |
| SES | −0.85 | −0.88 | −0.95 | 0.97 | 0.93 | 1.00 | 0.80 |
| DEP | −0.64 | −0.65 | −0.81 | 0.87 | 0.89 | 0.80 | 1.00 |
| 2005–2009[b] | MHI | MFI | SEA | SEP | SED | SES | DEP |
| MHI | 1.00 | 0.92 | 0.86 | −0.82 | −0.87 | −0.84 | −0.64 |
| MFI | 0.92 | 1.00 | 0.88 | −0.83 | −0.90 | −0.89 | −0.66 |
| SEA | 0.86 | 0.88 | 1.00 | −0.93 | −0.90 | −0.96 | −0.76 |
| SEP | −0.82 | −0.83 | −0.93 | 1.00 | 0.93 | 0.96 | 0.89 |
| SED | −0.87 | −0.90 | −0.90 | 0.93 | 1.00 | 0.92 | 0.87 |
| SES | −0.84 | −0.89 | −0.96 | 0.96 | 0.92 | 1.00 | 0.78 |
| DEP | −0.64 | −0.66 | −0.76 | 0.89 | 0.87 | 0.78 | 1.00 |
| 2010–2014[c] | MHI | MFI | SEA | SEP | SED | SES | DEP |
| MHI | 1.00 | 0.94 | 0.87 | −0.84 | −0.88 | −0.86 | −0.69 |
| MFI | 0.94 | 1.00 | 0.91 | −0.87 | −0.91 | −0.93 | −0.72 |
| SEA | 0.87 | 0.91 | 1.00 | −0.93 | −0.93 | −0.96 | −0.82 |
| SEP | −0.84 | −0.87 | −0.93 | 1.00 | 0.95 | 0.95 | 0.89 |
| SED | −0.88 | −0.91 | −0.93 | 0.95 | 1.00 | 0.94 | 0.91 |
| SES | −0.86 | −0.93 | −0.96 | 0.95 | 0.94 | 1.00 | 0.82 |
| DEP | −0.69 | −0.72 | −0.82 | 0.89 | 0.91 | 0.82 | 1.00 |
| 2015–2019[d] | MHI | MFI | SEA | SEP | SED | SES | DEP |
| MHI | 1.00 | 0.94 | 0.88 | −0.82 | −0.86 | −0.87 | −0.68 |
| MFI | 0.94 | 1.00 | 0.91 | −0.83 | −0.89 | −0.92 | −0.71 |
| SEA | 0.88 | 0.91 | 1.00 | −0.92 | −0.94 | −0.97 | −0.83 |
| SEP | −0.82 | −0.83 | −0.92 | 1.00 | 0.94 | 0.94 | 0.91 |
| SED | −0.86 | −0.89 | −0.94 | 0.94 | 1.00 | 0.94 | 0.92 |
| SES | −0.87 | −0.92 | −0.97 | 0.94 | 0.94 | 1.00 | 0.84 |
| DEP | −0.68 | −0.71 | −0.83 | 0.91 | 0.92 | 0.84 | 1.00 |

[a] Correlation matrix based on 499 census tracts; 4 out of 503 census tracts (0.80%) were omitted due to missing data.

[b] Correlation matrix based on 496 census tracts; 7 out of 503 census tracts (1.39%) were omitted due to missing data.

[c] Correlation matrix based on 524 census tracts; 8 out of 532 census tracts (1.50%) were omitted due to missing data.

[d] Correlation matrix based on 523 census tracts; 9 out of 532 census tracts (1.69%) were omitted due to missing data.

Abbreviations: MHI, Median Household Income; MFI, Median Family Income; SEA, Socioeconomic Advantage [20]; SEP, Socioeconomic Position [21]; SED, Socioeconomic Deprivation [22]; SES, Socioeconomic Status [23]; DEP, Deprivation [24].

Table S26. Relationships between simple and composite measures of neighborhood socioeconomic status in the State of Nevada.

| 2000[a] | MHI | MFI | SEA | SEP | SED | SES | DEP |
|---|---|---|---|---|---|---|---|
| MHI | 1.00 | 0.96 | 0.87 | −0.86 | −0.88 | −0.87 | −0.75 |
| MFI | 0.96 | 1.00 | 0.90 | −0.88 | −0.89 | −0.92 | −0.76 |
| SEA | 0.87 | 0.90 | 1.00 | −0.93 | −0.93 | −0.96 | −0.86 |
| SEP | −0.86 | −0.88 | −0.93 | 1.00 | 0.95 | 0.97 | 0.92 |
| SED | −0.88 | −0.89 | −0.93 | 0.95 | 1.00 | 0.94 | 0.96 |
| SES | −0.87 | −0.92 | −0.96 | 0.97 | 0.94 | 1.00 | 0.87 |
| DEP | −0.75 | −0.76 | −0.86 | 0.92 | 0.96 | 0.87 | 1.00 |
| 2005–2009[b] | MHI | MFI | SEA | SEP | SED | SES | DEP |
| MHI | 1.00 | 0.92 | 0.84 | −0.87 | −0.89 | −0.85 | −0.76 |
| MFI | 0.92 | 1.00 | 0.86 | −0.89 | −0.92 | −0.91 | −0.79 |
| SEA | 0.84 | 0.86 | 1.00 | −0.92 | −0.91 | −0.95 | −0.87 |
| SEP | −0.87 | −0.89 | −0.92 | 1.00 | 0.94 | 0.98 | 0.91 |
| SED | −0.89 | −0.92 | −0.91 | 0.94 | 1.00 | 0.93 | 0.94 |
| SES | −0.85 | −0.91 | −0.95 | 0.98 | 0.93 | 1.00 | 0.90 |
| DEP | −0.76 | −0.79 | −0.87 | 0.91 | 0.94 | 0.90 | 1.00 |
| 2010–2014[c] | MHI | MFI | SEA | SEP | SED | SES | DEP |
| MHI | 1.00 | 0.92 | 0.86 | −0.88 | −0.90 | −0.86 | −0.80 |
| MFI | 0.92 | 1.00 | 0.88 | −0.89 | −0.89 | −0.91 | −0.80 |
| SEA | 0.86 | 0.88 | 1.00 | −0.94 | −0.93 | −0.95 | −0.88 |
| SEP | −0.88 | −0.89 | −0.94 | 1.00 | 0.96 | 0.98 | 0.93 |
| SED | −0.90 | −0.89 | −0.93 | 0.96 | 1.00 | 0.94 | 0.97 |
| SES | −0.86 | −0.91 | −0.95 | 0.98 | 0.94 | 1.00 | 0.89 |
| DEP | −0.80 | −0.80 | −0.88 | 0.93 | 0.97 | 0.89 | 1.00 |
| 2015–2019[d] | MHI | MFI | SEA | SEP | SED | SES | DEP |
| MHI | 1.00 | 0.93 | 0.87 | −0.87 | −0.89 | −0.87 | −0.79 |
| MFI | 0.93 | 1.00 | 0.90 | −0.89 | −0.90 | −0.92 | −0.80 |
| SEA | 0.87 | 0.90 | 1.00 | −0.92 | −0.93 | −0.95 | −0.87 |
| SEP | −0.87 | −0.89 | −0.92 | 1.00 | 0.95 | 0.97 | 0.92 |
| SED | −0.89 | −0.90 | −0.93 | 0.95 | 1.00 | 0.94 | 0.96 |
| SES | −0.87 | −0.92 | −0.95 | 0.97 | 0.94 | 1.00 | 0.89 |
| DEP | −0.79 | −0.80 | −0.87 | 0.92 | 0.96 | 0.89 | 1.00 |

[a] Correlation matrix based on 477 census tracts; 10 out of 487 census tracts (2.05%) were omitted due to missing data.

[b] Correlation matrix based on 474 census tracts; 13 out of 487 census tracts (2.67%) were omitted due to missing data.

[c] Correlation matrix based on 672 census tracts; 15 out of 687 census tracts (2.18%) were omitted due to missing data.

[d] Correlation matrix based on 666 census tracts; 21 out of 687 census tracts (3.06%) were omitted due to missing data.

Abbreviations: MHI, Median Household Income; MFI, Median Family Income; SEA, Socioeconomic Advantage [20]; SEP, Socioeconomic Position [21]; SED, Socioeconomic Deprivation [22]; SES, Socioeconomic Status [23]; DEP, Deprivation [24].

Table S27. Relationships between simple and composite measures of neighborhood socioeconomic status in the State of New Hampshire.

| 2000[a] | MHI | MFI | SEA | SEP | SED | SES | DEP |
|---|---|---|---|---|---|---|---|
| MHI | 1.00 | 0.95 | 0.88 | −0.85 | −0.91 | −0.87 | −0.85 |
| MFI | 0.95 | 1.00 | 0.93 | −0.85 | −0.91 | −0.94 | −0.82 |
| SEA | 0.88 | 0.93 | 1.00 | −0.90 | −0.90 | −0.98 | −0.84 |
| SEP | −0.85 | −0.85 | −0.90 | 1.00 | 0.93 | 0.92 | 0.91 |
| SED | −0.91 | −0.91 | −0.90 | 0.93 | 1.00 | 0.90 | 0.97 |
| SES | −0.87 | −0.94 | −0.98 | 0.92 | 0.90 | 1.00 | 0.82 |
| DEP | −0.85 | −0.82 | −0.84 | 0.91 | 0.97 | 0.82 | 1.00 |
| 2005–2009[b] | MHI | MFI | SEA | SEP | SED | SES | DEP |
| MHI | 1.00 | 0.90 | 0.86 | −0.82 | −0.89 | −0.83 | −0.79 |
| MFI | 0.90 | 1.00 | 0.88 | −0.82 | −0.86 | −0.91 | −0.75 |
| SEA | 0.86 | 0.88 | 1.00 | −0.91 | −0.86 | −0.96 | −0.78 |
| SEP | −0.82 | −0.82 | −0.91 | 1.00 | 0.92 | 0.94 | 0.91 |
| SED | −0.89 | −0.86 | −0.86 | 0.92 | 1.00 | 0.87 | 0.95 |
| SES | −0.83 | −0.91 | −0.96 | 0.94 | 0.87 | 1.00 | 0.82 |
| DEP | −0.79 | −0.75 | −0.78 | 0.91 | 0.95 | 0.82 | 1.00 |
| 2010–2014[c] | MHI | MFI | SEA | SEP | SED | SES | DEP |
| MHI | 1.00 | 0.90 | 0.85 | −0.84 | −0.87 | −0.83 | −0.79 |
| MFI | 0.90 | 1.00 | 0.87 | −0.84 | −0.88 | −0.91 | −0.76 |
| SEA | 0.85 | 0.87 | 1.00 | −0.91 | −0.87 | −0.95 | −0.80 |
| SEP | −0.84 | −0.84 | −0.91 | 1.00 | 0.95 | 0.96 | 0.93 |
| SED | −0.87 | −0.88 | −0.87 | 0.95 | 1.00 | 0.92 | 0.96 |
| SES | −0.83 | −0.91 | −0.95 | 0.96 | 0.92 | 1.00 | 0.86 |
| DEP | −0.79 | −0.76 | −0.80 | 0.93 | 0.96 | 0.86 | 1.00 |
| 2015–2019[d] | MHI | MFI | SEA | SEP | SED | SES | DEP |
| MHI | 1.00 | 0.87 | 0.87 | −0.83 | −0.87 | −0.83 | −0.80 |
| MFI | 0.87 | 1.00 | 0.88 | −0.80 | −0.85 | −0.92 | −0.75 |
| SEA | 0.87 | 0.88 | 1.00 | −0.91 | −0.89 | −0.95 | −0.84 |
| SEP | −0.83 | −0.80 | −0.91 | 1.00 | 0.93 | 0.94 | 0.93 |
| SED | −0.87 | −0.85 | −0.89 | 0.93 | 1.00 | 0.90 | 0.96 |
| SES | −0.83 | −0.92 | −0.95 | 0.94 | 0.90 | 1.00 | 0.86 |
| DEP | −0.80 | −0.75 | −0.84 | 0.93 | 0.96 | 0.86 | 1.00 |

[a] Correlation matrix based on 272 census tracts; 1 out of 273 census tracts (0.37%) were omitted due to missing data.

[b] Correlation matrix based on 272 census tracts; 1 out of 273 census tracts (0.37%) were omitted due to missing data.

[c] Correlation matrix based on 292 census tracts; 3 out of 295 census tracts (1.02%) were omitted due to missing data.

[d] Correlation matrix based on 292 census tracts; 3 out of 295 census tracts (1.02%) were omitted due to missing data.

Abbreviations: MHI, Median Household Income; MFI, Median Family Income; SEA, Socioeconomic Advantage [20]; SEP, Socioeconomic Position [21]; SED, Socioeconomic Deprivation [22]; SES, Socioeconomic Status [23]; DEP, Deprivation [24].

Table S28. Relationships between simple and composite measures of neighborhood socioeconomic status in the State of New Jersey.

| 2000[a] | MHI | MFI | SEA | SEP | SED | SES | DEP |
|---|---|---|---|---|---|---|---|
| MHI | 1.00 | 0.98 | 0.91 | −0.91 | −0.86 | −0.92 | −0.78 |
| MFI | 0.98 | 1.00 | 0.93 | −0.92 | −0.88 | −0.95 | −0.80 |
| SEA | 0.91 | 0.93 | 1.00 | −0.95 | −0.93 | −0.97 | −0.89 |
| SEP | −0.91 | −0.92 | −0.95 | 1.00 | 0.95 | 0.98 | 0.92 |
| SED | −0.86 | −0.88 | −0.93 | 0.95 | 1.00 | 0.93 | 0.98 |
| SES | −0.92 | −0.95 | −0.97 | 0.98 | 0.93 | 1.00 | 0.88 |
| DEP | −0.78 | −0.80 | −0.89 | 0.92 | 0.98 | 0.88 | 1.00 |
| 2005–2009[b] | MHI | MFI | SEA | SEP | SED | SES | DEP |
| MHI | 1.00 | 0.96 | 0.90 | −0.90 | −0.87 | −0.90 | −0.79 |
| MFI | 0.96 | 1.00 | 0.91 | −0.92 | −0.89 | −0.93 | −0.80 |
| SEA | 0.90 | 0.91 | 1.00 | −0.95 | −0.91 | −0.97 | −0.87 |
| SEP | −0.90 | −0.92 | −0.95 | 1.00 | 0.95 | 0.98 | 0.92 |
| SED | −0.87 | −0.89 | −0.91 | 0.95 | 1.00 | 0.91 | 0.97 |
| SES | −0.90 | −0.93 | −0.97 | 0.98 | 0.91 | 1.00 | 0.87 |
| DEP | −0.79 | −0.80 | −0.87 | 0.92 | 0.97 | 0.87 | 1.00 |
| 2010–2014[c] | MHI | MFI | SEA | SEP | SED | SES | DEP |
| MHI | 1.00 | 0.96 | 0.91 | −0.90 | −0.87 | −0.90 | −0.78 |
| MFI | 0.96 | 1.00 | 0.92 | −0.93 | −0.89 | −0.94 | −0.80 |
| SEA | 0.91 | 0.92 | 1.00 | −0.96 | −0.91 | −0.97 | −0.86 |
| SEP | −0.90 | −0.93 | −0.96 | 1.00 | 0.95 | 0.98 | 0.92 |
| SED | −0.87 | −0.89 | −0.91 | 0.95 | 1.00 | 0.92 | 0.97 |
| SES | −0.90 | −0.94 | −0.97 | 0.98 | 0.92 | 1.00 | 0.86 |
| DEP | −0.78 | −0.80 | −0.86 | 0.92 | 0.97 | 0.86 | 1.00 |
| 2015–2019[d] | MHI | MFI | SEA | SEP | SED | SES | DEP |
| MHI | 1.00 | 0.96 | 0.91 | −0.88 | −0.86 | −0.90 | −0.78 |
| MFI | 0.96 | 1.00 | 0.92 | −0.90 | −0.89 | −0.94 | −0.80 |
| SEA | 0.91 | 0.92 | 1.00 | −0.94 | −0.91 | −0.97 | −0.86 |
| SEP | −0.88 | −0.90 | −0.94 | 1.00 | 0.94 | 0.97 | 0.91 |
| SED | −0.86 | −0.89 | −0.91 | 0.94 | 1.00 | 0.91 | 0.97 |
| SES | −0.90 | −0.94 | −0.97 | 0.97 | 0.91 | 1.00 | 0.86 |
| DEP | −0.78 | −0.80 | −0.86 | 0.91 | 0.97 | 0.86 | 1.00 |

[a] Correlation matrix based on 1,920 census tracts; 30 out of 1,950 census tracts (1.54%) were omitted due to missing data.

[b] Correlation matrix based on 1,903 census tracts; 47 out of 1,950 census tracts (2.41%) were omitted due to missing data.

[c] Correlation matrix based on 1,980 census tracts; 30 out of 2,010 census tracts (1.49%) were omitted due to missing data.

[d] Correlation matrix based on 1,964 census tracts; 46 out of 2,010 census tracts (2.29%) were omitted due to missing data.

Abbreviations: MHI, Median Household Income; MFI, Median Family Income; SEA, Socioeconomic Advantage [20]; SEP, Socioeconomic Position [21]; SED, Socioeconomic Deprivation [22]; SES, Socioeconomic Status [23]; DEP, Deprivation [24].

Table S29. Relationships between simple and composite measures of neighborhood socioeconomic status in the State of New Mexico.

| 2000[a] | MHI | MFI | SEA | SEP | SED | SES | DEP |
|---|---|---|---|---|---|---|---|
| MHI | 1.00 | 0.94 | 0.89 | −0.85 | −0.86 | −0.86 | −0.76 |
| MFI | 0.94 | 1.00 | 0.92 | −0.89 | −0.89 | −0.93 | −0.78 |
| SEA | 0.89 | 0.92 | 1.00 | −0.94 | −0.93 | −0.97 | −0.86 |
| SEP | −0.85 | −0.89 | −0.94 | 1.00 | 0.96 | 0.97 | 0.92 |
| SED | −0.86 | −0.89 | −0.93 | 0.96 | 1.00 | 0.94 | 0.96 |
| SES | −0.86 | −0.93 | −0.97 | 0.97 | 0.94 | 1.00 | 0.87 |
| DEP | −0.76 | −0.78 | −0.86 | 0.92 | 0.96 | 0.87 | 1.00 |
| 2005–2009[b] | MHI | MFI | SEA | SEP | SED | SES | DEP |
| MHI | 1.00 | 0.94 | 0.85 | −0.87 | −0.87 | −0.85 | −0.77 |
| MFI | 0.94 | 1.00 | 0.88 | −0.89 | −0.88 | −0.90 | −0.79 |
| SEA | 0.85 | 0.88 | 1.00 | −0.93 | −0.92 | −0.96 | −0.85 |
| SEP | −0.87 | −0.89 | −0.93 | 1.00 | 0.95 | 0.97 | 0.92 |
| SED | −0.87 | −0.88 | −0.92 | 0.95 | 1.00 | 0.94 | 0.95 |
| SES | −0.85 | −0.90 | −0.96 | 0.97 | 0.94 | 1.00 | 0.86 |
| DEP | −0.77 | −0.79 | −0.85 | 0.92 | 0.95 | 0.86 | 1.00 |
| 2010–2014[c] | MHI | MFI | SEA | SEP | SED | SES | DEP |
| MHI | 1.00 | 0.92 | 0.82 | −0.85 | −0.87 | −0.81 | −0.78 |
| MFI | 0.92 | 1.00 | 0.88 | −0.88 | −0.89 | −0.90 | −0.80 |
| SEA | 0.82 | 0.88 | 1.00 | −0.94 | −0.92 | −0.96 | −0.85 |
| SEP | −0.85 | −0.88 | −0.94 | 1.00 | 0.96 | 0.97 | 0.92 |
| SED | −0.87 | −0.89 | −0.92 | 0.96 | 1.00 | 0.94 | 0.95 |
| SES | −0.81 | −0.90 | −0.96 | 0.97 | 0.94 | 1.00 | 0.86 |
| DEP | −0.78 | −0.80 | −0.85 | 0.92 | 0.95 | 0.86 | 1.00 |
| 2015–2019[d] | MHI | MFI | SEA | SEP | SED | SES | DEP |
| MHI | 1.00 | 0.94 | 0.84 | −0.84 | −0.88 | −0.83 | −0.77 |
| MFI | 0.94 | 1.00 | 0.88 | −0.86 | −0.90 | −0.90 | −0.79 |
| SEA | 0.84 | 0.88 | 1.00 | −0.92 | −0.92 | −0.96 | −0.84 |
| SEP | −0.84 | −0.86 | −0.92 | 1.00 | 0.93 | 0.95 | 0.90 |
| SED | −0.88 | −0.90 | −0.92 | 0.93 | 1.00 | 0.93 | 0.95 |
| SES | −0.83 | −0.90 | −0.96 | 0.95 | 0.93 | 1.00 | 0.85 |
| DEP | −0.77 | −0.79 | −0.84 | 0.90 | 0.95 | 0.85 | 1.00 |

[a] Correlation matrix based on 442 census tracts; 14 out of 456 census tracts (3.07%) were omitted due to missing data.

[b] Correlation matrix based on 436 census tracts; 20 out of 456 census tracts (4.39%) were omitted due to missing data.

[c] Correlation matrix based on 492 census tracts; 7 out of 499 census tracts (1.40%) were omitted due to missing data.

[d] Correlation matrix based on 490 census tracts; 9 out of 499 census tracts (1.80%) were omitted due to missing data.

Abbreviations: MHI, Median Household Income; MFI, Median Family Income; SEA, Socioeconomic Advantage [20]; SEP, Socioeconomic Position [21]; SED, Socioeconomic Deprivation [22]; SES, Socioeconomic Status [23]; DEP, Deprivation [24].

Table S30. Relationships between simple and composite measures of neighborhood socioeconomic status in the State of New York.

| 2000[a] | MHI | MFI | SEA | SEP | SED | SES | DEP |
|---|---|---|---|---|---|---|---|
| MHI | 1.00 | 0.93 | 0.87 | −0.87 | −0.83 | −0.87 | −0.73 |
| MFI | 0.93 | 1.00 | 0.89 | −0.89 | −0.83 | −0.93 | −0.73 |
| SEA | 0.87 | 0.89 | 1.00 | −0.94 | −0.89 | −0.96 | −0.84 |
| SEP | −0.87 | −0.89 | −0.94 | 1.00 | 0.93 | 0.97 | 0.90 |
| SED | −0.83 | −0.83 | −0.89 | 0.93 | 1.00 | 0.88 | 0.97 |
| SES | −0.87 | −0.93 | −0.96 | 0.97 | 0.88 | 1.00 | 0.83 |
| DEP | −0.73 | −0.73 | −0.84 | 0.90 | 0.97 | 0.83 | 1.00 |
| 2005–2009[b] | MHI | MFI | SEA | SEP | SED | SES | DEP |
| MHI | 1.00 | 0.92 | 0.87 | −0.86 | −0.83 | −0.84 | −0.74 |
| MFI | 0.92 | 1.00 | 0.87 | −0.87 | −0.83 | −0.91 | −0.73 |
| SEA | 0.87 | 0.87 | 1.00 | −0.94 | −0.85 | −0.95 | −0.82 |
| SEP | −0.86 | −0.87 | −0.94 | 1.00 | 0.91 | 0.97 | 0.89 |
| SED | −0.83 | −0.83 | −0.85 | 0.91 | 1.00 | 0.86 | 0.96 |
| SES | −0.84 | −0.91 | −0.95 | 0.97 | 0.86 | 1.00 | 0.83 |
| DEP | −0.74 | −0.73 | −0.82 | 0.89 | 0.96 | 0.83 | 1.00 |
| 2010–2014[c] | MHI | MFI | SEA | SEP | SED | SES | DEP |
| MHI | 1.00 | 0.92 | 0.87 | −0.86 | −0.82 | −0.84 | −0.75 |
| MFI | 0.92 | 1.00 | 0.88 | −0.88 | −0.82 | −0.91 | −0.74 |
| SEA | 0.87 | 0.88 | 1.00 | −0.94 | −0.84 | −0.95 | −0.82 |
| SEP | −0.86 | −0.88 | −0.94 | 1.00 | 0.91 | 0.98 | 0.90 |
| SED | −0.82 | −0.82 | −0.84 | 0.91 | 1.00 | 0.87 | 0.97 |
| SES | −0.84 | −0.91 | −0.95 | 0.98 | 0.87 | 1.00 | 0.85 |
| DEP | −0.75 | −0.74 | −0.82 | 0.90 | 0.97 | 0.85 | 1.00 |
| 2015–2019[d] | MHI | MFI | SEA | SEP | SED | SES | DEP |
| MHI | 1.00 | 0.93 | 0.87 | −0.86 | −0.82 | −0.85 | −0.75 |
| MFI | 0.93 | 1.00 | 0.89 | −0.88 | −0.84 | −0.91 | −0.74 |
| SEA | 0.87 | 0.89 | 1.00 | −0.92 | −0.82 | −0.94 | −0.79 |
| SEP | −0.86 | −0.88 | −0.92 | 1.00 | 0.90 | 0.97 | 0.89 |
| SED | −0.82 | −0.84 | −0.82 | 0.90 | 1.00 | 0.86 | 0.96 |
| SES | −0.85 | −0.91 | −0.94 | 0.97 | 0.86 | 1.00 | 0.82 |
| DEP | −0.75 | −0.74 | −0.79 | 0.89 | 0.96 | 0.82 | 1.00 |

[a] Correlation matrix based on 4,735 census tracts; 172 out of 4,907 census tracts (3.51%) were omitted due to missing data.

[b] Correlation matrix based on 4,625 census tracts; 282 out of 4,907 census tracts (5.75%) were omitted due to missing data.

[c] Correlation matrix based on 4,666 census tracts; 252 out of 4,918 census tracts (5.12%) were omitted due to missing data.

[d] Correlation matrix based on 4,576 census tracts; 342 out of 4,918 census tracts (6.95%) were omitted due to missing data.

Abbreviations: MHI, Median Household Income; MFI, Median Family Income; SEA, Socioeconomic Advantage [20]; SEP, Socioeconomic Position [21]; SED, Socioeconomic Deprivation [22]; SES, Socioeconomic Status [23]; DEP, Deprivation [24].

Table S31. Relationships between simple and composite measures of neighborhood socioeconomic status in the State of North Carolina.

| 2000[a] | MHI | MFI | SEA | SEP | SED | SES | DEP |
|---|---|---|---|---|---|---|---|
| MHI | 1.00 | 0.94 | 0.89 | −0.90 | −0.90 | −0.88 | −0.82 |
| MFI | 0.94 | 1.00 | 0.92 | −0.91 | −0.91 | −0.94 | −0.80 |
| SEA | 0.89 | 0.92 | 1.00 | −0.94 | −0.94 | −0.97 | −0.86 |
| SEP | −0.90 | −0.91 | −0.94 | 1.00 | 0.96 | 0.95 | 0.91 |
| SED | −0.90 | −0.91 | −0.94 | 0.96 | 1.00 | 0.93 | 0.95 |
| SES | −0.88 | −0.94 | −0.97 | 0.95 | 0.93 | 1.00 | 0.83 |
| DEP | −0.82 | −0.80 | −0.86 | 0.91 | 0.95 | 0.83 | 1.00 |
| 2005–2009[b] | MHI | MFI | SEA | SEP | SED | SES | DEP |
| MHI | 1.00 | 0.89 | 0.88 | −0.89 | −0.88 | −0.85 | −0.82 |
| MFI | 0.89 | 1.00 | 0.89 | −0.88 | −0.87 | −0.92 | −0.78 |
| SEA | 0.88 | 0.89 | 1.00 | −0.94 | −0.93 | −0.96 | −0.87 |
| SEP | −0.89 | −0.88 | −0.94 | 1.00 | 0.96 | 0.97 | 0.93 |
| SED | −0.88 | −0.87 | −0.93 | 0.96 | 1.00 | 0.93 | 0.97 |
| SES | −0.85 | −0.92 | −0.96 | 0.97 | 0.93 | 1.00 | 0.86 |
| DEP | −0.82 | −0.78 | −0.87 | 0.93 | 0.97 | 0.86 | 1.00 |
| 2010–2014[c] | MHI | MFI | SEA | SEP | SED | SES | DEP |
| MHI | 1.00 | 0.92 | 0.88 | −0.89 | −0.89 | −0.87 | −0.82 |
| MFI | 0.92 | 1.00 | 0.90 | −0.90 | −0.90 | −0.92 | −0.80 |
| SEA | 0.88 | 0.90 | 1.00 | −0.94 | −0.93 | −0.96 | −0.87 |
| SEP | −0.89 | −0.90 | −0.94 | 1.00 | 0.97 | 0.97 | 0.93 |
| SED | −0.89 | −0.90 | −0.93 | 0.97 | 1.00 | 0.95 | 0.97 |
| SES | −0.87 | −0.92 | −0.96 | 0.97 | 0.95 | 1.00 | 0.88 |
| DEP | −0.82 | −0.80 | −0.87 | 0.93 | 0.97 | 0.88 | 1.00 |
| 2015–2019[d] | MHI | MFI | SEA | SEP | SED | SES | DEP |
| MHI | 1.00 | 0.93 | 0.88 | −0.88 | −0.89 | −0.87 | −0.81 |
| MFI | 0.93 | 1.00 | 0.91 | −0.88 | −0.90 | −0.93 | −0.79 |
| SEA | 0.88 | 0.91 | 1.00 | −0.92 | −0.94 | −0.96 | −0.87 |
| SEP | −0.88 | −0.88 | −0.92 | 1.00 | 0.96 | 0.96 | 0.93 |
| SED | −0.89 | −0.90 | −0.94 | 0.96 | 1.00 | 0.94 | 0.96 |
| SES | −0.87 | −0.93 | −0.96 | 0.96 | 0.94 | 1.00 | 0.87 |
| DEP | −0.81 | −0.79 | −0.87 | 0.93 | 0.96 | 0.87 | 1.00 |

[a] Correlation matrix based on 1,548 census tracts; 15 out of 1,563 census tracts (0.96%) were omitted due to missing data.

[b] Correlation matrix based on 1,539 census tracts; 24 out of 1,563 census tracts (1.54%) were omitted due to missing data.

[c] Correlation matrix based on 2,145 census tracts; 50 out of 2,195 census tracts (2.28%) were omitted due to missing data.

[d] Correlation matrix based on 2,143 census tracts; 52 out of 2,195 census tracts (2.37%) were omitted due to missing data.

Abbreviations: MHI, Median Household Income; MFI, Median Family Income; SEA, Socioeconomic Advantage [20]; SEP, Socioeconomic Position [21]; SED, Socioeconomic Deprivation [22]; SES, Socioeconomic Status [23]; DEP, Deprivation [24].

Table S32. Relationships between simple and composite measures of neighborhood socioeconomic status in the State of North Dakota.

| 2000[a] | MHI | MFI | SEA | SEP | SED | SES | DEP |
|---|---|---|---|---|---|---|---|
| MHI | 1.00 | 0.90 | 0.79 | −0.74 | −0.78 | 0.77 | −0.45 |
| MFI | 0.90 | 1.00 | 0.81 | −0.69 | −0.81 | 0.85 | −0.49 |
| SEA | 0.79 | 0.81 | 1.00 | −0.82 | −0.81 | 0.81 | −0.61 |
| SEP | −0.74 | −0.69 | −0.82 | 1.00 | 0.83 | −0.78 | 0.74 |
| SED | −0.78 | −0.81 | −0.81 | 0.83 | 1.00 | −0.85 | 0.78 |
| SES | 0.77 | 0.85 | 0.81 | −0.78 | −0.85 | 1.00 | −0.53 |
| DEP | −0.45 | −0.49 | −0.61 | 0.74 | 0.78 | −0.53 | 1.00 |
| 2005–2009[b] | MHI | MFI | SEA | SEP | SED | SES | DEP |
| MHI | 1.00 | 0.68 | 0.82 | −0.78 | −0.80 | 0.67 | −0.54 |
| MFI | 0.68 | 1.00 | 0.70 | −0.54 | −0.74 | 0.85 | −0.50 |
| SEA | 0.82 | 0.70 | 1.00 | −0.83 | −0.82 | 0.78 | −0.62 |
| SEP | −0.78 | −0.54 | −0.83 | 1.00 | 0.85 | −0.69 | 0.77 |
| SED | −0.80 | −0.74 | −0.82 | 0.85 | 1.00 | −0.79 | 0.86 |
| SES | 0.67 | 0.85 | 0.78 | −0.69 | −0.79 | 1.00 | −0.54 |
| DEP | −0.54 | −0.50 | −0.62 | 0.77 | 0.86 | −0.54 | 1.00 |
| 2010–2014[c] | MHI | MFI | SEA | SEP | SED | SES | DEP |
| MHI | 1.00 | 0.85 | 0.81 | −0.81 | −0.82 | −0.75 | −0.60 |
| MFI | 0.85 | 1.00 | 0.82 | −0.77 | −0.86 | −0.85 | −0.66 |
| SEA | 0.81 | 0.82 | 1.00 | −0.87 | −0.83 | −0.86 | −0.66 |
| SEP | −0.81 | −0.77 | −0.87 | 1.00 | 0.89 | 0.84 | 0.78 |
| SED | −0.82 | −0.86 | −0.83 | 0.89 | 1.00 | 0.84 | 0.88 |
| SES | −0.75 | −0.85 | −0.86 | 0.84 | 0.84 | 1.00 | 0.61 |
| DEP | −0.60 | −0.66 | −0.66 | 0.78 | 0.88 | 0.61 | 1.00 |
| 2015–2019[d] | MHI | MFI | SEA | SEP | SED | SES | DEP |
| MHI | 1.00 | 0.82 | 0.78 | −0.81 | −0.80 | −0.73 | −0.64 |
| MFI | 0.82 | 1.00 | 0.77 | −0.77 | −0.83 | −0.84 | −0.67 |
| SEA | 0.78 | 0.77 | 1.00 | −0.86 | −0.81 | −0.85 | −0.67 |
| SEP | −0.81 | −0.77 | −0.86 | 1.00 | 0.90 | 0.88 | 0.82 |
| SED | −0.80 | −0.83 | −0.81 | 0.90 | 1.00 | 0.84 | 0.91 |
| SES | −0.73 | −0.84 | −0.85 | 0.88 | 0.84 | 1.00 | 0.68 |
| DEP | −0.64 | −0.67 | −0.67 | 0.82 | 0.91 | 0.68 | 1.00 |

[a] Correlation matrix based on 225 census tracts; 2 out of 227 census tracts (0.88%) were omitted due to missing data.

[b] Correlation matrix based on 218 census tracts; 9 out of 227 census tracts (3.96%) were omitted due to missing data.

[c] Correlation matrix based on 203 census tracts; 2 out of 205 census tracts (0.98%) were omitted due to missing data.

[d] Correlation matrix based on 202 census tracts; 3 out of 205 census tracts (1.46%) were omitted due to missing data.

Abbreviations: MHI, Median Household Income; MFI, Median Family Income; SEA, Socioeconomic Advantage [20]; SEP, Socioeconomic Position [21]; SED, Socioeconomic Deprivation [22]; SES, Socioeconomic Status [23]; DEP, Deprivation [24].

Table S33. Relationships between simple and composite measures of neighborhood socioeconomic status in the State of Ohio.

| 2000[a] | MHI | MFI | SEA | SEP | SED | SES | DEP |
|---|---|---|---|---|---|---|---|
| MHI | 1.00 | 0.95 | 0.90 | −0.90 | −0.89 | −0.90 | −0.80 |
| MFI | 0.95 | 1.00 | 0.91 | −0.91 | −0.90 | −0.94 | −0.80 |
| SEA | 0.90 | 0.91 | 1.00 | −0.94 | −0.94 | −0.97 | −0.89 |
| SEP | −0.90 | −0.91 | −0.94 | 1.00 | 0.95 | 0.97 | 0.91 |
| SED | −0.89 | −0.90 | −0.94 | 0.95 | 1.00 | 0.93 | 0.96 |
| SES | −0.90 | −0.94 | −0.97 | 0.97 | 0.93 | 1.00 | 0.86 |
| DEP | −0.80 | −0.80 | −0.89 | 0.91 | 0.96 | 0.86 | 1.00 |
| 2005–2009[b] | MHI | MFI | SEA | SEP | SED | SES | DEP |
| MHI | 1.00 | 0.92 | 0.89 | −0.89 | −0.89 | −0.87 | −0.81 |
| MFI | 0.92 | 1.00 | 0.90 | −0.89 | −0.89 | −0.92 | −0.78 |
| SEA | 0.89 | 0.90 | 1.00 | −0.94 | −0.93 | −0.96 | −0.89 |
| SEP | −0.89 | −0.89 | −0.94 | 1.00 | 0.95 | 0.96 | 0.92 |
| SED | −0.89 | −0.89 | −0.93 | 0.95 | 1.00 | 0.92 | 0.96 |
| SES | −0.87 | −0.92 | −0.96 | 0.96 | 0.92 | 1.00 | 0.86 |
| DEP | −0.81 | −0.78 | −0.89 | 0.92 | 0.96 | 0.86 | 1.00 |
| 2010–2014[c] | MHI | MFI | SEA | SEP | SED | SES | DEP |
| MHI | 1.00 | 0.94 | 0.91 | −0.89 | −0.90 | −0.89 | −0.83 |
| MFI | 0.94 | 1.00 | 0.92 | −0.91 | −0.92 | −0.94 | −0.82 |
| SEA | 0.91 | 0.92 | 1.00 | −0.95 | −0.94 | −0.97 | −0.89 |
| SEP | −0.89 | −0.91 | −0.95 | 1.00 | 0.96 | 0.97 | 0.93 |
| SED | −0.90 | −0.92 | −0.94 | 0.96 | 1.00 | 0.94 | 0.96 |
| SES | −0.89 | −0.94 | −0.97 | 0.97 | 0.94 | 1.00 | 0.88 |
| DEP | −0.83 | −0.82 | −0.89 | 0.93 | 0.96 | 0.88 | 1.00 |
| 2015–2019[d] | MHI | MFI | SEA | SEP | SED | SES | DEP |
| MHI | 1.00 | 0.94 | 0.90 | −0.87 | −0.90 | −0.89 | −0.81 |
| MFI | 0.94 | 1.00 | 0.92 | −0.89 | −0.92 | −0.94 | −0.81 |
| SEA | 0.90 | 0.92 | 1.00 | −0.93 | −0.94 | −0.96 | −0.88 |
| SEP | −0.87 | −0.89 | −0.93 | 1.00 | 0.95 | 0.96 | 0.92 |
| SED | −0.90 | −0.92 | −0.94 | 0.95 | 1.00 | 0.94 | 0.95 |
| SES | −0.89 | −0.94 | −0.96 | 0.96 | 0.94 | 1.00 | 0.87 |
| DEP | −0.81 | −0.81 | −0.88 | 0.92 | 0.95 | 0.87 | 1.00 |

[a] Correlation matrix based on 2,900 census tracts; 41 out of 2,941 census tracts (1.39%) were omitted due to missing data.

[b] Correlation matrix based on 2,878 census tracts; 63 out of 2,941 census tracts (2.14%) were omitted due to missing data.

[c] Correlation matrix based on 2,915 census tracts; 37 out of 2,952 census tracts (1.25%) were omitted due to missing data.

[d] Correlation matrix based on 2,882 census tracts; 70 out of 2,952 census tracts (2.37%) were omitted due to missing data.

Abbreviations: MHI, Median Household Income; MFI, Median Family Income; SEA, Socioeconomic Advantage [20]; SEP, Socioeconomic Position [21]; SED, Socioeconomic Deprivation [22]; SES, Socioeconomic Status [23]; DEP, Deprivation [24].

Table S34. Relationships between simple and composite measures of neighborhood socioeconomic status in the State of Oklahoma.

| 2000[a] | MHI | MFI | SEA | SEP | SED | SES | DEP |
|---|---|---|---|---|---|---|---|
| MHI | 1.00 | 0.95 | 0.89 | −0.87 | −0.90 | −0.88 | −0.84 |
| MFI | 0.95 | 1.00 | 0.92 | −0.90 | −0.91 | −0.93 | −0.83 |
| SEA | 0.89 | 0.92 | 1.00 | −0.93 | −0.94 | −0.96 | −0.90 |
| SEP | −0.87 | −0.90 | −0.93 | 1.00 | 0.95 | 0.95 | 0.93 |
| SED | −0.90 | −0.91 | −0.94 | 0.95 | 1.00 | 0.93 | 0.97 |
| SES | −0.88 | −0.93 | −0.96 | 0.95 | 0.93 | 1.00 | 0.87 |
| DEP | −0.84 | −0.83 | −0.90 | 0.93 | 0.97 | 0.87 | 1.00 |
| 2005–2009[b] | MHI | MFI | SEA | SEP | SED | SES | DEP |
| MHI | 1.00 | 0.91 | 0.88 | −0.87 | −0.90 | −0.86 | −0.82 |
| MFI | 0.91 | 1.00 | 0.90 | −0.89 | −0.91 | −0.92 | −0.80 |
| SEA | 0.88 | 0.90 | 1.00 | −0.94 | −0.94 | −0.96 | −0.87 |
| SEP | −0.87 | −0.89 | −0.94 | 1.00 | 0.95 | 0.96 | 0.91 |
| SED | −0.90 | −0.91 | −0.94 | 0.95 | 1.00 | 0.94 | 0.95 |
| SES | −0.86 | −0.92 | −0.96 | 0.96 | 0.94 | 1.00 | 0.86 |
| DEP | −0.82 | −0.80 | −0.87 | 0.91 | 0.95 | 0.86 | 1.00 |
| 2010–2014[c] | MHI | MFI | SEA | SEP | SED | SES | DEP |
| MHI | 1.00 | 0.92 | 0.88 | −0.88 | −0.90 | −0.86 | −0.82 |
| MFI | 0.92 | 1.00 | 0.91 | −0.89 | −0.91 | −0.93 | −0.80 |
| SEA | 0.88 | 0.91 | 1.00 | −0.95 | −0.94 | −0.96 | −0.88 |
| SEP | −0.88 | −0.89 | −0.95 | 1.00 | 0.96 | 0.96 | 0.92 |
| SED | −0.90 | −0.91 | −0.94 | 0.96 | 1.00 | 0.94 | 0.95 |
| SES | −0.86 | −0.93 | −0.96 | 0.96 | 0.94 | 1.00 | 0.86 |
| DEP | −0.82 | −0.80 | −0.88 | 0.92 | 0.95 | 0.86 | 1.00 |
| 2015–2019[d] | MHI | MFI | SEA | SEP | SED | SES | DEP |
| MHI | 1.00 | 0.93 | 0.89 | −0.88 | −0.92 | −0.87 | −0.83 |
| MFI | 0.93 | 1.00 | 0.91 | −0.89 | −0.93 | −0.93 | −0.80 |
| SEA | 0.89 | 0.91 | 1.00 | −0.94 | −0.95 | −0.96 | −0.89 |
| SEP | −0.88 | −0.89 | −0.94 | 1.00 | 0.96 | 0.96 | 0.92 |
| SED | −0.92 | −0.93 | −0.95 | 0.96 | 1.00 | 0.96 | 0.94 |
| SES | −0.87 | −0.93 | −0.96 | 0.96 | 0.96 | 1.00 | 0.87 |
| DEP | −0.83 | −0.80 | −0.89 | 0.92 | 0.94 | 0.87 | 1.00 |

[a] Correlation matrix based on 984 census tracts; 6 out of 990 census tracts (0.61%) were omitted due to missing data.

[b] Correlation matrix based on 975 census tracts; 15 out of 990 census tracts (1.52%) were omitted due to missing data.

[c] Correlation matrix based on 1,031 census tracts; 15 out of 1,046 census tracts (1.43%) were omitted due to missing data.

[d] Correlation matrix based on 1,019 census tracts; 27 out of 1,046 census tracts (2.58%) were omitted due to missing data.

Abbreviations: MHI, Median Household Income; MFI, Median Family Income; SEA, Socioeconomic Advantage [20]; SEP, Socioeconomic Position [21]; SED, Socioeconomic Deprivation [22]; SES, Socioeconomic Status [23]; DEP, Deprivation [24].

Table S35. Relationships between simple and composite measures of neighborhood socioeconomic status in the State of Oregon.

| 2000[a] | MHI | MFI | SEA | SEP | SED | SES | DEP |
|---|---|---|---|---|---|---|---|
| MHI | 1.00 | 0.91 | 0.85 | −0.86 | −0.90 | −0.81 | −0.80 |
| MFI | 0.91 | 1.00 | 0.90 | −0.89 | −0.90 | −0.91 | −0.80 |
| SEA | 0.85 | 0.90 | 1.00 | −0.93 | −0.92 | −0.96 | −0.88 |
| SEP | −0.86 | −0.89 | −0.93 | 1.00 | 0.95 | 0.94 | 0.93 |
| SED | −0.90 | −0.90 | −0.92 | 0.95 | 1.00 | 0.91 | 0.95 |
| SES | −0.81 | −0.91 | −0.96 | 0.94 | 0.91 | 1.00 | 0.87 |
| DEP | −0.80 | −0.80 | −0.88 | 0.93 | 0.95 | 0.87 | 1.00 |
| 2005–2009[b] | MHI | MFI | SEA | SEP | SED | SES | DEP |
| MHI | 1.00 | 0.85 | 0.83 | −0.83 | −0.86 | −0.74 | −0.72 |
| MFI | 0.85 | 1.00 | 0.88 | −0.85 | −0.87 | −0.89 | −0.73 |
| SEA | 0.83 | 0.88 | 1.00 | −0.92 | −0.90 | −0.94 | −0.82 |
| SEP | −0.83 | −0.85 | −0.92 | 1.00 | 0.95 | 0.93 | 0.92 |
| SED | −0.86 | −0.87 | −0.90 | 0.95 | 1.00 | 0.89 | 0.93 |
| SES | −0.74 | −0.89 | −0.94 | 0.93 | 0.89 | 1.00 | 0.84 |
| DEP | −0.72 | −0.73 | −0.82 | 0.92 | 0.93 | 0.84 | 1.00 |
| 2010–2014[c] | MHI | MFI | SEA | SEP | SED | SES | DEP |
| MHI | 1.00 | 0.89 | 0.84 | −0.85 | −0.87 | −0.79 | −0.74 |
| MFI | 0.89 | 1.00 | 0.89 | −0.88 | −0.90 | −0.90 | −0.76 |
| SEA | 0.84 | 0.89 | 1.00 | −0.93 | −0.92 | −0.95 | −0.84 |
| SEP | −0.85 | −0.88 | −0.93 | 1.00 | 0.95 | 0.95 | 0.91 |
| SED | −0.87 | −0.90 | −0.92 | 0.95 | 1.00 | 0.92 | 0.93 |
| SES | −0.79 | −0.90 | −0.95 | 0.95 | 0.92 | 1.00 | 0.84 |
| DEP | −0.74 | −0.76 | −0.84 | 0.91 | 0.93 | 0.84 | 1.00 |
| 2015–2019[d] | MHI | MFI | SEA | SEP | SED | SES | DEP |
| MHI | 1.00 | 0.90 | 0.87 | −0.86 | −0.90 | −0.82 | −0.75 |
| MFI | 0.90 | 1.00 | 0.89 | −0.87 | −0.91 | −0.92 | −0.74 |
| SEA | 0.87 | 0.89 | 1.00 | −0.91 | −0.93 | −0.95 | −0.84 |
| SEP | −0.86 | −0.87 | −0.91 | 1.00 | 0.95 | 0.93 | 0.90 |
| SED | −0.90 | −0.91 | −0.93 | 0.95 | 1.00 | 0.93 | 0.92 |
| SES | −0.82 | −0.92 | −0.95 | 0.93 | 0.93 | 1.00 | 0.83 |
| DEP | −0.75 | −0.74 | −0.84 | 0.90 | 0.92 | 0.83 | 1.00 |

[a] Correlation matrix based on 753 census tracts; 2 out of 755 census tracts (0.26%) were omitted due to missing data.

[b] Correlation matrix based on 751 census tracts; 4 out of 755 census tracts (0.53%) were omitted due to missing data.

[c] Correlation matrix based on 822 census tracts; 12 out of 834 census tracts (1.44%) were omitted due to missing data.

[d] Correlation matrix based on 819 census tracts; 15 out of 834 census tracts (1.80%) were omitted due to missing data.

Abbreviations: MHI, Median Household Income; MFI, Median Family Income; SEA, Socioeconomic Advantage [20]; SEP, Socioeconomic Position [21]; SED, Socioeconomic Deprivation [22]; SES, Socioeconomic Status [23]; DEP, Deprivation [24].

Table S36. Relationships between simple and composite measures of neighborhood socioeconomic status in the State of Pennsylvania.

| 2000[a] | MHI | MFI | SEA | SEP | SED | SES | DEP |
|---|---|---|---|---|---|---|---|
| MHI | 1.00 | 0.95 | 0.89 | −0.87 | −0.87 | −0.89 | −0.72 |
| MFI | 0.95 | 1.00 | 0.91 | −0.88 | −0.87 | −0.94 | −0.72 |
| SEA | 0.89 | 0.91 | 1.00 | −0.94 | −0.95 | −0.97 | −0.87 |
| SEP | −0.87 | −0.88 | −0.94 | 1.00 | 0.94 | 0.96 | 0.89 |
| SED | −0.87 | −0.87 | −0.95 | 0.94 | 1.00 | 0.92 | 0.95 |
| SES | −0.89 | −0.94 | −0.97 | 0.96 | 0.92 | 1.00 | 0.82 |
| DEP | −0.72 | −0.72 | −0.87 | 0.89 | 0.95 | 0.82 | 1.00 |
| 2005–2009[b] | MHI | MFI | SEA | SEP | SED | SES | DEP |
| MHI | 1.00 | 0.93 | 0.90 | −0.88 | −0.88 | −0.88 | −0.75 |
| MFI | 0.93 | 1.00 | 0.92 | −0.89 | −0.90 | −0.94 | −0.75 |
| SEA | 0.90 | 0.92 | 1.00 | −0.94 | −0.93 | −0.96 | −0.86 |
| SEP | −0.88 | −0.89 | −0.94 | 1.00 | 0.95 | 0.97 | 0.91 |
| SED | −0.88 | −0.90 | −0.93 | 0.95 | 1.00 | 0.93 | 0.95 |
| SES | −0.88 | −0.94 | −0.96 | 0.97 | 0.93 | 1.00 | 0.84 |
| DEP | −0.75 | −0.75 | −0.86 | 0.91 | 0.95 | 0.84 | 1.00 |
| 2010–2014[c] | MHI | MFI | SEA | SEP | SED | SES | DEP |
| MHI | 1.00 | 0.93 | 0.90 | −0.87 | −0.88 | −0.87 | −0.76 |
| MFI | 0.93 | 1.00 | 0.92 | −0.90 | −0.90 | −0.94 | −0.75 |
| SEA | 0.90 | 0.92 | 1.00 | −0.95 | −0.93 | −0.96 | −0.85 |
| SEP | −0.87 | −0.90 | −0.95 | 1.00 | 0.95 | 0.97 | 0.91 |
| SED | −0.88 | −0.90 | −0.93 | 0.95 | 1.00 | 0.93 | 0.95 |
| SES | −0.87 | −0.94 | −0.96 | 0.97 | 0.93 | 1.00 | 0.85 |
| DEP | −0.76 | −0.75 | −0.85 | 0.91 | 0.95 | 0.85 | 1.00 |
| 2015–2019[d] | MHI | MFI | SEA | SEP | SED | SES | DEP |
| MHI | 1.00 | 0.93 | 0.90 | −0.86 | −0.88 | −0.87 | −0.76 |
| MFI | 0.93 | 1.00 | 0.92 | −0.88 | −0.91 | −0.94 | −0.76 |
| SEA | 0.90 | 0.92 | 1.00 | −0.93 | −0.93 | −0.96 | −0.85 |
| SEP | −0.86 | −0.88 | −0.93 | 1.00 | 0.95 | 0.96 | 0.91 |
| SED | −0.88 | −0.91 | −0.93 | 0.95 | 1.00 | 0.92 | 0.95 |
| SES | −0.87 | −0.94 | −0.96 | 0.96 | 0.92 | 1.00 | 0.83 |
| DEP | −0.76 | −0.76 | −0.85 | 0.91 | 0.95 | 0.83 | 1.00 |

[a] Correlation matrix based on 3,106 census tracts; 29 out of 3,135 census tracts (0.93%) were omitted due to missing data.

[b] Correlation matrix based on 3,082 census tracts; 53 out of 3,135 census tracts (1.69%) were omitted due to missing data.

[c] Correlation matrix based on 3,167 census tracts; 51 out of 3,218 census tracts (1.58%) were omitted due to missing data.

[d] Correlation matrix based on 3,151 census tracts; 67 out of 3,218 census tracts (2.08%) were omitted due to missing data.

Abbreviations: MHI, Median Household Income; MFI, Median Family Income; SEA, Socioeconomic Advantage [20]; SEP, Socioeconomic Position [21]; SED, Socioeconomic Deprivation [22]; SES, Socioeconomic Status [23]; DEP, Deprivation [24].

Table S37. Relationships between simple and composite measures of neighborhood socioeconomic status in the State of Rhode Island.

| 2000[a] | MHI | MFI | SEA | SEP | SED | SES | DEP |
|---|---|---|---|---|---|---|---|
| MHI | 1.00 | 0.85 | 0.88 | −0.89 | −0.87 | −0.82 | −0.82 |
| MFI | 0.85 | 1.00 | 0.92 | −0.88 | −0.88 | −0.94 | −0.81 |
| SEA | 0.88 | 0.92 | 1.00 | −0.97 | −0.95 | −0.97 | −0.90 |
| SEP | −0.89 | −0.88 | −0.97 | 1.00 | 0.95 | 0.95 | 0.91 |
| SED | −0.87 | −0.88 | −0.95 | 0.95 | 1.00 | 0.91 | 0.97 |
| SES | −0.82 | −0.94 | −0.97 | 0.95 | 0.91 | 1.00 | 0.85 |
| DEP | −0.82 | −0.81 | −0.90 | 0.91 | 0.97 | 0.85 | 1.00 |
| 2005–2009[b] | MHI | MFI | SEA | SEP | SED | SES | DEP |
| MHI | 1.00 | 0.92 | 0.86 | −0.88 | −0.88 | −0.84 | −0.80 |
| MFI | 0.92 | 1.00 | 0.91 | −0.91 | −0.89 | −0.92 | −0.81 |
| SEA | 0.86 | 0.91 | 1.00 | −0.95 | −0.90 | −0.97 | −0.85 |
| SEP | −0.88 | −0.91 | −0.95 | 1.00 | 0.95 | 0.98 | 0.92 |
| SED | −0.88 | −0.89 | −0.90 | 0.95 | 1.00 | 0.90 | 0.96 |
| SES | −0.84 | −0.92 | −0.97 | 0.98 | 0.90 | 1.00 | 0.87 |
| DEP | −0.80 | −0.81 | −0.85 | 0.92 | 0.96 | 0.87 | 1.00 |
| 2010–2014[c] | MHI | MFI | SEA | SEP | SED | SES | DEP |
| MHI | 1.00 | 0.83 | 0.88 | −0.89 | −0.88 | −0.82 | −0.84 |
| MFI | 0.83 | 1.00 | 0.89 | −0.88 | −0.88 | −0.93 | −0.81 |
| SEA | 0.88 | 0.89 | 1.00 | −0.96 | −0.92 | −0.96 | −0.89 |
| SEP | −0.89 | −0.88 | −0.96 | 1.00 | 0.96 | 0.97 | 0.94 |
| SED | −0.88 | −0.88 | −0.92 | 0.96 | 1.00 | 0.92 | 0.97 |
| SES | −0.82 | −0.93 | −0.96 | 0.97 | 0.92 | 1.00 | 0.88 |
| DEP | −0.84 | −0.81 | −0.89 | 0.94 | 0.97 | 0.88 | 1.00 |
| 2015–2019[d] | MHI | MFI | SEA | SEP | SED | SES | DEP |
| MHI | 1.00 | 0.90 | 0.86 | −0.88 | −0.89 | −0.84 | −0.83 |
| MFI | 0.90 | 1.00 | 0.90 | −0.86 | −0.88 | −0.91 | −0.78 |
| SEA | 0.86 | 0.90 | 1.00 | −0.91 | −0.88 | −0.96 | −0.80 |
| SEP | −0.88 | −0.86 | −0.91 | 1.00 | 0.96 | 0.96 | 0.92 |
| SED | −0.89 | −0.88 | −0.88 | 0.96 | 1.00 | 0.91 | 0.97 |
| SES | −0.84 | −0.91 | −0.96 | 0.96 | 0.91 | 1.00 | 0.85 |
| DEP | −0.83 | −0.78 | −0.80 | 0.92 | 0.97 | 0.85 | 1.00 |

[a] Correlation matrix based on 232 census tracts; 2 out of 234 census tracts (0.85%) were omitted due to missing data.

[b] Correlation matrix based on 230 census tracts; 4 out of 234 census tracts (1.71%) were omitted due to missing data.

[c] Correlation matrix based on 238 census tracts; 6 out of 244 census tracts (2.46%) were omitted due to missing data.

[d] Correlation matrix based on 237 census tracts; 7 out of 244 census tracts (2.87%) were omitted due to missing data.

Abbreviations: MHI, Median Household Income; MFI, Median Family Income; SEA, Socioeconomic Advantage [20]; SEP, Socioeconomic Position [21]; SED, Socioeconomic Deprivation [22]; SES, Socioeconomic Status [23]; DEP, Deprivation [24].

Table S38. Relationships between simple and composite measures of neighborhood socioeconomic status in the State of South Carolina.

| 2000[a] | MHI | MFI | SEA | SEP | SED | SES | DEP |
|---|---|---|---|---|---|---|---|
| MHI | 1.00 | 0.91 | 0.89 | −0.90 | −0.91 | −0.86 | −0.89 |
| MFI | 0.91 | 1.00 | 0.93 | −0.91 | −0.93 | −0.94 | −0.87 |
| SEA | 0.89 | 0.93 | 1.00 | −0.95 | −0.94 | −0.97 | −0.90 |
| SEP | −0.90 | −0.91 | −0.95 | 1.00 | 0.96 | 0.96 | 0.93 |
| SED | −0.91 | −0.93 | −0.94 | 0.96 | 1.00 | 0.94 | 0.96 |
| SES | −0.86 | −0.94 | −0.97 | 0.96 | 0.94 | 1.00 | 0.87 |
| DEP | −0.89 | −0.87 | −0.90 | 0.93 | 0.96 | 0.87 | 1.00 |
| 2005–2009[b] | MHI | MFI | SEA | SEP | SED | SES | DEP |
| MHI | 1.00 | 0.85 | 0.89 | −0.90 | −0.91 | −0.85 | −0.86 |
| MFI | 0.85 | 1.00 | 0.86 | −0.85 | −0.87 | −0.90 | −0.78 |
| SEA | 0.89 | 0.86 | 1.00 | −0.95 | −0.92 | −0.95 | −0.86 |
| SEP | −0.90 | −0.85 | −0.95 | 1.00 | 0.96 | 0.97 | 0.92 |
| SED | −0.91 | −0.87 | −0.92 | 0.96 | 1.00 | 0.93 | 0.97 |
| SES | −0.85 | −0.90 | −0.95 | 0.97 | 0.93 | 1.00 | 0.85 |
| DEP | −0.86 | −0.78 | −0.86 | 0.92 | 0.97 | 0.85 | 1.00 |
| 2010–2014[c] | MHI | MFI | SEA | SEP | SED | SES | DEP |
| MHI | 1.00 | 0.90 | 0.88 | −0.90 | −0.91 | −0.86 | −0.87 |
| MFI | 0.90 | 1.00 | 0.90 | −0.89 | −0.91 | −0.92 | −0.82 |
| SEA | 0.88 | 0.90 | 1.00 | −0.94 | −0.93 | −0.96 | −0.86 |
| SEP | −0.90 | −0.89 | −0.94 | 1.00 | 0.97 | 0.97 | 0.94 |
| SED | −0.91 | −0.91 | −0.93 | 0.97 | 1.00 | 0.95 | 0.96 |
| SES | −0.86 | −0.92 | −0.96 | 0.97 | 0.95 | 1.00 | 0.88 |
| DEP | −0.87 | −0.82 | −0.86 | 0.94 | 0.96 | 0.88 | 1.00 |
| 2015–2019[d] | MHI | MFI | SEA | SEP | SED | SES | DEP |
| MHI | 1.00 | 0.89 | 0.90 | −0.90 | −0.92 | −0.88 | −0.86 |
| MFI | 0.89 | 1.00 | 0.90 | −0.88 | −0.90 | −0.93 | −0.80 |
| SEA | 0.90 | 0.90 | 1.00 | −0.93 | −0.93 | −0.96 | −0.88 |
| SEP | −0.90 | −0.88 | −0.93 | 1.00 | 0.95 | 0.96 | 0.92 |
| SED | −0.92 | −0.90 | −0.93 | 0.95 | 1.00 | 0.94 | 0.96 |
| SES | −0.88 | −0.93 | −0.96 | 0.96 | 0.94 | 1.00 | 0.86 |
| DEP | −0.86 | −0.80 | −0.88 | 0.92 | 0.96 | 0.86 | 1.00 |

[a] Correlation matrix based on 856 census tracts; 11 out of 867 census tracts (1.27%) were omitted due to missing data.

[b] Correlation matrix based on 855 census tracts; 12 out of 867 census tracts (1.38%) were omitted due to missing data.

[c] Correlation matrix based on 1,076 census tracts; 27 out of 1,103 census tracts (2.45%) were omitted due to missing data.

[d] Correlation matrix based on 1,072 census tracts; 31 out of 1,103 census tracts (2.81%) were omitted due to missing data.

Abbreviations: MHI, Median Household Income; MFI, Median Family Income; SEA, Socioeconomic Advantage [20]; SEP, Socioeconomic Position [21]; SED, Socioeconomic Deprivation [22]; SES, Socioeconomic Status [23]; DEP, Deprivation [24].

Table S39. Relationships between simple and composite measures of neighborhood socioeconomic status in the State of South Dakota.

| 2000[a] | MHI | MFI | SEA | SEP | SED | SES | DEP |
|---|---|---|---|---|---|---|---|
| MHI | 1.00 | 0.91 | 0.84 | −0.78 | −0.85 | 0.77 | −0.68 |
| MFI | 0.91 | 1.00 | 0.86 | −0.78 | −0.90 | 0.87 | −0.77 |
| SEA | 0.84 | 0.86 | 1.00 | −0.85 | −0.83 | 0.77 | −0.75 |
| SEP | −0.78 | −0.78 | −0.85 | 1.00 | 0.86 | −0.82 | 0.84 |
| SED | −0.85 | −0.90 | −0.83 | 0.86 | 1.00 | −0.92 | 0.91 |
| SES | 0.77 | 0.87 | 0.77 | −0.82 | −0.92 | 1.00 | −0.82 |
| DEP | −0.68 | −0.77 | −0.75 | 0.84 | 0.91 | −0.82 | 1.00 |
| 2005–2009[b] | MHI | MFI | SEA | SEP | SED | SES | DEP |
| MHI | 1.00 | 0.88 | 0.80 | −0.82 | −0.85 | −0.82 | −0.61 |
| MFI | 0.88 | 1.00 | 0.80 | −0.81 | −0.90 | −0.91 | −0.70 |
| SEA | 0.80 | 0.80 | 1.00 | −0.85 | −0.83 | −0.86 | −0.66 |
| SEP | −0.82 | −0.81 | −0.85 | 1.00 | 0.86 | 0.89 | 0.77 |
| SED | −0.85 | −0.90 | −0.83 | 0.86 | 1.00 | 0.93 | 0.86 |
| SES | −0.82 | −0.91 | −0.86 | 0.89 | 0.93 | 1.00 | 0.75 |
| DEP | −0.61 | −0.70 | −0.66 | 0.77 | 0.86 | 0.75 | 1.00 |
| 2010–2014[c] | MHI | MFI | SEA | SEP | SED | SES | DEP |
| MHI | 1.00 | 0.91 | 0.83 | −0.84 | −0.85 | −0.87 | −0.66 |
| MFI | 0.91 | 1.00 | 0.86 | −0.86 | −0.90 | −0.94 | −0.76 |
| SEA | 0.83 | 0.86 | 1.00 | −0.91 | −0.85 | −0.91 | −0.76 |
| SEP | −0.84 | −0.86 | −0.91 | 1.00 | 0.90 | 0.93 | 0.83 |
| SED | −0.85 | −0.90 | −0.85 | 0.90 | 1.00 | 0.93 | 0.91 |
| SES | −0.87 | −0.94 | −0.91 | 0.93 | 0.93 | 1.00 | 0.81 |
| DEP | −0.66 | −0.76 | −0.76 | 0.83 | 0.91 | 0.81 | 1.00 |
| 2015–2019[d] | MHI | MFI | SEA | SEP | SED | SES | DEP |
| MHI | 1.00 | 0.89 | 0.85 | −0.84 | −0.83 | −0.85 | −0.63 |
| MFI | 0.89 | 1.00 | 0.87 | −0.84 | −0.88 | −0.93 | −0.72 |
| SEA | 0.85 | 0.87 | 1.00 | −0.91 | −0.87 | −0.91 | −0.74 |
| SEP | −0.84 | −0.84 | −0.91 | 1.00 | 0.91 | 0.92 | 0.84 |
| SED | −0.83 | −0.88 | −0.87 | 0.91 | 1.00 | 0.93 | 0.92 |
| SES | −0.85 | −0.93 | −0.91 | 0.92 | 0.93 | 1.00 | 0.82 |
| DEP | −0.63 | −0.72 | −0.74 | 0.84 | 0.92 | 0.82 | 1.00 |

[a] Correlation matrix based on 226 census tracts; 9 out of 235 census tracts (3.83%) were omitted due to missing data.

[b] Correlation matrix based on 221 census tracts; 14 out of 235 census tracts (5.96%) were omitted due to missing data.

[c] Correlation matrix based on 221 census tracts; 1 out of 222 census tracts (0.45%) were omitted due to missing data.

[d] Correlation matrix based on 220 census tracts; 2 out of 222 census tracts (0.90%) were omitted due to missing data.

Abbreviations: MHI, Median Household Income; MFI, Median Family Income; SEA, Socioeconomic Advantage [20]; SEP, Socioeconomic Position [21]; SED, Socioeconomic Deprivation [22]; SES, Socioeconomic Status [23]; DEP, Deprivation [24].

Table S40. Relationships between simple and composite measures of neighborhood socioeconomic status in the State of Tennessee.

| 2000[a] | MHI | MFI | SEA | SEP | SED | SES | DEP |
|---|---|---|---|---|---|---|---|
| MHI | 1.00 | 0.95 | 0.89 | −0.87 | −0.90 | −0.88 | −0.78 |
| MFI | 0.95 | 1.00 | 0.93 | −0.90 | −0.91 | −0.94 | −0.79 |
| SEA | 0.89 | 0.93 | 1.00 | −0.94 | −0.94 | −0.96 | −0.86 |
| SEP | −0.87 | −0.90 | −0.94 | 1.00 | 0.95 | 0.96 | 0.89 |
| SED | −0.90 | −0.91 | −0.94 | 0.95 | 1.00 | 0.93 | 0.93 |
| SES | −0.88 | −0.94 | −0.96 | 0.96 | 0.93 | 1.00 | 0.81 |
| DEP | −0.78 | −0.79 | −0.86 | 0.89 | 0.93 | 0.81 | 1.00 |
| 2005–2009[b] | MHI | MFI | SEA | SEP | SED | SES | DEP |
| MHI | 1.00 | 0.92 | 0.88 | −0.89 | −0.89 | −0.88 | −0.79 |
| MFI | 0.92 | 1.00 | 0.90 | −0.90 | −0.90 | −0.93 | −0.78 |
| SEA | 0.88 | 0.90 | 1.00 | −0.94 | −0.93 | −0.96 | −0.84 |
| SEP | −0.89 | −0.90 | −0.94 | 1.00 | 0.95 | 0.97 | 0.90 |
| SED | −0.89 | −0.90 | −0.93 | 0.95 | 1.00 | 0.93 | 0.94 |
| SES | −0.88 | −0.93 | −0.96 | 0.97 | 0.93 | 1.00 | 0.82 |
| DEP | −0.79 | −0.78 | −0.84 | 0.90 | 0.94 | 0.82 | 1.00 |
| 2010–2014[c] | MHI | MFI | SEA | SEP | SED | SES | DEP |
| MHI | 1.00 | 0.93 | 0.89 | −0.90 | −0.90 | −0.89 | −0.81 |
| MFI | 0.93 | 1.00 | 0.92 | −0.92 | −0.92 | −0.94 | −0.81 |
| SEA | 0.89 | 0.92 | 1.00 | −0.95 | −0.93 | −0.96 | −0.87 |
| SEP | −0.90 | −0.92 | −0.95 | 1.00 | 0.96 | 0.98 | 0.92 |
| SED | −0.90 | −0.92 | −0.93 | 0.96 | 1.00 | 0.94 | 0.95 |
| SES | −0.89 | −0.94 | −0.96 | 0.98 | 0.94 | 1.00 | 0.86 |
| DEP | −0.81 | −0.81 | −0.87 | 0.92 | 0.95 | 0.86 | 1.00 |
| 2015–2019[d] | MHI | MFI | SEA | SEP | SED | SES | DEP |
| MHI | 1.00 | 0.93 | 0.90 | −0.89 | −0.90 | −0.89 | −0.81 |
| MFI | 0.93 | 1.00 | 0.93 | −0.89 | −0.92 | −0.94 | −0.79 |
| SEA | 0.90 | 0.93 | 1.00 | −0.93 | −0.94 | −0.96 | −0.87 |
| SEP | −0.89 | −0.89 | −0.93 | 1.00 | 0.96 | 0.96 | 0.92 |
| SED | −0.90 | −0.92 | −0.94 | 0.96 | 1.00 | 0.95 | 0.95 |
| SES | −0.89 | −0.94 | −0.96 | 0.96 | 0.95 | 1.00 | 0.86 |
| DEP | −0.81 | −0.79 | −0.87 | 0.92 | 0.95 | 0.86 | 1.00 |

[a] Correlation matrix based on 1,249 census tracts; 12 out of 1,261 census tracts (0.95%) were omitted due to missing data.

[b] Correlation matrix based on 1,238 census tracts; 23 out of 1,261 census tracts (1.82%) were omitted due to missing data.

[c] Correlation matrix based on 1,463 census tracts; 34 out of 1,497 census tracts (2.27%) were omitted due to missing data.

[d] Correlation matrix based on 1,455 census tracts; 42 out of 1,497 census tracts (2.81%) were omitted due to missing data.

Abbreviations: MHI, Median Household Income; MFI, Median Family Income; SEA, Socioeconomic Advantage [20]; SEP, Socioeconomic Position [21]; SED, Socioeconomic Deprivation [22]; SES, Socioeconomic Status [23]; DEP, Deprivation [24].

Table S41. Relationships between simple and composite measures of neighborhood socioeconomic status in the State of Texas.

| 2000[a] | MHI | MFI | SEA | SEP | SED | SES | DEP |
|---|---|---|---|---|---|---|---|
| MHI | 1.00 | 0.95 | 0.87 | −0.87 | −0.86 | −0.88 | −0.76 |
| MFI | 0.95 | 1.00 | 0.91 | −0.89 | −0.89 | −0.93 | −0.78 |
| SEA | 0.87 | 0.91 | 1.00 | −0.95 | −0.96 | −0.97 | −0.90 |
| SEP | −0.87 | −0.89 | −0.95 | 1.00 | 0.96 | 0.97 | 0.93 |
| SED | −0.86 | −0.89 | −0.96 | 0.96 | 1.00 | 0.95 | 0.96 |
| SES | −0.88 | −0.93 | −0.97 | 0.97 | 0.95 | 1.00 | 0.88 |
| DEP | −0.76 | −0.78 | −0.90 | 0.93 | 0.96 | 0.88 | 1.00 |

| 2005–2009[b] | MHI | MFI | SEA | SEP | SED | SES | DEP |
|---|---|---|---|---|---|---|---|
| MHI | 1.00 | 0.94 | 0.87 | −0.88 | −0.87 | −0.87 | −0.78 |
| MFI | 0.94 | 1.00 | 0.90 | −0.90 | −0.89 | −0.93 | −0.79 |
| SEA | 0.87 | 0.90 | 1.00 | −0.94 | −0.95 | −0.96 | −0.90 |
| SEP | −0.88 | −0.90 | −0.94 | 1.00 | 0.95 | 0.97 | 0.93 |
| SED | −0.87 | −0.89 | −0.95 | 0.95 | 1.00 | 0.93 | 0.96 |
| SES | −0.87 | −0.93 | −0.96 | 0.97 | 0.93 | 1.00 | 0.87 |
| DEP | −0.78 | −0.79 | −0.90 | 0.93 | 0.96 | 0.87 | 1.00 |

| 2010–2014[c] | MHI | MFI | SEA | SEP | SED | SES | DEP |
|---|---|---|---|---|---|---|---|
| MHI | 1.00 | 0.94 | 0.88 | −0.89 | −0.88 | −0.88 | −0.80 |
| MFI | 0.94 | 1.00 | 0.91 | −0.91 | −0.90 | −0.93 | −0.81 |
| SEA | 0.88 | 0.91 | 1.00 | −0.95 | −0.94 | −0.97 | −0.89 |
| SEP | −0.89 | −0.91 | −0.95 | 1.00 | 0.96 | 0.98 | 0.93 |
| SED | −0.88 | −0.90 | −0.94 | 0.96 | 1.00 | 0.94 | 0.97 |
| SES | −0.88 | −0.93 | −0.97 | 0.98 | 0.94 | 1.00 | 0.89 |
| DEP | −0.80 | −0.81 | −0.89 | 0.93 | 0.97 | 0.89 | 1.00 |

| 2015–2019[d] | MHI | MFI | SEA | SEP | SED | SES | DEP |
|---|---|---|---|---|---|---|---|
| MHI | 1.00 | 0.94 | 0.89 | −0.88 | −0.89 | −0.88 | −0.79 |
| MFI | 0.94 | 1.00 | 0.92 | −0.89 | −0.91 | −0.93 | −0.81 |
| SEA | 0.89 | 0.92 | 1.00 | −0.94 | −0.95 | −0.97 | −0.89 |
| SEP | −0.88 | −0.89 | −0.94 | 1.00 | 0.95 | 0.97 | 0.93 |
| SED | −0.89 | −0.91 | −0.95 | 0.95 | 1.00 | 0.94 | 0.96 |
| SES | −0.88 | −0.93 | −0.97 | 0.97 | 0.94 | 1.00 | 0.88 |
| DEP | −0.79 | −0.81 | −0.89 | 0.93 | 0.96 | 0.88 | 1.00 |

[a] Correlation matrix based on 4,351 census tracts; 37 out of 4,388 census tracts (0.84%) were omitted due to missing data.

[b] Correlation matrix based on 4,308 census tracts; 82 out of 4,390 census tracts (1.87%) were omitted due to missing data.

[c] Correlation matrix based on 5,117 census tracts; 148 out of 5,265 census tracts (2.81%) were omitted due to missing data.

[d] Correlation matrix based on 5,082 census tracts; 183 out of 5,265 census tracts (3.48%) were omitted due to missing data.

Abbreviations: MHI, Median Household Income; MFI, Median Family Income; SEA, Socioeconomic Advantage [20]; SEP, Socioeconomic Position [21]; SED, Socioeconomic Deprivation [22]; SES, Socioeconomic Status [23]; DEP, Deprivation [24].

Table S42. Relationships between simple and composite measures of neighborhood socioeconomic status in the State of Utah.

| 2000[a] | MHI | MFI | SEA | SEP | SED | SES | DEP |
|---|---|---|---|---|---|---|---|
| MHI | 1.00 | 0.97 | 0.85 | −0.83 | −0.87 | −0.84 | −0.81 |
| MFI | 0.97 | 1.00 | 0.88 | −0.84 | −0.86 | −0.87 | −0.81 |
| SEA | 0.85 | 0.88 | 1.00 | −0.92 | −0.85 | −0.96 | −0.86 |
| SEP | −0.83 | −0.84 | −0.92 | 1.00 | 0.92 | 0.96 | 0.92 |
| SED | −0.87 | −0.86 | −0.85 | 0.92 | 1.00 | 0.88 | 0.93 |
| SES | −0.84 | −0.87 | −0.96 | 0.96 | 0.88 | 1.00 | 0.88 |
| DEP | −0.81 | −0.81 | −0.86 | 0.92 | 0.93 | 0.88 | 1.00 |
| 2005–2009[b] | MHI | MFI | SEA | SEP | SED | SES | DEP |
| MHI | 1.00 | 0.95 | 0.84 | −0.85 | −0.90 | −0.81 | −0.78 |
| MFI | 0.95 | 1.00 | 0.88 | −0.88 | −0.90 | −0.87 | −0.77 |
| SEA | 0.84 | 0.88 | 1.00 | −0.93 | −0.87 | −0.96 | −0.81 |
| SEP | −0.85 | −0.88 | −0.93 | 1.00 | 0.92 | 0.96 | 0.87 |
| SED | −0.90 | −0.90 | −0.87 | 0.92 | 1.00 | 0.87 | 0.94 |
| SES | −0.81 | −0.87 | −0.96 | 0.96 | 0.87 | 1.00 | 0.79 |
| DEP | −0.78 | −0.77 | −0.81 | 0.87 | 0.94 | 0.79 | 1.00 |
| 2010–2014[c] | MHI | MFI | SEA | SEP | SED | SES | DEP |
| MHI | 1.00 | 0.95 | 0.86 | −0.87 | −0.91 | −0.84 | −0.80 |
| MFI | 0.95 | 1.00 | 0.90 | −0.89 | −0.92 | −0.89 | −0.81 |
| SEA | 0.86 | 0.90 | 1.00 | −0.92 | −0.90 | −0.95 | −0.85 |
| SEP | −0.87 | −0.89 | −0.92 | 1.00 | 0.94 | 0.97 | 0.92 |
| SED | −0.91 | −0.92 | −0.90 | 0.94 | 1.00 | 0.91 | 0.95 |
| SES | −0.84 | −0.89 | −0.95 | 0.97 | 0.91 | 1.00 | 0.88 |
| DEP | −0.80 | −0.81 | −0.85 | 0.92 | 0.95 | 0.88 | 1.00 |
| 2015–2019[d] | MHI | MFI | SEA | SEP | SED | SES | DEP |
| MHI | 1.00 | 0.94 | 0.86 | −0.82 | −0.90 | −0.81 | −0.78 |
| MFI | 0.94 | 1.00 | 0.91 | −0.86 | −0.90 | −0.89 | −0.77 |
| SEA | 0.86 | 0.91 | 1.00 | −0.90 | −0.89 | −0.95 | −0.81 |
| SEP | −0.82 | −0.86 | −0.90 | 1.00 | 0.93 | 0.96 | 0.91 |
| SED | −0.90 | −0.90 | −0.89 | 0.93 | 1.00 | 0.90 | 0.94 |
| SES | −0.81 | −0.89 | −0.95 | 0.96 | 0.90 | 1.00 | 0.84 |
| DEP | −0.78 | −0.77 | −0.81 | 0.91 | 0.94 | 0.84 | 1.00 |

[a] Correlation matrix based on 487 census tracts; 9 out of 496 census tracts (1.81%) were omitted due to missing data.

[b] Correlation matrix based on 481 census tracts; 15 out of 496 census tracts (3.02%) were omitted due to missing data.

[c] Correlation matrix based on 577 census tracts; 11 out of 588 census tracts (1.87%) were omitted due to missing data.

[d] Correlation matrix based on 575 census tracts; 13 out of 588 census tracts (2.21%) were omitted due to missing data.

Abbreviations: MHI, Median Household Income; MFI, Median Family Income; SEA, Socioeconomic Advantage [20]; SEP, Socioeconomic Position [21]; SED, Socioeconomic Deprivation [22]; SES, Socioeconomic Status [23]; DEP, Deprivation [24].

Table S43. Relationships between simple and composite measures of neighborhood socioeconomic status in the State of Vermont.

| 2000[a] | MHI | MFI | SEA | SEP | SED | SES | DEP |
|---|---|---|---|---|---|---|---|
| MHI | 1.00 | 0.92 | 0.89 | −0.89 | −0.94 | −0.85 | −0.93 |
| MFI | 0.92 | 1.00 | 0.92 | −0.88 | −0.95 | −0.93 | −0.87 |
| SEA | 0.89 | 0.92 | 1.00 | −0.94 | −0.95 | −0.97 | −0.89 |
| SEP | −0.89 | −0.88 | −0.94 | 1.00 | 0.94 | 0.94 | 0.93 |
| SED | −0.94 | −0.95 | −0.95 | 0.94 | 1.00 | 0.94 | 0.95 |
| SES | −0.85 | −0.93 | −0.97 | 0.94 | 0.94 | 1.00 | 0.86 |
| DEP | −0.93 | −0.87 | −0.89 | 0.93 | 0.95 | 0.86 | 1.00 |
| 2005–2009[b] | MHI | MFI | SEA | SEP | SED | SES | DEP |
| MHI | 1.00 | 0.81 | 0.85 | −0.88 | −0.91 | −0.79 | −0.89 |
| MFI | 0.81 | 1.00 | 0.83 | −0.78 | −0.86 | −0.87 | −0.74 |
| SEA | 0.85 | 0.83 | 1.00 | −0.93 | −0.91 | −0.95 | −0.82 |
| SEP | −0.88 | −0.78 | −0.93 | 1.00 | 0.95 | 0.92 | 0.90 |
| SED | −0.91 | −0.86 | −0.91 | 0.95 | 1.00 | 0.90 | 0.93 |
| SES | −0.79 | −0.87 | −0.95 | 0.92 | 0.90 | 1.00 | 0.78 |
| DEP | −0.89 | −0.74 | −0.82 | 0.90 | 0.93 | 0.78 | 1.00 |
| 2010–2014[c] | MHI | MFI | SEA | SEP | SED | SES | DEP |
| MHI | 1.00 | 0.85 | 0.85 | −0.88 | −0.91 | −0.82 | −0.86 |
| MFI | 0.85 | 1.00 | 0.86 | −0.84 | −0.89 | −0.90 | −0.73 |
| SEA | 0.85 | 0.86 | 1.00 | −0.91 | −0.90 | −0.95 | −0.80 |
| SEP | −0.88 | −0.84 | −0.91 | 1.00 | 0.95 | 0.94 | 0.89 |
| SED | −0.91 | −0.89 | −0.90 | 0.95 | 1.00 | 0.91 | 0.93 |
| SES | −0.82 | −0.90 | −0.95 | 0.94 | 0.91 | 1.00 | 0.78 |
| DEP | −0.86 | −0.73 | −0.80 | 0.89 | 0.93 | 0.78 | 1.00 |
| 2015–2019[d] | MHI | MFI | SEA | SEP | SED | SES | DEP |
| MHI | 1.00 | 0.84 | 0.82 | −0.83 | −0.86 | −0.77 | −0.82 |
| MFI | 0.84 | 1.00 | 0.85 | −0.80 | −0.92 | −0.91 | −0.76 |
| SEA | 0.82 | 0.85 | 1.00 | −0.87 | −0.87 | −0.93 | −0.78 |
| SEP | −0.83 | −0.80 | −0.87 | 1.00 | 0.93 | 0.87 | 0.89 |
| SED | −0.86 | −0.92 | −0.87 | 0.93 | 1.00 | 0.92 | 0.91 |
| SES | −0.77 | −0.91 | −0.93 | 0.87 | 0.92 | 1.00 | 0.77 |
| DEP | −0.82 | −0.76 | −0.78 | 0.89 | 0.91 | 0.77 | 1.00 |

[a] Correlation matrix based on 179 census tracts.

[b] Correlation matrix based on 179 census tracts.

[c] Correlation matrix based on 183 census tracts; 1 out of 184 census tracts (0.54%) were omitted due to missing data.

[d] Correlation matrix based on 183 census tracts; 1 out of 184 census tracts (0.54%) were omitted due to missing data.

Abbreviations: MHI, Median Household Income; MFI, Median Family Income; SEA, Socioeconomic Advantage [20]; SEP, Socioeconomic Position [21]; SED, Socioeconomic Deprivation [22]; SES, Socioeconomic Status [23]; DEP, Deprivation [24].

Table S44. Relationships between simple and composite measures of neighborhood socioeconomic status in the State of Virginia.

| 2000[a] | MHI | MFI | SEA | SEP | SED | SES | DEP |
|---|---|---|---|---|---|---|---|
| MHI | 1.00 | 0.97 | 0.91 | −0.90 | −0.89 | −0.92 | −0.78 |
| MFI | 0.97 | 1.00 | 0.93 | −0.90 | −0.90 | −0.95 | −0.78 |
| SEA | 0.91 | 0.93 | 1.00 | −0.93 | −0.94 | −0.97 | −0.86 |
| SEP | −0.90 | −0.90 | −0.93 | 1.00 | 0.96 | 0.96 | 0.91 |
| SED | −0.89 | −0.90 | −0.94 | 0.96 | 1.00 | 0.94 | 0.95 |
| SES | −0.92 | −0.95 | −0.97 | 0.96 | 0.94 | 1.00 | 0.84 |
| DEP | −0.78 | −0.78 | −0.86 | 0.91 | 0.95 | 0.84 | 1.00 |
| 2005–2009[b] | MHI | MFI | SEA | SEP | SED | SES | DEP |
| MHI | 1.00 | 0.95 | 0.91 | −0.90 | −0.90 | −0.90 | −0.78 |
| MFI | 0.95 | 1.00 | 0.92 | −0.90 | −0.89 | −0.93 | −0.76 |
| SEA | 0.91 | 0.92 | 1.00 | −0.94 | −0.93 | −0.97 | −0.85 |
| SEP | −0.90 | −0.90 | −0.94 | 1.00 | 0.96 | 0.97 | 0.92 |
| SED | −0.90 | −0.89 | −0.93 | 0.96 | 1.00 | 0.94 | 0.95 |
| SES | −0.90 | −0.93 | −0.97 | 0.97 | 0.94 | 1.00 | 0.86 |
| DEP | −0.78 | −0.76 | −0.85 | 0.92 | 0.95 | 0.86 | 1.00 |
| 2010–2014[c] | MHI | MFI | SEA | SEP | SED | SES | DEP |
| MHI | 1.00 | 0.96 | 0.91 | −0.90 | −0.91 | −0.90 | −0.78 |
| MFI | 0.96 | 1.00 | 0.93 | −0.92 | −0.92 | −0.94 | −0.79 |
| SEA | 0.91 | 0.93 | 1.00 | −0.95 | −0.93 | −0.97 | −0.86 |
| SEP | −0.90 | −0.92 | −0.95 | 1.00 | 0.97 | 0.98 | 0.92 |
| SED | −0.91 | −0.92 | −0.93 | 0.97 | 1.00 | 0.95 | 0.95 |
| SES | −0.90 | −0.94 | −0.97 | 0.98 | 0.95 | 1.00 | 0.88 |
| DEP | −0.78 | −0.79 | −0.86 | 0.92 | 0.95 | 0.88 | 1.00 |
| 2015–2019[d] | MHI | MFI | SEA | SEP | SED | SES | DEP |
| MHI | 1.00 | 0.96 | 0.91 | −0.87 | −0.91 | −0.89 | −0.79 |
| MFI | 0.96 | 1.00 | 0.94 | −0.89 | −0.93 | −0.94 | −0.79 |
| SEA | 0.91 | 0.94 | 1.00 | −0.93 | −0.93 | −0.97 | −0.86 |
| SEP | −0.87 | −0.89 | −0.93 | 1.00 | 0.95 | 0.96 | 0.91 |
| SED | −0.91 | −0.93 | −0.93 | 0.95 | 1.00 | 0.94 | 0.94 |
| SES | −0.89 | −0.94 | −0.97 | 0.96 | 0.94 | 1.00 | 0.86 |
| DEP | −0.79 | −0.79 | −0.86 | 0.91 | 0.94 | 0.86 | 1.00 |

[a] Correlation matrix based on 1,516 census tracts; 25 out of 1,541 census tracts (1.62%) were omitted due to missing data.

[b] Correlation matrix based on 1,501 census tracts; 40 out of 1,541 census tracts (2.60%) were omitted due to missing data.

[c] Correlation matrix based on 1,842 census tracts; 65 out of 1,907 census tracts (3.41%) were omitted due to missing data.

[d] Correlation matrix based on 1,832 census tracts; 75 out of 1,907 census tracts (3.93%) were omitted due to missing data.

Abbreviations: MHI, Median Household Income; MFI, Median Family Income; SEA, Socioeconomic Advantage [20]; SEP, Socioeconomic Position [21]; SED, Socioeconomic Deprivation [22]; SES, Socioeconomic Status [23]; DEP, Deprivation [24].

Table S45. Relationships between simple and composite measures of neighborhood socioeconomic status in the State of Washington.

| 2000[a] | MHI | MFI | SEA | SEP | SED | SES | DEP |
|---|---|---|---|---|---|---|---|
| MHI | 1.00 | 0.94 | 0.86 | −0.87 | −0.89 | −0.84 | −0.79 |
| MFI | 0.94 | 1.00 | 0.92 | −0.91 | −0.91 | −0.92 | −0.81 |
| SEA | 0.86 | 0.92 | 1.00 | −0.93 | −0.91 | −0.96 | −0.85 |
| SEP | −0.87 | −0.91 | −0.93 | 1.00 | 0.95 | 0.97 | 0.92 |
| SED | −0.89 | −0.91 | −0.91 | 0.95 | 1.00 | 0.92 | 0.96 |
| SES | −0.84 | −0.92 | −0.96 | 0.97 | 0.92 | 1.00 | 0.87 |
| DEP | −0.79 | −0.81 | −0.85 | 0.92 | 0.96 | 0.87 | 1.00 |
| 2005–2009[b] | MHI | MFI | SEA | SEP | SED | SES | DEP |
| MHI | 1.00 | 0.91 | 0.86 | −0.87 | −0.88 | −0.83 | −0.77 |
| MFI | 0.91 | 1.00 | 0.91 | −0.90 | −0.91 | −0.92 | −0.79 |
| SEA | 0.86 | 0.91 | 1.00 | −0.94 | −0.91 | −0.96 | −0.86 |
| SEP | −0.87 | −0.90 | −0.94 | 1.00 | 0.95 | 0.97 | 0.92 |
| SED | −0.88 | −0.91 | −0.91 | 0.95 | 1.00 | 0.92 | 0.95 |
| SES | −0.83 | −0.92 | −0.96 | 0.97 | 0.92 | 1.00 | 0.86 |
| DEP | −0.77 | −0.79 | −0.86 | 0.92 | 0.95 | 0.86 | 1.00 |
| 2010–2014[c] | MHI | MFI | SEA | SEP | SED | SES | DEP |
| MHI | 1.00 | 0.92 | 0.87 | −0.88 | −0.89 | −0.84 | −0.79 |
| MFI | 0.92 | 1.00 | 0.91 | −0.91 | −0.91 | −0.93 | −0.80 |
| SEA | 0.87 | 0.91 | 1.00 | −0.95 | −0.92 | −0.96 | −0.86 |
| SEP | −0.88 | −0.91 | −0.95 | 1.00 | 0.95 | 0.97 | 0.92 |
| SED | −0.89 | −0.91 | −0.92 | 0.95 | 1.00 | 0.92 | 0.95 |
| SES | −0.84 | −0.93 | −0.96 | 0.97 | 0.92 | 1.00 | 0.86 |
| DEP | −0.79 | −0.80 | −0.86 | 0.92 | 0.95 | 0.86 | 1.00 |
| 2015–2019[d] | MHI | MFI | SEA | SEP | SED | SES | DEP |
| MHI | 1.00 | 0.93 | 0.89 | −0.87 | −0.89 | −0.86 | −0.75 |
| MFI | 0.93 | 1.00 | 0.92 | −0.89 | −0.91 | −0.93 | −0.76 |
| SEA | 0.89 | 0.92 | 1.00 | −0.92 | −0.92 | −0.96 | −0.83 |
| SEP | −0.87 | −0.89 | −0.92 | 1.00 | 0.95 | 0.96 | 0.90 |
| SED | −0.89 | −0.91 | −0.92 | 0.95 | 1.00 | 0.93 | 0.94 |
| SES | −0.86 | −0.93 | −0.96 | 0.96 | 0.93 | 1.00 | 0.84 |
| DEP | −0.75 | −0.76 | −0.83 | 0.90 | 0.94 | 0.84 | 1.00 |

[a] Correlation matrix based on 1,313 census tracts; 5 out of 1,318 census tracts (0.38%) were omitted due to missing data.

[b] Correlation matrix based on 1,305 census tracts; 13 out of 1,318 census tracts (0.99%) were omitted due to missing data.

[c] Correlation matrix based on 1,432 census tracts; 26 out of 1,458 census tracts (1.78%) were omitted due to missing data.

[d] Correlation matrix based on 1,423 census tracts; 35 out of 1,458 census tracts (2.40%) were omitted due to missing data.

Abbreviations: MHI, Median Household Income; MFI, Median Family Income; SEA, Socioeconomic Advantage [20]; SEP, Socioeconomic Position [21]; SED, Socioeconomic Deprivation [22]; SES, Socioeconomic Status [23]; DEP, Deprivation [24].

Table S46. Relationships between simple and composite measures of neighborhood socioeconomic status in the State of West Virginia.

| 2000[a] | MHI | MFI | SEA | SEP | SED | SES | DEP |
|---|---|---|---|---|---|---|---|
| MHI | 1.00 | 0.91 | 0.86 | −0.89 | −0.92 | −0.84 | −0.93 |
| MFI | 0.91 | 1.00 | 0.92 | −0.90 | −0.94 | −0.94 | −0.88 |
| SEA | 0.86 | 0.92 | 1.00 | −0.93 | −0.93 | −0.97 | −0.91 |
| SEP | −0.89 | −0.90 | −0.93 | 1.00 | 0.94 | 0.94 | 0.93 |
| SED | −0.92 | −0.94 | −0.93 | 0.94 | 1.00 | 0.93 | 0.97 |
| SES | −0.84 | −0.94 | −0.97 | 0.94 | 0.93 | 1.00 | 0.87 |
| DEP | −0.93 | −0.88 | −0.91 | 0.93 | 0.97 | 0.87 | 1.00 |
| 2005–2009[b] | MHI | MFI | SEA | SEP | SED | SES | DEP |
| MHI | 1.00 | 0.87 | 0.83 | −0.87 | −0.91 | −0.80 | −0.88 |
| MFI | 0.87 | 1.00 | 0.86 | −0.84 | −0.92 | −0.89 | −0.81 |
| SEA | 0.83 | 0.86 | 1.00 | −0.90 | −0.90 | −0.94 | −0.83 |
| SEP | −0.87 | −0.84 | −0.90 | 1.00 | 0.91 | 0.91 | 0.90 |
| SED | −0.91 | −0.92 | −0.90 | 0.91 | 1.00 | 0.89 | 0.93 |
| SES | −0.80 | −0.89 | −0.94 | 0.91 | 0.89 | 1.00 | 0.79 |
| DEP | −0.88 | −0.81 | −0.83 | 0.90 | 0.93 | 0.79 | 1.00 |
| 2010–2014[c] | MHI | MFI | SEA | SEP | SED | SES | DEP |
| MHI | 1.00 | 0.89 | 0.84 | −0.85 | −0.91 | −0.79 | −0.90 |
| MFI | 0.89 | 1.00 | 0.86 | −0.84 | −0.93 | −0.87 | −0.81 |
| SEA | 0.84 | 0.86 | 1.00 | −0.90 | −0.90 | −0.93 | −0.81 |
| SEP | −0.85 | −0.84 | −0.90 | 1.00 | 0.90 | 0.92 | 0.85 |
| SED | −0.91 | −0.93 | −0.90 | 0.90 | 1.00 | 0.88 | 0.92 |
| SES | −0.79 | −0.87 | −0.93 | 0.92 | 0.88 | 1.00 | 0.74 |
| DEP | −0.90 | −0.81 | −0.81 | 0.85 | 0.92 | 0.74 | 1.00 |
| 2015–2019[d] | MHI | MFI | SEA | SEP | SED | SES | DEP |
| MHI | 1.00 | 0.88 | 0.85 | −0.83 | −0.91 | −0.81 | −0.85 |
| MFI | 0.88 | 1.00 | 0.88 | −0.83 | −0.92 | −0.90 | −0.78 |
| SEA | 0.85 | 0.88 | 1.00 | −0.89 | −0.91 | −0.95 | −0.81 |
| SEP | −0.83 | −0.83 | −0.89 | 1.00 | 0.90 | 0.92 | 0.87 |
| SED | −0.91 | −0.92 | −0.91 | 0.90 | 1.00 | 0.90 | 0.92 |
| SES | −0.81 | −0.90 | −0.95 | 0.92 | 0.90 | 1.00 | 0.77 |
| DEP | −0.85 | −0.78 | −0.81 | 0.87 | 0.92 | 0.77 | 1.00 |

[a] Correlation matrix based on 466 census tracts.

[b] Correlation matrix based on 463 census tracts; 3 out of 466 census tracts (0.64%) were omitted due to missing data.

[c] Correlation matrix based on 484 census tracts.

[d] Correlation matrix based on 479 census tracts; 5 out of 484 census tracts (1.03%) were omitted due to missing data.

Abbreviations: MHI, Median Household Income; MFI, Median Family Income; SEA, Socioeconomic Advantage [20]; SEP, Socioeconomic Position [21]; SED, Socioeconomic Deprivation [22]; SES, Socioeconomic Status [23]; DEP, Deprivation [24].

Table S47. Relationships between simple and composite measures of neighborhood socioeconomic status in the State of Wisconsin.

| 2000[a] | MHI | MFI | SEA | SEP | SED | SES | DEP |
|---|---|---|---|---|---|---|---|
| MHI | 1.00 | 0.94 | 0.88 | −0.87 | −0.84 | −0.86 | −0.72 |
| MFI | 0.94 | 1.00 | 0.91 | −0.90 | −0.88 | −0.92 | −0.76 |
| SEA | 0.88 | 0.91 | 1.00 | −0.94 | −0.94 | −0.96 | −0.87 |
| SEP | −0.87 | −0.90 | −0.94 | 1.00 | 0.94 | 0.96 | 0.88 |
| SED | −0.84 | −0.88 | −0.94 | 0.94 | 1.00 | 0.92 | 0.95 |
| SES | −0.86 | −0.92 | −0.96 | 0.96 | 0.92 | 1.00 | 0.83 |
| DEP | −0.72 | −0.76 | −0.87 | 0.88 | 0.95 | 0.83 | 1.00 |
| 2005–2009[b] | MHI | MFI | SEA | SEP | SED | SES | DEP |
| MHI | 1.00 | 0.90 | 0.88 | −0.87 | −0.87 | −0.84 | −0.75 |
| MFI | 0.90 | 1.00 | 0.88 | −0.87 | −0.89 | −0.91 | −0.76 |
| SEA | 0.88 | 0.88 | 1.00 | −0.94 | −0.92 | −0.96 | −0.85 |
| SEP | −0.87 | −0.87 | −0.94 | 1.00 | 0.95 | 0.96 | 0.90 |
| SED | −0.87 | −0.89 | −0.92 | 0.95 | 1.00 | 0.92 | 0.95 |
| SES | −0.84 | −0.91 | −0.96 | 0.96 | 0.92 | 1.00 | 0.84 |
| DEP | −0.75 | −0.76 | −0.85 | 0.90 | 0.95 | 0.84 | 1.00 |
| 2010–2014[c] | MHI | MFI | SEA | SEP | SED | SES | DEP |
| MHI | 1.00 | 0.90 | 0.88 | −0.87 | −0.86 | −0.85 | −0.75 |
| MFI | 0.90 | 1.00 | 0.90 | −0.89 | −0.89 | −0.92 | −0.77 |
| SEA | 0.88 | 0.90 | 1.00 | −0.95 | −0.93 | −0.96 | −0.87 |
| SEP | −0.87 | −0.89 | −0.95 | 1.00 | 0.96 | 0.97 | 0.92 |
| SED | −0.86 | −0.89 | −0.93 | 0.96 | 1.00 | 0.93 | 0.95 |
| SES | −0.85 | −0.92 | −0.96 | 0.97 | 0.93 | 1.00 | 0.87 |
| DEP | −0.75 | −0.77 | −0.87 | 0.92 | 0.95 | 0.87 | 1.00 |
| 2015–2019[d] | MHI | MFI | SEA | SEP | SED | SES | DEP |
| MHI | 1.00 | 0.91 | 0.89 | −0.86 | −0.88 | −0.85 | −0.79 |
| MFI | 0.91 | 1.00 | 0.91 | −0.88 | −0.91 | −0.93 | −0.79 |
| SEA | 0.89 | 0.91 | 1.00 | −0.93 | −0.94 | −0.95 | −0.88 |
| SEP | −0.86 | −0.88 | −0.93 | 1.00 | 0.94 | 0.95 | 0.92 |
| SED | −0.88 | −0.91 | −0.94 | 0.94 | 1.00 | 0.93 | 0.95 |
| SES | −0.85 | −0.93 | −0.95 | 0.95 | 0.93 | 1.00 | 0.87 |
| DEP | −0.79 | −0.79 | −0.88 | 0.92 | 0.95 | 0.87 | 1.00 |

[a] Correlation matrix based on 1,311 census tracts; 22 out of 1,333 census tracts (1.65%) were omitted due to missing data.

[b] Correlation matrix based on 1,304 census tracts; 29 out of 1,333 census tracts (2.18%) were omitted due to missing data.

[c] Correlation matrix based on 1,381 census tracts; 28 out of 1,409 census tracts (1.99%) were omitted due to missing data.

[d] Correlation matrix based on 1,375 census tracts; 34 out of 1,409 census tracts (2.41%) were omitted due to missing data.

Abbreviations: MHI, Median Household Income; MFI, Median Family Income; SEA, Socioeconomic Advantage [20]; SEP, Socioeconomic Position [21]; SED, Socioeconomic Deprivation [22]; SES, Socioeconomic Status [23]; DEP, Deprivation [24].

Table S48. Relationships between simple and composite measures of neighborhood socioeconomic status in the State of Wyoming.

| 2000[a] | MHI | MFI | SEA | SEP | SED | SES | DEP |
|---|---|---|---|---|---|---|---|
| MHI | 1.00 | 0.91 | 0.68 | −0.77 | −0.87 | −0.68 | −0.71 |
| MFI | 0.91 | 1.00 | 0.78 | −0.85 | −0.92 | −0.82 | −0.78 |
| SEA | 0.68 | 0.78 | 1.00 | −0.90 | −0.82 | −0.92 | −0.71 |
| SEP | −0.77 | −0.85 | −0.90 | 1.00 | 0.92 | 0.96 | 0.88 |
| SED | −0.87 | −0.92 | −0.82 | 0.92 | 1.00 | 0.87 | 0.88 |
| SES | −0.68 | −0.82 | −0.92 | 0.96 | 0.87 | 1.00 | 0.80 |
| DEP | −0.71 | −0.78 | −0.71 | 0.88 | 0.88 | 0.80 | 1.00 |
| 2005–2009[b] | MHI | MFI | SEA | SEP | SED | SES | DEP |
| MHI | 1.00 | 0.87 | 0.58 | −0.66 | −0.83 | −0.59 | −0.68 |
| MFI | 0.87 | 1.00 | 0.71 | −0.72 | −0.85 | −0.75 | −0.66 |
| SEA | 0.58 | 0.71 | 1.00 | −0.89 | −0.80 | −0.92 | −0.74 |
| SEP | −0.66 | −0.72 | −0.89 | 1.00 | 0.91 | 0.96 | 0.90 |
| SED | −0.83 | −0.85 | −0.80 | 0.91 | 1.00 | 0.86 | 0.91 |
| SES | −0.59 | −0.75 | −0.92 | 0.96 | 0.86 | 1.00 | 0.80 |
| DEP | −0.68 | −0.66 | −0.74 | 0.90 | 0.91 | 0.80 | 1.00 |
| 2010–2014[c] | MHI | MFI | SEA | SEP | SED | SES | DEP |
| MHI | 1.00 | 0.87 | 0.66 | −0.70 | −0.83 | −0.61 | −0.61 |
| MFI | 0.87 | 1.00 | 0.71 | −0.72 | −0.83 | −0.73 | −0.61 |
| SEA | 0.66 | 0.71 | 1.00 | −0.87 | −0.82 | −0.89 | −0.70 |
| SEP | −0.70 | −0.72 | −0.87 | 1.00 | 0.92 | 0.95 | 0.89 |
| SED | −0.83 | −0.83 | −0.82 | 0.92 | 1.00 | 0.86 | 0.85 |
| SES | −0.61 | −0.73 | −0.89 | 0.95 | 0.86 | 1.00 | 0.80 |
| DEP | −0.61 | −0.61 | −0.70 | 0.89 | 0.85 | 0.80 | 1.00 |
| 2015–2019[d] | MHI | MFI | SEA | SEP | SED | SES | DEP |
| MHI | 1.00 | 0.87 | 0.67 | −0.74 | −0.84 | −0.67 | −0.63 |
| MFI | 0.87 | 1.00 | 0.77 | −0.83 | −0.90 | −0.83 | −0.69 |
| SEA | 0.67 | 0.77 | 1.00 | −0.84 | −0.79 | −0.89 | −0.63 |
| SEP | −0.74 | −0.83 | −0.84 | 1.00 | 0.93 | 0.96 | 0.85 |
| SED | −0.84 | −0.90 | −0.79 | 0.93 | 1.00 | 0.87 | 0.87 |
| SES | −0.67 | −0.83 | −0.89 | 0.96 | 0.87 | 1.00 | 0.76 |
| DEP | −0.63 | −0.69 | −0.63 | 0.85 | 0.87 | 0.76 | 1.00 |

[a] Correlation matrix based on 125 census tracts; 2 out of 127 census tracts (1.57%) were omitted due to missing data.

[b] Correlation matrix based on 125 census tracts; 2 out of 127 census tracts (1.57%) were omitted due to missing data.

[c] Correlation matrix based on 129 census tracts; 3 out of 132 census tracts (2.27%) were omitted due to missing data.

[d] Correlation matrix based on 129 census tracts; 3 out of 132 census tracts (2.27%) were omitted due to missing data.

Abbreviations: MHI, Median Household Income; MFI, Median Family Income; SEA, Socioeconomic Advantage [20]; SEP, Socioeconomic Position [21]; SED, Socioeconomic Deprivation [22]; SES, Socioeconomic Status [23]; DEP, Deprivation [24].
